# Branched-chain amino acids and their metabolites decrease human and rat hepatic stellate cell activation

**DOI:** 10.1007/s11033-024-10027-4

**Published:** 2024-11-04

**Authors:** Maria Camila Trillos-Almanza, Magnolia Martinez Aguilar, Manon Buist-Homan, Nils Bomer, Karla Arevalo Gomez, Vincent E. de Meijer, Frederike G. I. van Vilsteren, Hans Blokzijl, Han Moshage

**Affiliations:** 1 Department of Gastroenterology and Hepatology, University Medical Centre Groningen, University of Groningen, Groningen, The Netherlands; 2 Department of Cardiology, University Medical Centre Groningen, University of Groningen, Groningen, The Netherlands; 3 Division of Hepato-Pancreato-Biliary Surgery and Liver Transplantation, Department of Surgery, University Medical Centre Groningen, University of Groningen, Groningen, The Netherlands; 4 Department of Laboratory Medicine, University Medical Centre Groningen, University of Groningen, Groningen, The Netherlands

**Keywords:** Branched-chain amino acids, BCAAs, Branched-chain keto acids, BCKAs, End-stage liver disease, Fibrosis, Liver cirrhosis, Hepatic stellate cells

## Abstract

**Background:**

End-stage liver diseases (ESLDs) are a significant global health challenge due to their high prevalence and severe health impacts. Despite the severe outcomes associated with ESLDs, therapeutic options remain limited. Targeting the activation of hepatic stellate cells (HSCs), key drivers of extracellular matrix accumulation during liver injury presents a novel therapeutic approach. In ESLDs patients, branched-chain amino acids (BCAAs, leucine, isoleucine and valine) levels are decreased, and supplementation has been proposed to attenuate liver fibrosis and improve regeneration. However, their effects on HSCs require further investigation.

**Objective:**

To evaluate the efficacy of BCAAs and their metabolites, branched-chain α-keto acids (BCKAs), in modulating HSCs activation in human and rat models.

**Methods:**

Primary HSCs from rats and cirrhotic and non-cirrhotic human livers, were cultured and treated with BCAAs or BCKAs to assess their effects on both preventing (from day 1 of isolation) and reversing (from day 7 of isolation) HSCs activation.

**Results:**

In rat HSCs, leucine and BCKAs significantly reduced fibrotic markers and cell proliferation. In human HSCs, the metabolite of isoleucine decreased cell proliferation around 85% and increased the expression of branched-chain ketoacid dehydrogenase. The other metabolites also showed antifibrotic effects in HSCs from non-cirrhotic human livers.

**Conclusion:**

BCAAs and their respective metabolites inhibit HSC activation with species-specific responses. Further research is needed to understand how BCAAs influence liver fibrogenesis. BCKAs supplementation could be a strategic approach for managing ESLDs, considering the nutritional status and amino acid profiles of patients.

**Graphical abstract:**

The antifibrotic effects of BCAAs and BCKAs in various conditions are depicted for human HSCs (left) and rat HSCs (right) The symbol ‘↓’ indicates a downregulation or a decrease. *α-SMA* alpha-smooth muscle actin, *BCAAs* branched-chain amino acids, *BCKAs* branched-chain keto acids, *HSCs* hepatic stellate cells, *KMV* α-keto-β-methylvalerate. Figure created with Biorender.com

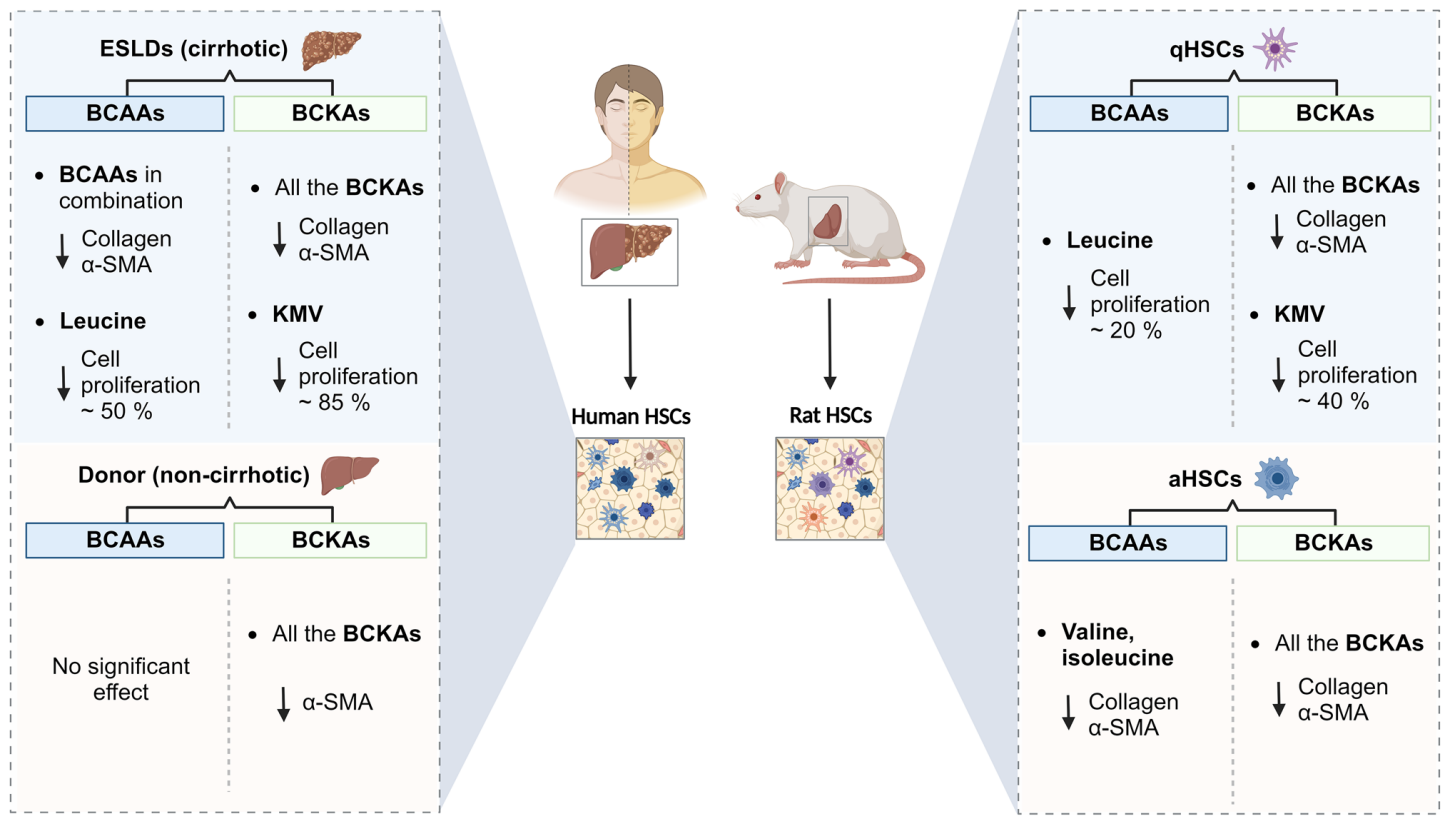

**Supplementary Information:**

The online version contains supplementary material available at 10.1007/s11033-024-10027-4.

## Introduction

End-stage liver diseases (ESLDs) represent a major global health challenge due to their high prevalence, severe health impact, high mortality and economic burden [1]. Despite their devastating consequences, therapeutic options for ESLDs are limited. Liver transplantation is often restricted by organ scarcity and resource limitations [2].

ESLDs are a consequence of chronic liver injury from various causes, including metabolic syndrome, excessive alcohol consumption, viral hepatitis and autoimmune disorders [3]. This leads to excessive extracellular matrix accumulation, scar tissue formation, and significant loss of liver function [3]. The development of liver fibrosis is influenced by hepatic stellate cells (HSCs) activation, as they drive the excessive deposition of extracellular matrix and remodeling enzymes [2, 4]. Preventing the progression of fibrogenesis is one strategy to reduce liver-related mortality.

Branched-chain Amino Acids (BCAAs), comprising leucine, valine and isoleucine, are essential amino acids in mammals that contribute to glucose homeostasis and protein synthesis [5]. They are metabolized into the branched-chain α-keto acids (BCKAs) α-ketoisocaproate (KIC), α-ketoisovalerate (KIV) and α-keto-β-methylvalerate (KMV), by branched-chain aminotransferases (BCAT) [5, 6]. The cytosolic isoform, BCAT1, is mostly expressed in brain and immune cells, and the mitochondrial isoform, BCAT2, is present in skeletal muscle, kidney, pancreas, colon, and to a lower extent in the liver [5, 6]. BCKAs are subsequently converted into branched-chain acyl-CoA esters by branched-chain α-keto acid dehydrogenase (BCKDH), mainly active in the liver [5, 6].

In ESLDs patients, the serum ratio of BCAAs to aromatic amino acids (Fischer ratio) is typically low [5, 7] associated with poor outcomes including hepatic encephalopathy, sarcopenia, and increased mortality [8, 9]. BCAAs supplementation has shown benefits in improving muscle mass, increasing plasma albumin levels, and reducing severe cirrhotic complications [5, 10, 11]. Beyond these clinical benefits, BCAAs may contribute to improving fibrosis regression and liver regeneration in animal models [12, 13], and inhibit the transforming growth factor β (TGF-β)-induced activation of LX-2 cells [14].

The evidence supporting BCAAs as a treatment for liver fibrosis primarily comes from in vivo and limited in vitro experiments. As a result, the overall effectiveness of BCAAs as a potential anti-fibrotic treatment, and which specific BCAA is most effective, remain incompletely understood. We hypothesized that BCAAs possess antifibrotic properties, and we aimed to assess the efficacy of BCAAs and their metabolites in reducing and preventing the activation of primary human and rat HSCs.

## Materials and methods

### Cell isolation and culture

Isolation and culture of human HSCs (hHSCs), rat HSCs (rHSCs), skeletal muscle (SKM) cells, and human cardiomyocytes, are described in Supplemental Materials and Methods.

### Experimental design

**Fig. 1:**
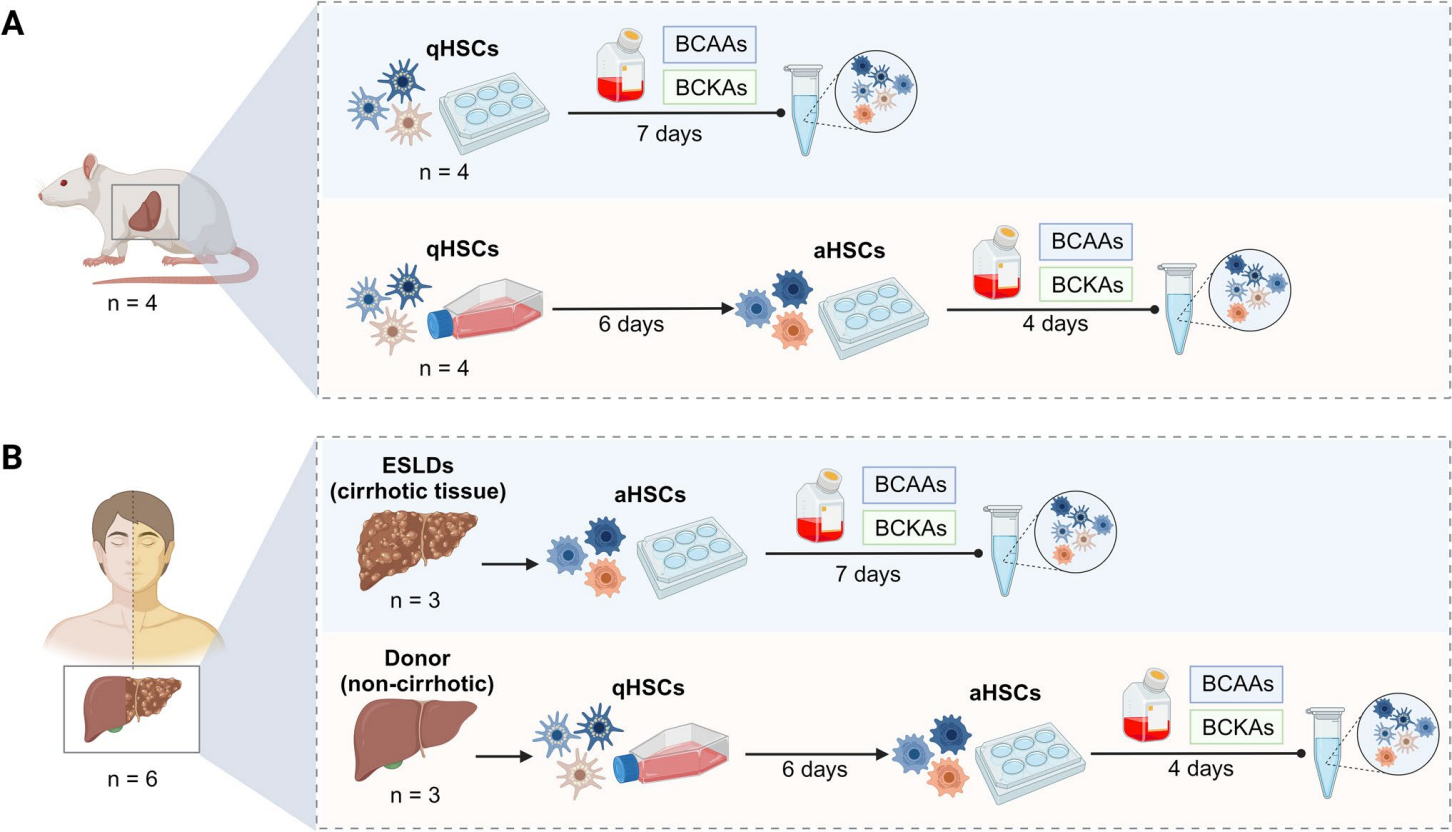
Methodology. HSCs were isolated from rat (**A**) and human (**B**) livers. Quiescent HSCs from rats were cultured in a medium enriched with BCAAs or BCKAs for 7 days, or cultured in a flask with standard IMDM medium for 6 days to achieve activation. Afterward, they were treated for 4 days with BCAAs- or BCKAs-enriched medium. For humans, HSCs were isolated from both cirrhotic and non-cirrhotic livers. HSCs from cirrhotic livers were cultured in a BCAAs- or BCKAs-enriched medium for 7 days, and HSCs from non-cirrhotic livers were cultured in a flask with standard IMDM medium for 6 days to achieve complete activation, and then treated for 4 days with a BCAAs- or BCKAs-enriched medium. *aHSCs* activated hepatic stellate cells, *BCAAs* branched-chain amino acids, *BCKAs* branched-chain keto acids, *ESLD* end-stage liver disease, *qHSCs* quiescent hepatic stellate cells. Figure created with Biorender.com

The experimental design is depicted in Fig. 1.

To evaluate whether BCAAs can inhibit the activation process of HSCs, quiescent rHSCs were cultured for 7 days in IMDM medium enriched with BCAAs, including L-Leucine (L8912, Sigma-Aldrich, Zwijndrecht, The Netherlands), L-Isoleucine (I7403, Sigma-Aldrich), L-Valine (V0513, Sigma-Aldrich), or the combination of the three in a 1:1:1 ratio (BCAAs) at a concentration of 15 mM.

To evaluate whether BCAAs can revert the activation process in HSCs, activated rHSCs (7 days after isolation) were treated for 96 h with BCAAs. Additional experiments were performed on rHSCs to corroborate these findings: rHSCs were cultured in conditioned medium from cardiomyocytes, containing the metabolites of BCAAs. Also, quiescent rHSCs were exposed to TGF-β (2.5 ng/mL) for 48 h to induce their activation in the presence of BCAAs.

hHSCs from cirrhotic livers were considered activated cells and cultured for 7 days with BCAAs. hHSCs from non-cirrhotic livers were first cultured in flasks for 6 days to induce activation and subsequently treated with BCAAs.

Regarding the experiments with BCKAs, both quiescent and activated rHSCs, as well as hHSCs from cirrhotic and non-cirrhotic livers, were treated with KIC (H60076.03, Thermo Fisher Scientific, Waltham, MA, USA), KMV (189,720,010, Thermo Fisher Scientific), KIV (198,978, Sigma-Aldrich), or their combination in a 1:1:1 ratio (BCKAs) at a concentration of 2 mM in culture medium.

### Laboratory techniques

Western blot analysis, RNA isolation, cDNA synthesis and RT-PCR, immunofluorescence microscopy, cell toxicity determination, and cell proliferation assays are described in the Supplemental Materials and Methods.

### Statistical analysis

GraphPad Prism 10.2.0 (Dotmatics, San Diego, CA, USA) was used for the graphs and the statistical analysis. Data are presented as means ± standard error of the mean. Statistical significance between groups was assessed using two-way ANOVA and *t*-tests, as appropriate. *P* values ≤ 0.05 were considered significant. Each experiment was repeated at least three times, using HSCs from different isolations.

### Ethical considerations

The Animal Ethics Committee at the University of Groningen (DEC-RUG) approved the protocols involving animal experiments (IvD protocol AVD10500202115139), conducted according to ARRIVE guidelines [15]. Informed consent was obtained from all patients. Human research was approved by the Medical Ethical Committee at UMCG, in accordance with Dutch laws, the Code of Conduct for responsibly handling of human tissue, and the Declaration of Helsinki guidelines [16].

## Results

### Branched-chain amino acids metabolism in rat hepatic stellate cells

**Fig. 2:**
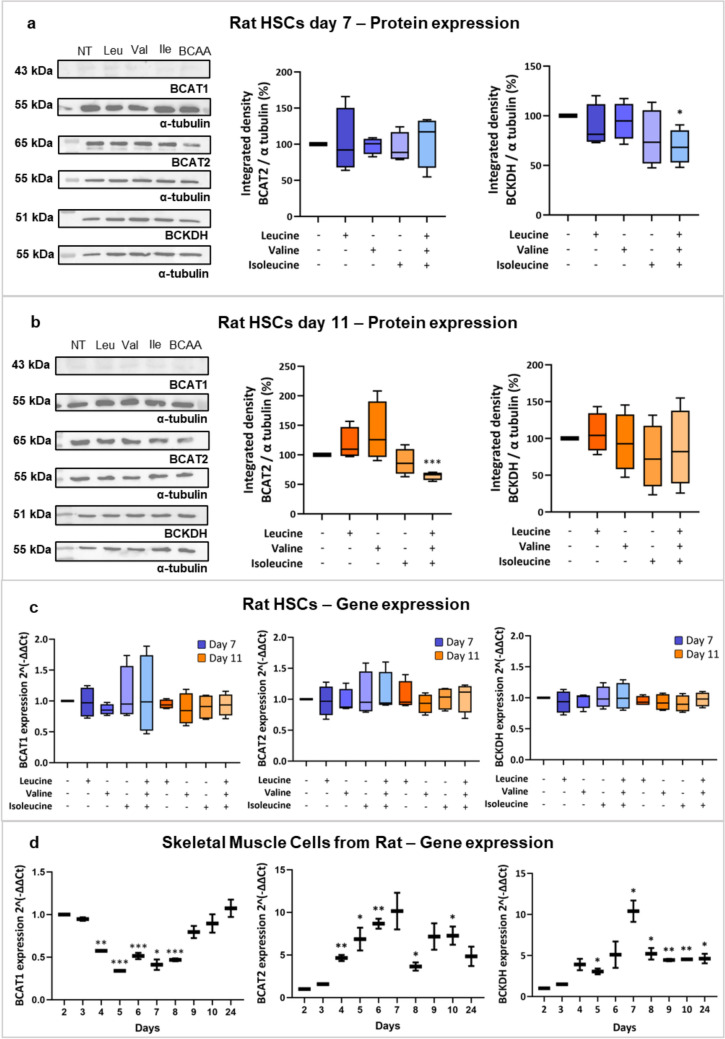
Expression of *BCAT* and *BCKDH* genes and proteins in HSCs and skeletal muscle tissues from rats. Representative Western blots and protein expression of BCAT2 and BCKDH in HSCs on day 7 (**a**) and day 11 (**b**) after treatment with leucine, valine, isoleucine, or a combination of the three BCAAs. Panel c shows the gene expression of *BCAT1, BCAT2*, and *BCKDH* in HSCs from rats, indicated as delta-delta Ct, at day 7 (blue bars) and day 11 (orange bars) of treatment with BCAAs as indicated. Panel d illustrates the gene expression levels, indicated as delta-delta Ct value of *BCAT1, BCAT2*, and *BCKDH* in skeletal muscle cells from rats after various culture times. *BCAAs* branched-chain amino acids, *BCAT* branched-chain amino acid transaminase, *BCKDH* branched-chain keto acid dehydrogenase, *Ile* Isoleucine, *Leu* Leucine, *Val* Valine. *p < 0.05; *** *p* < 0.001. Figure created with Biorender.com

Figure 2 illustrates the expression of BCAAs enzymes in rHSCs.

BCAT1 protein expression was weak in rHSCs on days 7 and 11, with or without BCAAs treatment (Fig. 2a and b). BCAT2 protein expression decreased by day 11 with BCAAs (Fig. 2b). BCKDH protein expression tended to decrease by day 11 (Fig. 2b) and was significantly lower on day 7 with BCAAs (Fig. 2a). BCAT2 was exclusively detected in rHSCs and SKM cells (Supplemental Fig. S1d).

Gene expression of *Bcat2* and *Bckdh* increased over time in SKM cells (Fig. 2d). After one week, *Bcat2* and *Bckdh* expression was lower in rHSCs compared to SKM cells (Supplemental Fig. S1a and b) and no differences were noted in the expression of these enzymes on days 7 or 11 with or without BCAAs (Fig. 2c) (Supplemental Fig. S1a). TGF-β upregulated *Bcat1* and *Bckdh* in rHSCs treated with isoleucine at day 7 (Supplemental Fig. S1c).

In Supplemental Figs. S2–S4) the effects of various culture times and BCAAs concentrations (Supplemental Figs. S3 and S4) on gene expression of *Bcat* and *Bckdh* were measured. By day 11, *Bcat1* and *Bcat2* expression tended to increase, while *Bckdh* expression significantly decreased (Supplemental Fig. S2). Gene expression of *Bcat1*, *Bcat2*, and *Bckdh* did not significantly change with varying BCAAs concentrations (Supplemental Figs. S3 and S4).

### Branched-chain amino acids and branched-chain α-keto acids inhibit fibrogenesis and cell proliferation on rat hepatic stellate cells

**Fig. 3:**
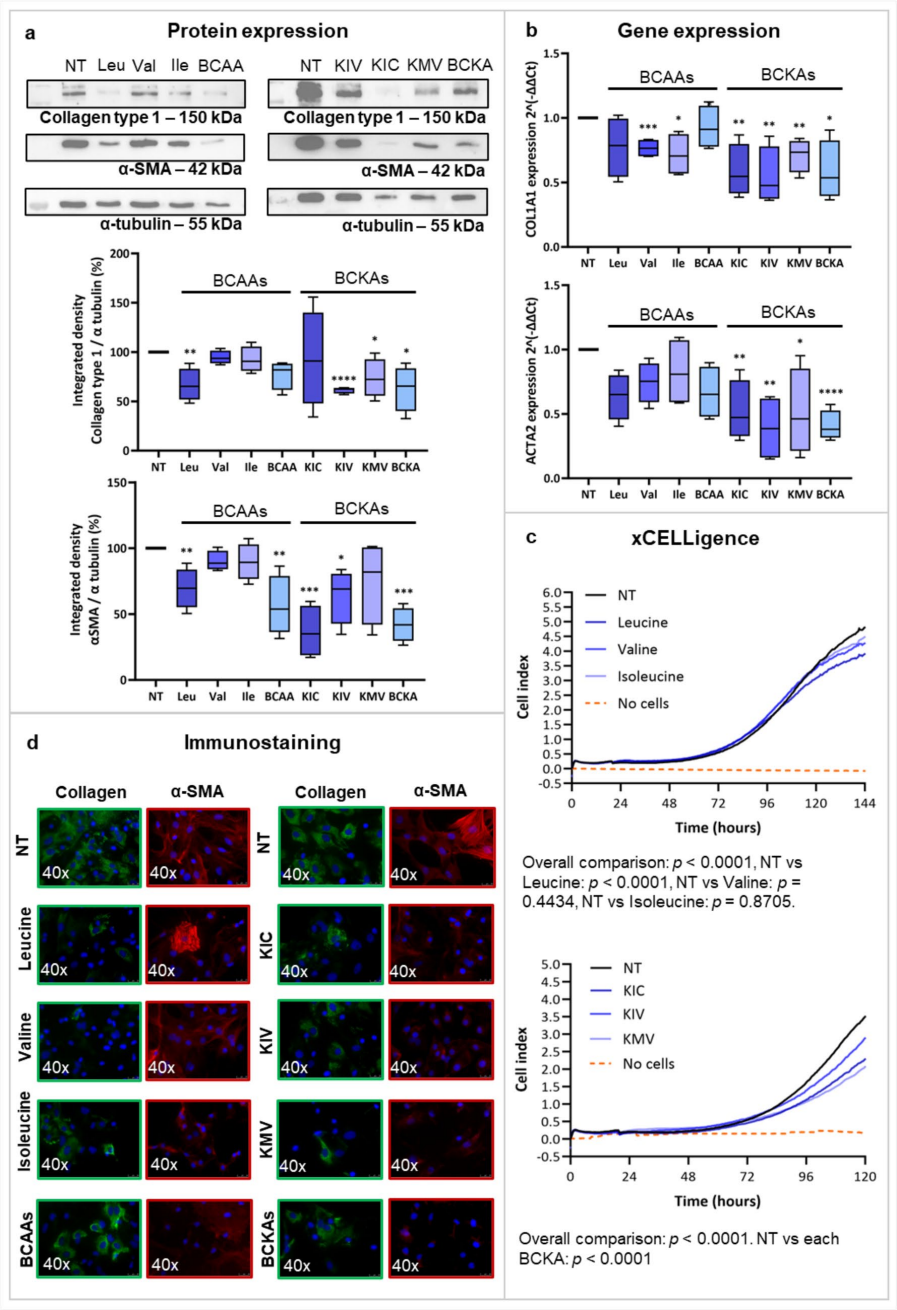
Effect of BCAAs and BCKAs on activation markers in rHSCs cultured with BCAAs from day 1 to 7. Panel A shows the protein expression of Collagen type 1 and α-SMA, given as the integrated density of the protein band divided by the integrated density of α-tubulin (used as a housekeeping protein), and then normalized to the control sample, in samples from rHSCs at day 7 after treatment with BCAAs and BCKAs as indicated. Panel B corresponds to the gene expression levels of *Col1a1* and *Acta2,* as delta-delta Ct with the housekeeping gene *36B4* in rHSCs at day 7. Panel C illustrates the cell proliferation in rHSCs treated with leucine, valine, isoleucine, KIC, KIV, KMV (blue lines), without any treatment (black line), or the control without cells (orange line). Panel D shows a representative immunofluorescence staining of Collagen type 1 (green) and α-SMA (red) under the indicated conditions. *α-SMA* alpha-smooth muscle actin, *BCAAs* branched-chain amino acids, *BCKAs* Branched-chain Keto Acids, *Ile* Isoleucine, *KIC* α-Ketoisocaproate, *KIV* α-Ketoisovalerate, *KMV* α-Keto-β-methylvalerate, *Leu* Leucine, *NT* Non-treatment, *Val* Valine. *p < 0.05; **p < 0.01; ***p < 0.001; ****p < 0.0001. Figure created with Biorender.com

BCAAs and BCKAs did not show toxicity after 72 h of treatment (data not shown). Leucine significantly reduced Collagen type 1 and α-SMA protein expression (*p* < 0.01), confirmed by immunofluorescence (Fig. 3a and d). Leucine and BCAAs significantly downregulated *Col1a1* expression in TGF-β activated rHSCs (Supplemental Fig. S5).

Valine and isoleucine downregulated *Col1a1* gene expression (*p* < 0.001 and *p* < 0.05, respectively), whereas leucine, valine, and BCAAs downregulated the expression of *Acta2* without reaching statistical significance (Fig. 3b). Leucine reduced rHSCs proliferation (*p* < 0.0001) (Fig. 3c).

KIV (*p* < 0.0001), KMV (*p* < 0.05), and BCKAs (*p* < 0.05) decreased protein expression of Collagen type 1 (Fig. 3a). Moreover, *Col1a1* was significantly downregulated under all conditions (Fig. 3b). α-SMA protein expression was reduced with KIC (*p* < 0.001), KIV (*p* < 0.05), and BCKAs (*p* < 0.001), and *Acta2* was significantly downregulated in all conditions (Fig. 3a and b). *Col1a1* and *Acta2* were also downregulated with increasing concentrations of BCKAs (Supplemental Fig. S6). BCKAs reduced Collagen type 1 and α-SMA immunofluoerescence and decreased cell proliferation for all the conditions (*p* < 0.0001) (Fig. 3c and d). After culturing rHSCs in conditioned medium from cardiomyocytes, only KIC downregulated *Col1a1* expression (*p* < 0.001), while the metabolites of the three BCAAs significantly downregulated *Acta2* expression (Supplemental Fig. S6a and b).

### Leucine, isoleucine, and their metabolites, reverse fibrogenesis on rat hepatic stellate cells

**Fig. 4:**
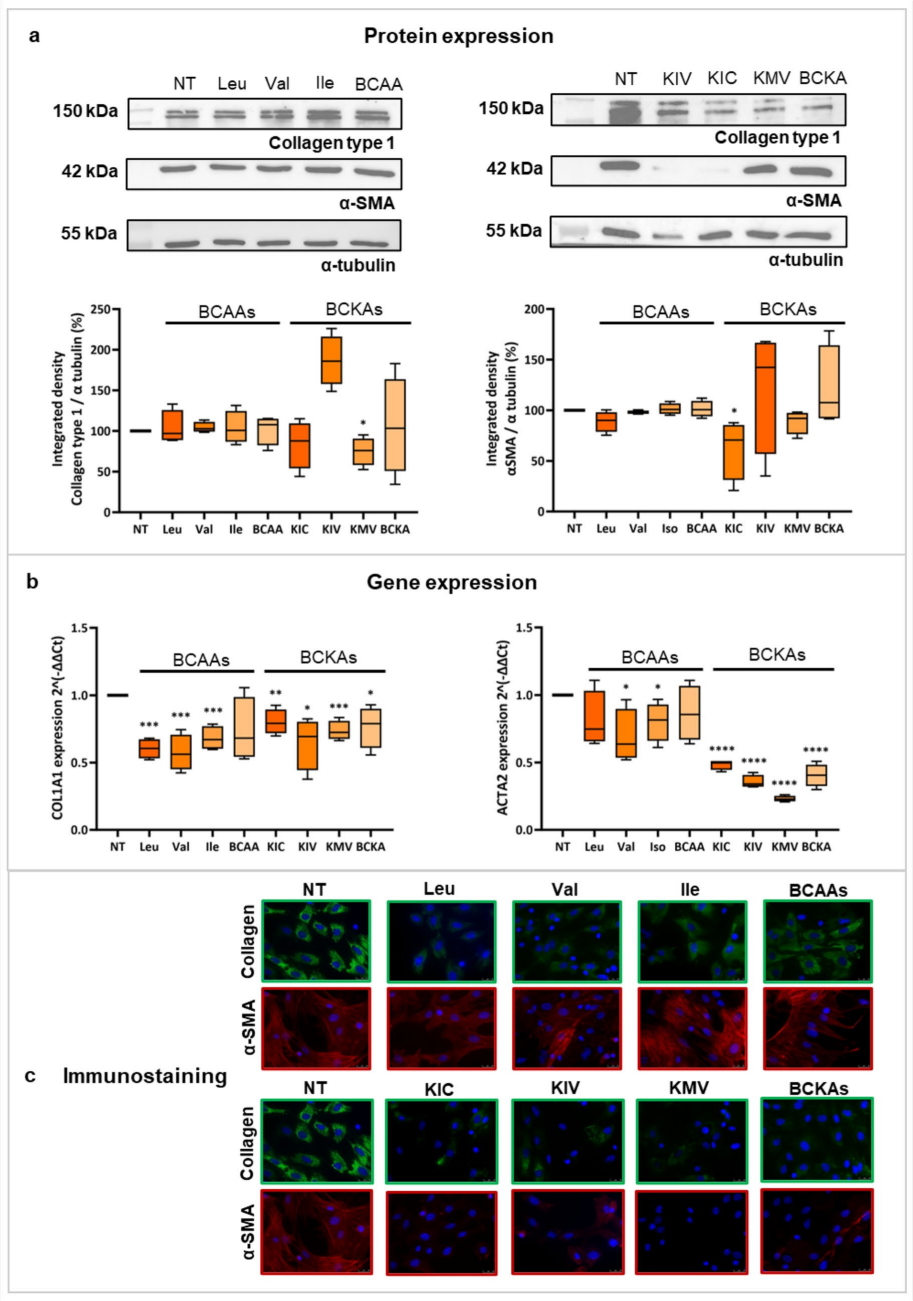
Effects of BCAAs and BCKAs on activation markers in rHSCs cultured with BCAAs from day 7 to 11. Panel A shows the protein expression of Collagen type 1 and α-SMA, given as the integrated density of the protein band divided by the integrated density of α-tubulin (used as a housekeeping protein), and then normalized to the control sample, in samples from rHSCs at day 11 after 4 days of treatment with BCAAs and BCKAs as indicated. Panel B shows the gene expression levels of *Col1a1* and *Acta2*, indicated as delta-delta Ct with the housekeeping gene *36b4*, under the indicated treatment conditions in rHSCs at day 11. Panel C indicates a representative immunofluorescence staining of Collagen type 1 (green) and α-SMA (red) under the indicated conditions. *α-SMA* alpha-smooth muscle actin, *BCAAs* Branched-chain Amino Acids, *BCKAs* Branched-chain Keto Acids, *Ile* Isoleucine, *KIC* α-Ketoisocaproate, *KIV* α-Ketoisovalerate, *KMV* α-Keto-β-methylvalerate, *Leu* Leucine, *NT* non-treatment, *Val* Valine. *p < 0.05; **p < 0.01; ***p < 0.001; ****p < 0.0001. Figure created with Biorender.com

BCAAs did not change protein expression of Collagen type 1 and α-SMA in already activated rHSCs (Fig. 4a).

However, at the gene expression level, the individual amino acids downregulated *Col1a1* (*p* < 0.001 for each amino acid) and tended to decrease *Acta2* expression (Fig. 4b). Immunofluorescence staining did not show a remarkable difference among the conditions (Fig. 4c).

KMV decreased the expression of Collagen Type 1 (*p* < 0.05) and KIC decreased α-SMA expression (*p* < 0.05) (Fig. 4a). At the gene expression level, BCKAs downregulated *Acta2* (*p* < 0.0001) and *Col1a1* (Fig. 4b). The immunofluorescence signal of Collagen and α-SMA also decreased (Fig. 4c). *Col1a1* expression was downregulated with increasing concentrations of KIC, KIV, KMV, and BCKAs (Supplemental Fig S8).

### Branched-chain amino acids metabolism in human hepatic stellate cells

**Fig. 5:**
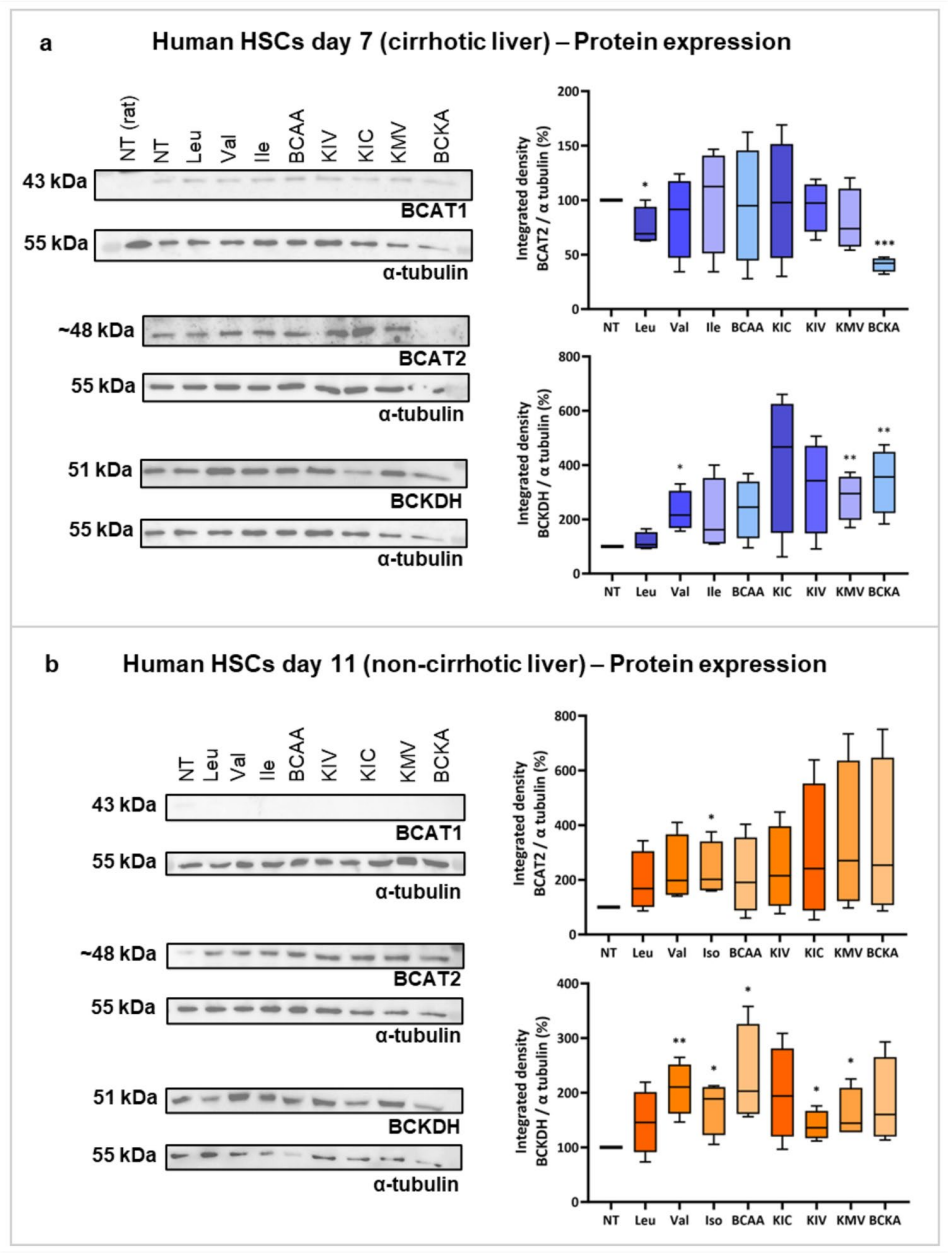
Protein expression of BCAT and BCKDH in human HSCs from cirrhotic and non-cirrhotic livers. Panel A illustrates the protein expression of BCAT and BCKDH in human HSCs isolated from cirrhotic livers (blue bars), treated with BCAAs or BCKAs as indicated. Panel B shows the protein expression of BCAT and BCKDH in human HSCs from non-cirrhotic livers (orange bars), also treated with BCAAs or BCKAs as indicated. *BCAAs* branched-chain amino acids, *BCAT* branched-chain amino acid transaminase, *BCKAs* branched-chain keto acids, *BCKDH* branched-chain keto acid dehydrogenase, *Ile* isoleucine, *KIC* α-ketoisocaproate, *KIV* α-ketoisovalerate, *KMV* α-keto-β-methylvalerate, *Leu* leucine, *Val* valine. *p < 0.05; **p < 0.01; ***p < 0.001. Figure created with Biorender.com

As shown in Fig. 5, BCAT1 protein was slightly expressed in both cirrhotic and non-cirrhotic hHSCs (Fig. 5a and b). BCKDH showed a similar pattern at the protein level in both types of hHSCs. In all other conditions, BCKDH tended to increase, and in some cases reached significance (Fig. 5a and b). In non-cirrhotic hHSCs, BCAT2 protein expression increased, particularly with isoleucine (*p* < 0.05) (Fig. 5b). In cirrhotic hHSCs, BCAT2 decreased with the use of leucine (*p* < 0.05) and BCKAs (*p* < 0.001).

### Branched-chain amino acids and branched-chain α-keto acids decrease the activation of human hepatic stellate cells from cirrhotic tissue

**Fig. 6:**
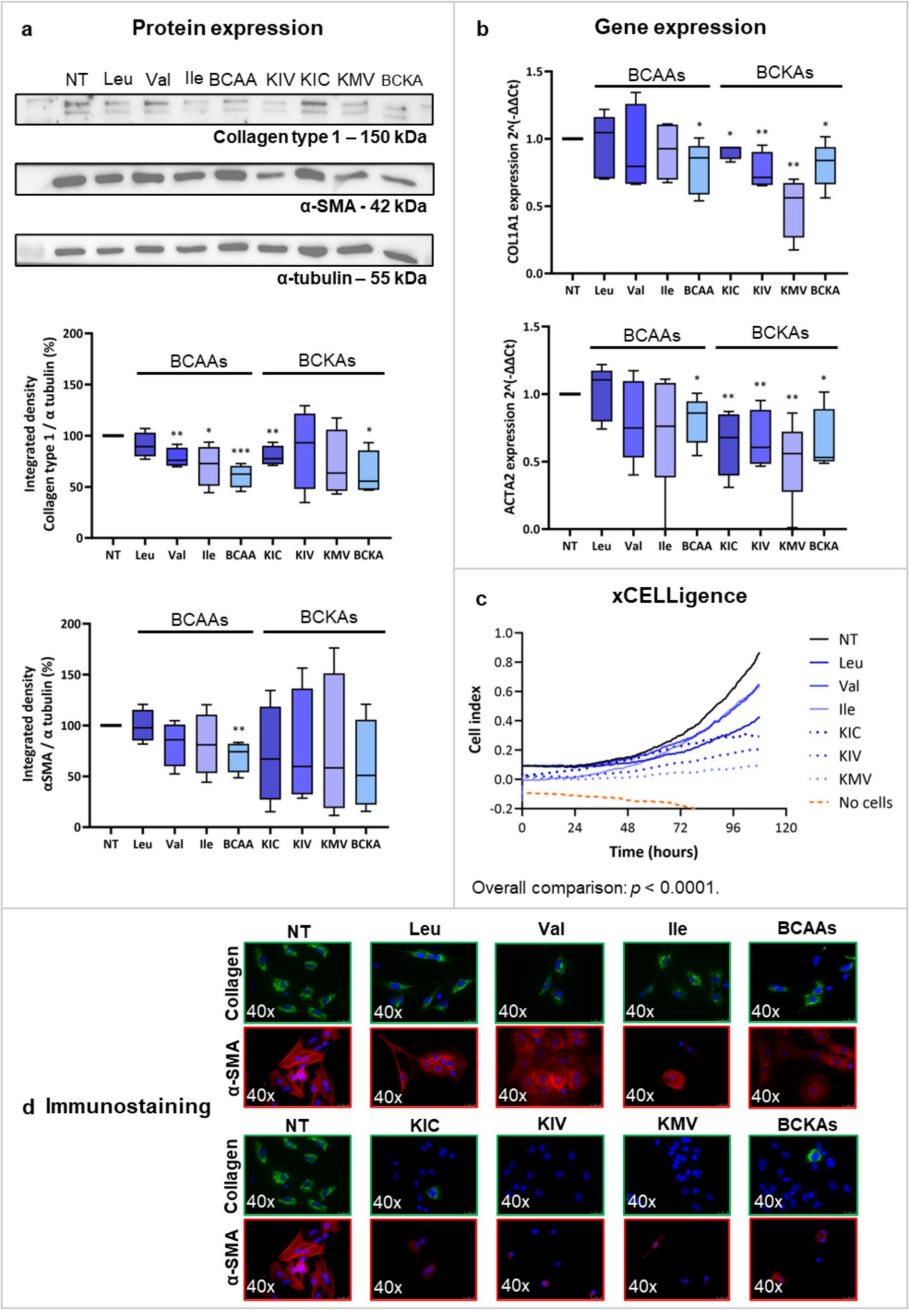
Effect of BCAAs and BCKAs on activation markers in hHSCs from cirrhotic tissue, cultured with BCAAs and BCKAs from day 1 to 7. Panel A shows the protein expression of Collagen type 1 and α-SMA, given as the integrated density of the protein band divided by the integrated density of α-tubulin (used as a housekeeping protein), and then normalized to the control sample when treating the cells with BCAAs and BCKAs as indicated. Panel B shows the gene expression levels of *COL1A1* and *ACTA2*, indicated as delta-delta Ct with the housekeeping gene *18S*, under the indicated treatment conditions. Panel C indicates the cell proliferation in hHSCs treated with leucine, valine, or isoleucine (continued blue lines), KIC, KIV, or KMV (intermittent blue lines), without any treatment (black line), or the controls without cells (orange line). Panel D shows the immunofluorescence staining of Collagen type 1 (green) and α-SMA (red) under the indicated conditions. *α-SMA* alpha-smooth muscle actin, *BCAAs* branched-chain amino acids, *BCKAs* branched-chain keto acids, *Ile* isoleucine; *KIC* α-ketoisocaproate, *KIV* α-ketoisovalerate, *KMV* α-keto-β-methylvalerate, *Leu* leucine, *NT* non-treatment, *Val* valine. *p < 0.05; **p < 0.01; ***p < 0.001. Figure created with Biorender.com

BCAAs and BCKAs did not induce cell toxicity after 72 h in hHSCs from cirrhotic tissue (data not shown). Supplementation with valine (*p* < 0.01), isoleucine (*p* < 0.05), BCAAs (*p* < 0.001), KIC (*p* < 0.01), and BCKAs (*p* < 0.05) reduced the protein expression of Collagen Type 1 (Fig. 6a).

At the gene expression level, BCAAs when used in combination, and BCKAs, individually or in combination, significantly downregulated the expression of *COL1A1* and *ACTA2* (Fig. 6b) as well as cell proliferation, especially with BCKAs (*p* < 0.0001) (Fig. 6c). Similarly, BCKAs clearly decreased the immunofluorescence staining for Collagen Type 1 and α-SMA (Fig. 6d).

### Branched-chain α-keto acids reverse activation of human hepatic stellate cells from non-cirrhotic tissue

**Fig. 7:**
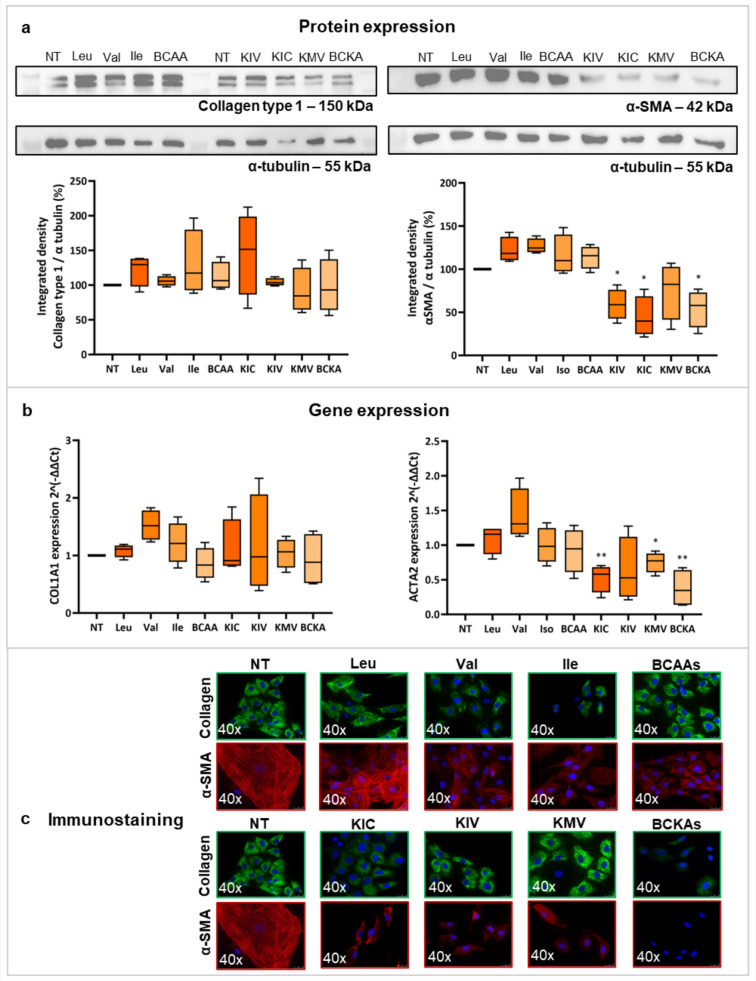
Effect of BCAAs and BCKAs on activation markers in hHSCs from non-cirrhotic tissue, cultured with BCAAs and BCKAs from day 7 to 11. Panel A shows the protein expression of Collagen type 1 and α-SMA, given as the integrated density of the protein band divided by the integrated density of α-tubulin (used as a housekeeping protein), and then normalized to the control sample, when treating the cells with BCAAs and BCKAs as indicated. Panel B shows the gene expression levels of *COL1A1* and *ACTA2*, indicated as delta-delta Ct with the housekeeping gene *18S*, under the indicated treatment conditions. Panel C indicates the immunofluorescence staining of Collagen type 1 (green) and α-SMA (red) under the indicated conditions. *α-SMA* alpha-smooth muscle actin, *BCAAs* branched-chain amino acids, *BCKAs* branched-chain keto acids, *Ile* isoleucine, *KIC* α-ketoisocaproate, *KIV* α-ketoisovalerate, *KMV* α-keto-β-methylvalerate, *Leu* leucine, *NT* non-treatment, *Val* valine. *p < 0.05; **p < 0.01. Figure created with Biorender.com

Treatment with BCKAs significantly downregulated expression of *ACTA2* and its associated protein α-SMA in hHSCs from non-cirrhotic tissue (Fig. 7a and b). Treatment with BCAAs either increased or did not modify the expression of this gene and protein (Fig. 7a and b).

Collagen type 1 protein expression was not affected by BCAAs, while there was a non-significant tendency to decrease Collagen type 1 when treating hHSCs with KMV or BCKAs (Fig. 7a). At the gene expression level, valine increased the expression of *COL1A1*, and both BCAAs and BCKAs showed a non-significant tendency to downregulate this gene (Fig. 7b). The immunostaining reflects these findings, showing a decrease in the fluorescence of α-SMA in hHSCs supplemented with KIC, KIV, KMV, and BCKAs. However, other conditions did not show clear differences (Fig. 7c).

In summary, different antifibrogenic effects were documented according to the condition and species; leucine and KMV were the most effective in inhibiting cell proliferation in both human and rat HSCs, and the strongest effects were shown with the use of BCKAs.

## Discussion

Preventing or reversing HSCs activation emerges as a promising strategy to treat ESLDs [17, 18]. Our study is the first to evaluate whether BCAAs (or their metabolites) can reduce or reverse the activation process in primary cultures of HSCs from humans and rats. We demonstrate that BCAAs, particularly their metabolites, exhibited (species-specific) antifibrogenic effects on both rat and human HSCs.

BCAAs supplementation is recommended in international guidelines for ESLDs patients to improve their nutritional status and hepatic encephalopathy [19, 20]. However, its benefits on liver function and disease state are inconsistent [21, 22]. These discrepancies arise from variations in BCAAs administration protocols, which highlight the importance of identifying the optimal characteristics for BCAAs supplementation at the animal and cellular levels before translating the findings into human research.

In previous studies using murine models, the combined use of BCAAs attenuated liver fibrosis through inhibition of the TGF-β1 signaling pathway by downregulating several key pathway components, including SMAD-4, P-SMAD3L, TIMP-1, PDGFRβ, p-ERK, and COL1A2 [12–14, 23]. Takegoshi et al. [13] reported that the administration of BCAAs in a high-fat diet-induced non-alcoholic steatohepatitis model demonstrated anti-fibrotic effects. In this study, mTORC1 activation inhibited TGF-β1 signaling in LX-2 and Huh-7 cells treated with BCAAs, suggesting a regulatory relationship between these pathways. This finding was further validated by Lee HL et al. [14], who reported that BCAAs treatment resulted in decreased activation of both Smad-dependent and Smad-independent p38 mitogen-activated protein kinase signaling pathways in LX-2 cells. Similarly, we also found in our in vitro study that BCAAs, particularly leucine, reduced the fibrogenic process in rHSCs when stimulated with TGF-β. However, valine and isoleucine did not show the same antifibrotic effects, suggesting that each BCAA may activate different cellular pathways.

Crosstalk between mTOR and TGF-β1 has been explored in various models, including primary human lung fibroblasts, human pterygium fibroblasts and NRK-49F cells. These studies indicate that mTOR signaling plays a crucial role in TGF-β1-induced α-SMA and collagen gene expression, specifically through an AKT-independent, Rictor/mTORC2-dependent pathway [24–26]. Conversely, some researchers suggest that leucine acts as a pro-oxidant in hepatic stellate cells via NADPH oxidase and mitochondrial reactive oxygen species production. These effects can activate insulin receptor/IGF-IR and mTOR signaling pathways, ultimately leading to translational regulation of collagen synthesis mediated by eIF4E released by 4E-BP1 or Mnk-1 [27].

When rHSCs were treated from their quiescent state, leucine and the combination of BCAAs also showed the strongest reducing effect on fibrogenic markers at the protein level, but not at the gene expression level. This finding could be explained by the known activation of the PI3K/Akt/mTOR pathway by leucine [28, 29]. Through the activation of this pathway, leucine could enhance specific protein synthesis pathways involved in cellular quiescence that mitigate factors leading to a myofibroblast-like state, for example, by increasing the synthesis of eIF4E-binding protein as demonstrated in other cell types [14, 30]. Another possible explanation is that the activation of mTOR could also lead to post-transcriptional regulation, particularly in splicing and polyadenylation [31], resulting in the antifibrotic effect of leucine at the protein level.

With activated cells, BCAAs did not demonstrate a clear antifibrotic effect, since their effects at the gene level did not translate into corresponding changes at the protein level. This can be explained by a lower activity of the BCAT2 enzyme when culturing the rHSCs until day 11 with BCAAs, due to a possible feedback inhibition or protein degradation over time, and by the reduced culturing time with BCAAs in comparison to the quiescent rHSCs. Increasing the culture time of these cells should be considered to evaluate whether the BCAAs can have antifibrotic effects on aHSCs.

The activity of the BCAT enzyme, responsible for the first step in BCAAs metabolism, is low in the liver [5, 6]. We did not find protein expression of BCAT1 in rHSC; however, we did observe expression of BCAT2, which could indicate that there was at least partial metabolism of BCAAs, resulting in the antifibrotic effect mentioned above. Using our in vitro approach, we evaluated the effects of individual BCKAs on HSCs activation, bypassing the first step of metabolism. Moreover, we used conditioned medium from cardiomyocytes containing the metabolic products of BCAAs. This is important because the liver does contain significant BCKDH activity, making this a more realistic condition. Our findings demonstrate clear inhibitory effects of BCKAs on activation markers in quiescent and activated rHSCs.

One of the unique characteristics of our study is the use of primary hHSCs, which is a significant advantage in comparison to cell lines. We observed that BCAAs and especially BCKAs, in particular KMV, significantly reduced the activation markers, including proliferation, in hHSC from cirrhotic livers. In hHSCs from non-cirrhotic livers, KIC and BCKAs were the most important down-regulators of fibrogenic genes, although the response at the protein level was not significant, possibly due to the shorter culturing time. Differences in response between liver donors and patients with ESLD may be attributed to variations in metabolic enzyme expression due to liver disease, as well as to genetic predispositions and comorbidities. Nevertheless, the antifibrotic effects of BCKAs were consistently evident across different subjects and conditions.

Species-specific variations in metabolism can account for the observed differences in response between rHSCs and hHSCs. BCAT activity in rat tissues, including muscle, heart, brain, kidney, and pancreas, is approximately two- to ten-fold higher than in human tissues, except for the liver, in which BCAT activity is higher in humans [32]. In human tissues, the highest activity of BCKDH is found in the kidney, followed by that in the liver, brain, and heart, but it is lower than the enzyme activity in rat [32]. BCAT2 protein expression decreases after treatment with BCAAs in rHSCs at day 11, but not at day 7, despite higher gene expression of BCAT2 at day 11. This phenomenon could be due to protein degradation or regulatory shutdown of enzymes at saturation to maintain metabolic balance. Further experimental validation, such as time-course studies of protein and mRNA levels, treatments with proteasome inhibitors, or assays to measure mRNA stability and translation efficiency, is necessary to explain the observed decrease in BCAT2 protein expression by day 11. In contrast, BCAT2 protein expression in hHSCs from non-cirrhotic livers showed a tendency to increase under prolonged culture conditions in the presence of BCAAs/BCKAs, whereas hHSCs from cirrhotic tissue either did not alter or decrease BCAT2 expression, especially in the case of leucine and BCKAs.

Another difference in BCAAs metabolism between humans and rats may be related to differences in the expression and regulation of BCKDH. BCKDH was consistently increased when using BCAAs and BCKAs in both hHSCs from both cirrhotic and non-cirrhotic liver tissue, while in rHSCs it decreased. In humans, the higher expression of the enzyme might indicate a higher rate of BCAAs catabolism in response to BCAAs and BCKAs due to different regulatory mechanisms in hHSCs that favor the activation of BCKDH. For instance, human cells might increase BCKDH expression to enhance energy production or to manage increased levels of BCAAs and their keto-derivatives more efficiently, especially under conditions simulating metabolic stress or therapeutic interventions, which can be reflected in different antifibrotic responses to the BCAAs.

In the current study, we described the metabolism of BCAAs in HSCs from humans with or without ESLD, and in rats. However, we did not investigate the specific molecular pathways leading to the antifibrotic effect. The effects of BCAAs and BCKAs on HSCs could result from various mechanisms, such as (I) activation of the PI3K/Akt/mTOR pathway, (II) inhibition of the TGF-β1 pathway, by affecting both the Smad and Smad-independent p38 MAPK signaling pathways [14], and (III) the effect of BCKAs metabolism on the mitochondria, which can alter the cellular redox state [33, 34], leading to changes in oxidative stress levels within HSCs and influencing their activation state and protein synthesis. Thus, further research should focus on these potential mechanisms.

From a clinical perspective, BCAAs supplementation is a relatively safe and recommended strategy that improves the nutritional status of patients with ESLDs and may be beneficial for those with hepatic encephalopathy when standard treatments are insufficient. Nevertheless, considering BCAAs as a therapy to prevent fibrosis development in patients with steatotic liver disease, or as a treatment for ESLDs at any stage, still requires further investigation. Not all patients would benefit from the supplementation, since elevated BCAAs serum levels could lead to metabolic complications [6, 35] and only patients with low levels of circulating BCAAs might be candidates for supplementation. Additionally, supplementation with BCKAs might be the preferred method to achieve an effect in these patients, as sarcopenia and poor nutritional states, which are common in patients with ESLDs, can alter the initial step in BCAAs metabolism. Before translating this into clinical practice, more knowledge regarding BCAAs and their metabolism, implications for health, and actual benefits should be considered.

## Conclusion

Our study provides an extensive overview of the antifibrotic potential of BCAAs and BCKAs, across various conditions, by mitigating the activation of HSCs from both humans and rats, primarily through the activity of BCKDH in the liver. We observed that each amino acid, and its metabolite, exhibited antifibrotic responses specific to the condition: in rats, leucine demonstrated the most effective results. In humans, KIC showed promising results in HSCs from healthy livers. Conversely, in human HSCs from patients with ESLDs, KMV, a metabolite of isoleucine, was more effective in enhancing the expression of BCKDH. The supplementation of BCKAs could potentially be a strategic approach in the future to reverse fibrogenesis in patients with ESLDs.

## Supplementary Information

Below is the link to the electronic supplementary material.

Supplementary file1 (PDF 3446 KB)

## Data Availability

Data is available from the corresponding author upon reasonable request and with the appropriate ethical approval.

## Abbreviations

ACTA2: Alpha-actin-2
BCAAs: Branched-chain amino acids
BCAT: Branched-chain aminotransferases
BCKAs: Branched-chain α-keto acids
BCKDHs: Branched-chain ketoacid dehydrogenase
COL1A1: Collagen, type 1, alpha 1
ESLD: End-stage liver disease
FCS: Fetal calf serum
hHSCs: Human hepatic stellate cells
HSCs: Hepatic stellate cells
KIC: α-Ketoisocaproate
KIV: α-Ketoisovalerate
KMV: α-Keto-β-methylvalerate
rHSCs: Rat hepatic stellate cells
SKM: Skeletal muscle
TGF-β: Transforming growth factor β
UMCG: University Medical Centre Groningen
α-SMA: Alpha smooth muscle actin
