# Supplementary material for: Branched-chain amino acids and their metabolites decrease human and rat hepatic stellate cell activation
Source: Mol Biol Rep. 2024 Nov 4;51(1):1116. doi: 10.1007/s11033-024-10027-4 (PMC11534903; doi:10.1007/s11033-024-10027-4)

# BRANCHED-CHAIN AMINO ACIDS AND THEIR METABOLITES DECREASE HUMAN AND RAT HEPATIC STELLATE CELL ACTIVATION

**Molecular Biology Reports**

Maria Camila Trillos-Almanza*, Magnolia Martinez Aguilar, Manon Buist-Homan, Nils Bomer, Karla Arevalo Gomez, Vincent E. de Meijer, Frederike G.I. van Vilsteren, Hans Blokzijl, Han Moshage.

*Department of Gastroenterology and Hepatology, University Medical Centre Groningen, University of Groningen, Groningen, The Netherlands. Correspondence: m.c.trillos.almanza@umcg.nl, a.j.moshage@umcg.nl, Tel: +31-50-3616161, Fax: +31-50-3619306.

## SUPPLEMENTAL MATERIALS AND METHODS

### Human tissue and isolation of human hepatic stellate cells

Human liver tissue of approximately 40 grams was collected from the liver explant of 3 patients with ESLDs who underwent liver transplantation, and from 3 non-cirrhotic human donor livers. The procedures were done in accordance with the experimental protocols at the University Medical Centre Groningen (UMCG). Liver tissues were stored in University of Wisconsin preservation solution (4 °C), and used for HSCs isolation within 12 hours following the resection. The liver wedges were washed with Hank's Balanced Salt Solution (HBSS) without calcium and magnesium (14175095, Thermo Fisher Scientific, Waltham, MA, USA) to remove excess blood, and then homogenized. The tissue was digested with a solution containing 0.01 % Collagenase P (11213857001, Roche, Almere, the Netherlands), 0.05 % Pronase (107433, Merck, Amsterdam, the Netherlands), and 0.001 % DNAse I (10104159001, Merck), in a shaking water bath at 37 °C for 45 minutes. Human HSCs (hHSCs) were isolated with a density gradient centrifugation containing 40 % OptiPrep ™ (1893, Progen, Heidelberg, Germany), as previously described by Rombouts K, et al. [1]. The average yield was approximately 0.4 to 0.51 ×10^6 cells per gram of liver tissue.

### Animal tissue and isolation of rat hepatic stellate cells

Rat HSCs (rHSCs) were isolated from pathogen-free male Wistar rats (Charles River Laboratories Inc., Wilmington, MA, USA) weighing 350 – 450 grams. Animals were housed under standard laboratory conditions with free access to standard laboratory chow diet and water. The rats were anesthetized with

isoflurane, ketamine, and dexmedetomidine. The livers were perfused via the portal vein with a buffer containing Pronase E (107433, Merck) and Collagenase P (11213857001, Roche) to digest the tissue. rHSCs were isolated using a density gradient centrifugation with a 13 % Nycodenz (18003, Serumwerk Bernburg AG, Bernburg, Germany) solution, as previously described [2, 3]. The average yield was approximately 0.84 to 1.33 ×10^6 cells per gram of liver tissue.

### Hepatic stellate cell culture conditions

Both hHSCs and rHSCs were cultured in a humidified atmosphere of 5 % $CO_2$ at 37 °C in Iscove's Modified Dulbecco's Medium (IMDM) with Glutamax (12440053, Thermo Fisher Scientific), supplemented with 20 % heat-inactivated fetal calf serum (FCS) (10082147, Thermo Fisher Scientific), 1 % sodium pyruvate (11360070, Thermo Fisher Scientific), 1 % MEM non-essential amino acids (11140050, Thermo Fisher Scientific), Penicillin/Streptomycin/Amphotericin B (15240062, Thermo Fisher Scientific). Primary HSCs were cultured on plastic for seven days to induce their spontaneous activation. For experiments with quiescent rHSCs and hHSCs from cirrhotic tissue, cells were cultured in 6-well plates and the treatment began 24 hours after isolation. For activated rHSCs and hHSCs from non-cirrhotic tissue, cells were cultured for 6 days in T-75 flasks to obtain complete activation before starting the treatments.

### Skeletal muscle cell isolation and culture

Skeletal muscle cells (SKM cells), encompassing individual myofibers and their satellite cells, were isolated from the extensor digitorum longus muscle of the same male Wistar rats used for rHSCs isolation, immediately after their death. The tissue was digested in a solution containing 0.2 % Collagenase type I (43E23601, Sigma-Aldrich, Zwijndrecht, The Netherlands), followed by sequential washing of myofibers in Petri dishes, under controlled conditions at 37 °C and careful handling to preserve fiber integrity before culturing, as described before [4]. SKM cells were cultured in 6-well plates at different time points (2 - 10, and 23 days) in Dulbecco's modified Eagle's medium (DMEM) with Glutamax (10564011, Thermo Fisher Scientific), supplemented with 20 % FCS (10082147, Thermo Fisher Scientific) and 1 % Penicillin/Streptomycin/Amphotericin B (15240062, Thermo Fisher Scientific).

### Cardiomyocytes culture conditions

Human Pluripotent Stem Cell (hPSCs) were maintained in TeSR-E8 medium (05990, StemCells technologies, Cologne, Germany) on a Geltrex ®-coated surface (A1413301, Thermo Fisher Scientific) under controlled conditions at 37 °C, 5 % $CO_2$ and 100 % humidity. The medium was refreshed daily. Differentiation to cardiomyocytes was achieved as described previously [5–7] and resulted in > 99 % pure spontaneously beating cardiomyocytes. Experiments were typically started at day 20 from the start of differentiation, after dissociating the cells to fit the experimental set-up in CDM3 medium [8] which was refreshed every other day. To achieve BCAAs metabolism by the hPSC-derived cardiomyocytes (hPSC-CMs), either 15 mM L-Leucine (L8912, Sigma-Aldrich), 15 mM L-Isoleucine (I7403, Sigma-Aldrich), 15 mM L-Valine (V0513, Sigma-Aldrich), or the combination of all three was added to fresh CDM3 culture medium. After 6 hours, the specified conditioned media was collected and stored at – 20 °C.

**Western blot analysis**

At the end of the experiments, cells were collected using lysis buffer and protein lysates were prepared through four cycles of freezing at –196 °C and thawing at 37 °C, followed by a centrifugation step for 15 minutes at 12000 g. Protein concentrations were determined using the Bio-Rad protein assay (5000111, Bio-Rad Laboratories, Veendedaal, The Netherlands). Western blot analysis was conducted on the cell lysates by loading 40 µg of protein on 10 % SDS-PAGE gels and using semidry blotting for protein transfer onto the nitrocellulose membrane. 0.1 % w/v Ponceau S stain (P7170, Sigma-Aldrich) was used to confirm the transfer of proteins. Membranes were blocked with 5 % Bovine Serum Albumin (BSA) (A3059, Sigma-Aldrich) and incubated overnight with the primary antibodies listed in Table 1. α tubulin was used as housekeeping protein. Protein bands were visualized using a Chemidoc MR system (Bio-Rad Laboratories), and band intensities were quantified using ImageJ 1.53 m software (National Institutes of Health, USA).

**Table 1.** Primary and secondary antibodies used for Western Blot and Immunofluorescence Microscopy.

| Protein | Species | Dilution | Company |
|---|---|---|---|
| Collagen type 1 | Polyclonal - Goat | 1:1000 (WB), 1:200 (IF) | # 1310-01, Southern Biotech. |
| α-SMA | Monoclonal - Mouse | 1:1000 (WB), 1:200 (IF) | # A5228, Sigma Aldrich. |
| α tubulin | Monoclonal - Mouse | 1:1000 (WB) | # T9026, Sigma Aldrich. |
| BCAT 1 | Polyclonal - Rabbit | 1:1000 (WB) | # PA5-120441, Thermo Fisher Scientific. |
| BCAT 2 | Polyclonal - Rabbit | 1:1000 (WB) | # ab95976, abcam. |
| BCKDH | Monoclonal - Rabbit | 1:1000 (WB) | # A700-060, Thermo Fisher Scientific. |

*Abbreviations: α-SMA: α smooth muscle actin; BCAT: branched chain amino-acid transaminase; BCKDH: Branched-chain ketoacid dehydrogenase; IF: Immunofluorescence Microscopy; WB: Western Blot.*

**RNA isolation, cDNA synthesis and RT-PCR**

Following the manufacturer's instructions, RNA was isolated from cells using TRI-reagent (T9424, Sigma-Aldrich), then resuspended in 30 µL of RNase-free water. The concentration and purity of the RNA were quantified using a NanoDrop 2000 Spectrophotometer (ND-2000, Thermo Scientific), and the samples were stored at – 80 °C for further analysis. Reverse transcription was performed using random primers (48190011, Thermo Fisher Scientific), RNase-OUT (10777019, Thermo Fisher Scientific) and M-MLV reverse transcriptase (28-025-

013, Invitrogen, Carlsbad, CA, USA). Subsequently, real-time quantitative PCR (RT-PCR) was performed using the qPCR core kit master mix (Eurogentec, The Netherlands) on a QuantStudio ™ 3 (Thermo Fisher Scientific). The gene expression was normalized to *36b4* for rHSCs and *18S* for hHSCs as housekeeping genes and analyzed using the delta-delta CT method. Primers are listed in Table 2.

**Table 2.** Sequences of primers for RT-PCR in human and rat.

| Gene | Forward 5' - 3' | Reverse 3'- 5' | Probe |
|---|---|---|---|
| Rat | | | |
| *Col1α1* | TGGTGAACGTGGTG TACAAGGT | CAGTATCACCCTTGG CACCAT | TCCTGCTGGTCCCCGA GGAAACA |
| *Acta2* | GCCAGTCGCCATCA GGAAC | CACACCAGAGCTGT GCTGTCTT | CTTCACACATAGCTGG AGCAGCTTCTCGA |
| *36b4* | GCTTCATTGTGGGA GCAGACA | CATGGTGTTCTTGCC CATCAG | TCCAAGCAGATGCAG CAGATCCGC |
| *Bcat 1* | GAAGAACGCATTGC AGAAGCT | GGCCGGACCTCAAC ATGA | TCAAACTCCGGCAGA GTGGTCCTCA |
| *Bcat 2* | CCTATTCCCTGGCG TCTATGTG | TGGCTCTCTGGTCAC CTGAAC | CTCCAACTTCAAGGC GGCAGACCTC |
| *Bckdh* | CACGATCTGATTGG CATTGG | AGCGTCACTTCGTCA CCATTT | CCTACCGCCTGAGGG ATCTGCGT |
| Human | | | |
| *COL1A 1* | GGCCCAGAAGAAC TGGTACATC | CCGCCATACTCGAAC TGGAA | CCCCAAGGACAAGAG GCATGTCTG |
| *ACTA2* | GGGACGACATGGA AAAGATCTG | CAGGGTGGGATGCT CTTCA | CACTCTTTCTACAATG AGCTTCGTGTTGCCC |
| *18S* | CGGCTACCACATCC AAGGA | CCAATTACAGGGCCT CGAAA | CGCGCAAATTACCCA CTCCCGA |

*Abbreviations: 36b4 or Rplp0: ribosomal protein lateral stalk subunit P0; ACTA2: alpha-actin-2; COL1A1: Collagen, type 1, alpha 1; BCAT: branched chain amino-acid transaminase; BCKDH: Branched chain ketoacid dehydrogenase.*

**Immunofluorescence microscopy**

For immunofluorescence staining, cells were seeded in 12-well plates with 18 mm glass coverslips. After treatment, cells were fixed with 4 % formaldehyde (10 minutes) and permeabilized with 0.1 % Triton ™ X - 100 (9036-19-5, Merck) for 30 minutes prior to non-specific blocking with 1 % BSA / PBS (30 minutes). Then, cells were incubated for 1 hour at room temperature with the primary antibodies described in Table 1. Secondary antibodies included Alexa Fluor ™ 488 donkey anti-goat (A-11055, Invitrogen) and Alexa Fluor 568 ™ goat anti-mouse (A-11004, Invitrogen). The DAPI dilution was 1: 1000. Afterwards, the coverslips were mounted on slides using Dako Fluorescence Mounting Medium (S3023, Agilent Technologies, Santa Clara, CA, USA) and stored at 4 °C, covered from light until further use. Images were captured using a Leica Fluorescence microscope (DMI6000, Leica Microsystems, Amsterdam, The Netherlands).

**Cell toxicity determination**

Cell death was detected using SYTOX ™ Green nucleic acid staining (S7020, Thermo Fisher Scientific) following the manufacturer's protocol. Briefly, SYTOX ™ Green was diluted 1 : 40000 in IMDM medium and added for 15 minutes at 37 °C to the cells. Fluorescence was detected using a Leica Fluorescence microscope (DMI6000, Leica Microsystems) with an excitation wavelength of 450 − 490 nm. Hydrogen peroxide 30 % (1.07209.0250, Merck) was used as a positive control at a concentration of 5 mM.

**Cell proliferation assay**

HSCs were seeded in 16-well E-plates. xCELLigence Real-Time Cell Analysis (Agilent Technologies) was performed for rHSCs and hHSCs from cirrhotic tissue, starting on the day

of isolation and continuing for at least 100 hours. Cell index was determined using the RTCA Software (Agilent Technologies).

## REFERENCES


1. Rombouts K, Carloni V (2016) Determination and Characterization of Tetraspanin-Associated Phosphoinositide-4 Kinases in Primary and Neoplastic Liver Cells. Methods Mol Biol 1376:203–212. https://doi.org/10.1007/978-1-4939-3170-5_17

2. Moshage H, Casini A, Lieber CS (1990) Acetaldehyde selectively stimulates collagen production in cultured rat liver fat-storing cells but not in hepatocytes. Hepatology 12:511–518. https://doi.org/10.1002/HEP.1840120311

3. Mederacke I, Dapito DH, Affò S, Uchinami H, Schwabe RF (2015) High-yield and high-purity isolation of hepatic stellate cells from normal and fibrotic mouse livers. Nature Protocols 2014 10:2 10:305–315. https://doi.org/10.1038/nprot.2015.017

4. Pasut A, Jones AE, Rudnicki MA (2013) Isolation and Culture of Individual Myofibers and their Satellite Cells from Adult Skeletal Muscle. J Vis Exp 50074. https://doi.org/10.3791/50074

5. Bomer N, Pavez-giani MG, Deiman FE, Linders AN, Hoes MF, Baierl CLJ, Oberdorf-maass SU, de Boer RA, Silljé HHW, Berezikov E, Simonides WS, Westenbrink BD, van der Meer P (2021) Selenoprotein DIO2 Is a Regulator of Mitochondrial Function, Morphology and UPRmt in Human Cardiomyocytes. Int J Mol Sci 22:. https://doi.org/10.3390/IJMS222111906

6. Hoes MF, Grote Beverborg N, Kijlstra JD, Kuipers J, Swinkels DW, Giepmans BNG, Rodenburg RJ, van Veldhuisen DJ, de Boer RA, van der Meer P (2018) Iron deficiency impairs contractility of human cardiomyocytes through decreased mitochondrial function. Eur J Heart Fail 20:910–919. https://doi.org/10.1002/EJHF.1154

7. Breckwoldt K, Letuffe-Brenière D, Mannhardt I, Schulze T, Ulmer B, Werner T, Benzin A, Klampe B, Reinsch MC, Laufer S, Shibamiya A, Prondzynski M, Mearini G, Schade D, Fuchs S, Neuber C, Krämer E, Saleem U, Schulze ML, Rodriguez ML, Eschenhagen T, Hansen A (2017) Differentiation of cardiomyocytes and generation of human engineered heart tissue. Nat Protoc 12:1177–1197. https://doi.org/10.1038/NPROT.2017.033

8. Burridge PW, Matsa E, Shukla P, Lin ZC, Churko JM, Ebert AD, Lan F, Diecke S, Huber B, Mordwinkin NM, Plews JR, Abilez OJ, Cui B, Gold JD, Wu JC (2014) Chemically defined generation of human cardiomyocytes. Nature Methods 2014 11:8 11:855–860. https://doi.org/10.1038/nmeth.2999

**SUPPLEMENTAL FIGURES**

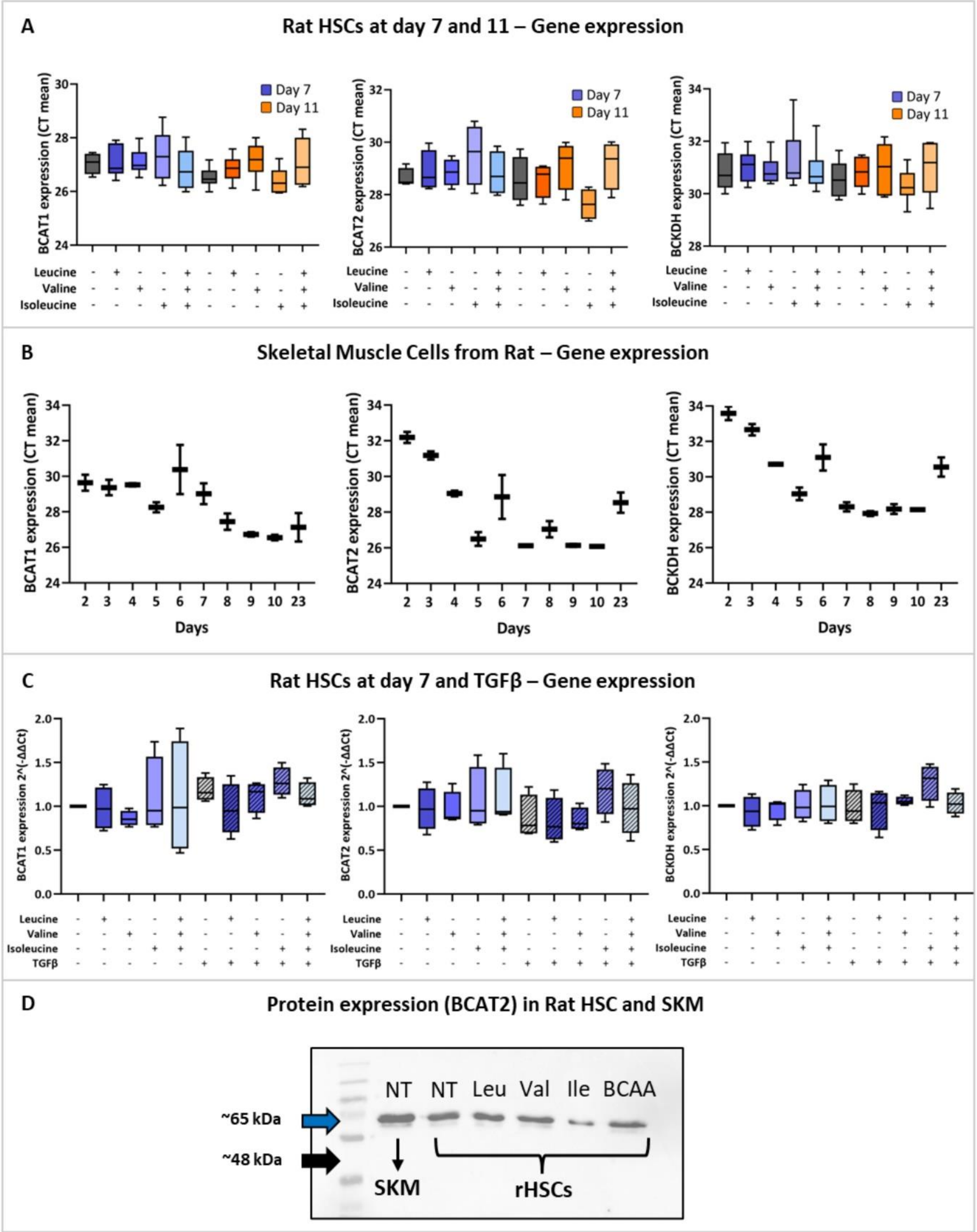


**Supplemental Fig S1. Gene and protein expression of BCAA enzymes in rHSCs.** Panel A illustrates the Ct mean values for *BCAT1*, *BCAT2*, and *BCKDH* gene expression in rHSCs treated from day 1 to 7 (blue bars) or from day 7 to 11 (orange bars) with BCAAs. Panel B illustrates Ct mean values for *BCAT1*, *BCAT2*, and *BCKDH* gene expression in SKM cells from

rat at different time points. Panel C illustrates the the delta-delta CT values for *BCAT1, BCAT2,* and *BCKDH* gene expression in rHSCs treated from day 1 to 7 (blue bars) with BCAAs, and rHSCs treated with TGF-β and BCAAs (blue bars with black lines). Panel D shows a complete membrane from a western blot, displaying the band for BCAT2 at approximately 65 kDa for SKM and rHSCs, instead of at ~48 kDa as suggested by the manufacturer. *Abbreviations: BCAAs: Branched-chain Amino Acids; BCAT: branched-chain amino acid transaminase; BCKDH: branched-chain keto acid dehydrogenase; Ile: Isoleucine; Leu: Leucine; SKM: skeletal muscle cells; Val: Valine.*

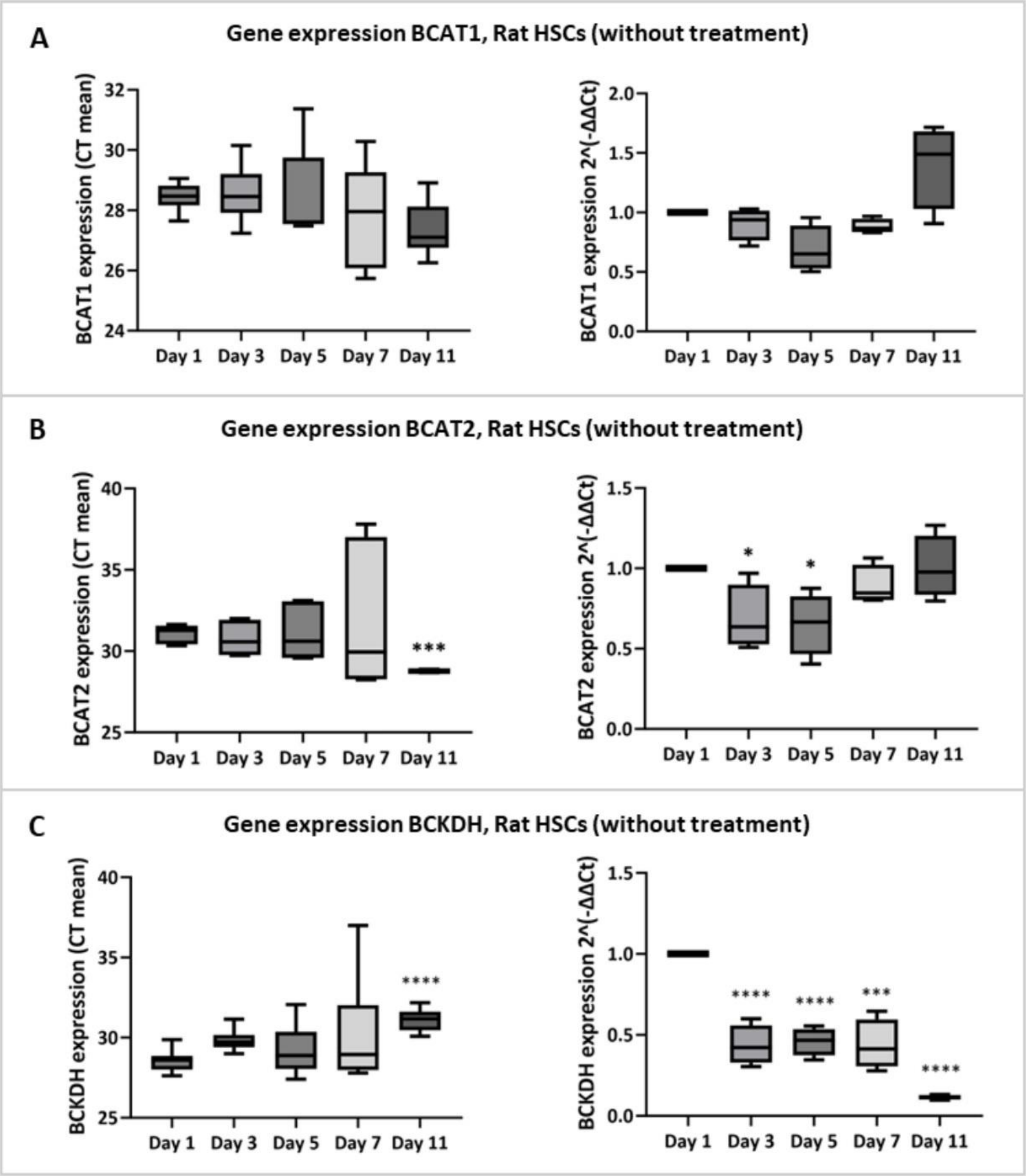


**Supplemental Fig S2. Gene expression of *BCAT1* and *BCKDH* in rHSCs during different culture times.** Panel A shows the gene expression of *BCAT1* as Ct mean (left) and delta-delta CT (right) in rHSCs at days 1, 3, 5, 7, and 11, without any other intervention. Panel B and Panel C illustrate the gene expression of *BCAT2 and BCKDH* in rHSCs under the same conditions. *Abbreviations: BCAT: branched-chain amino acid transaminase; BCKDH: branched-chain keto acid dehydrogenase.* * $p < 0.01$; *** $p < 0.001$; **** $p < 0.0001$.

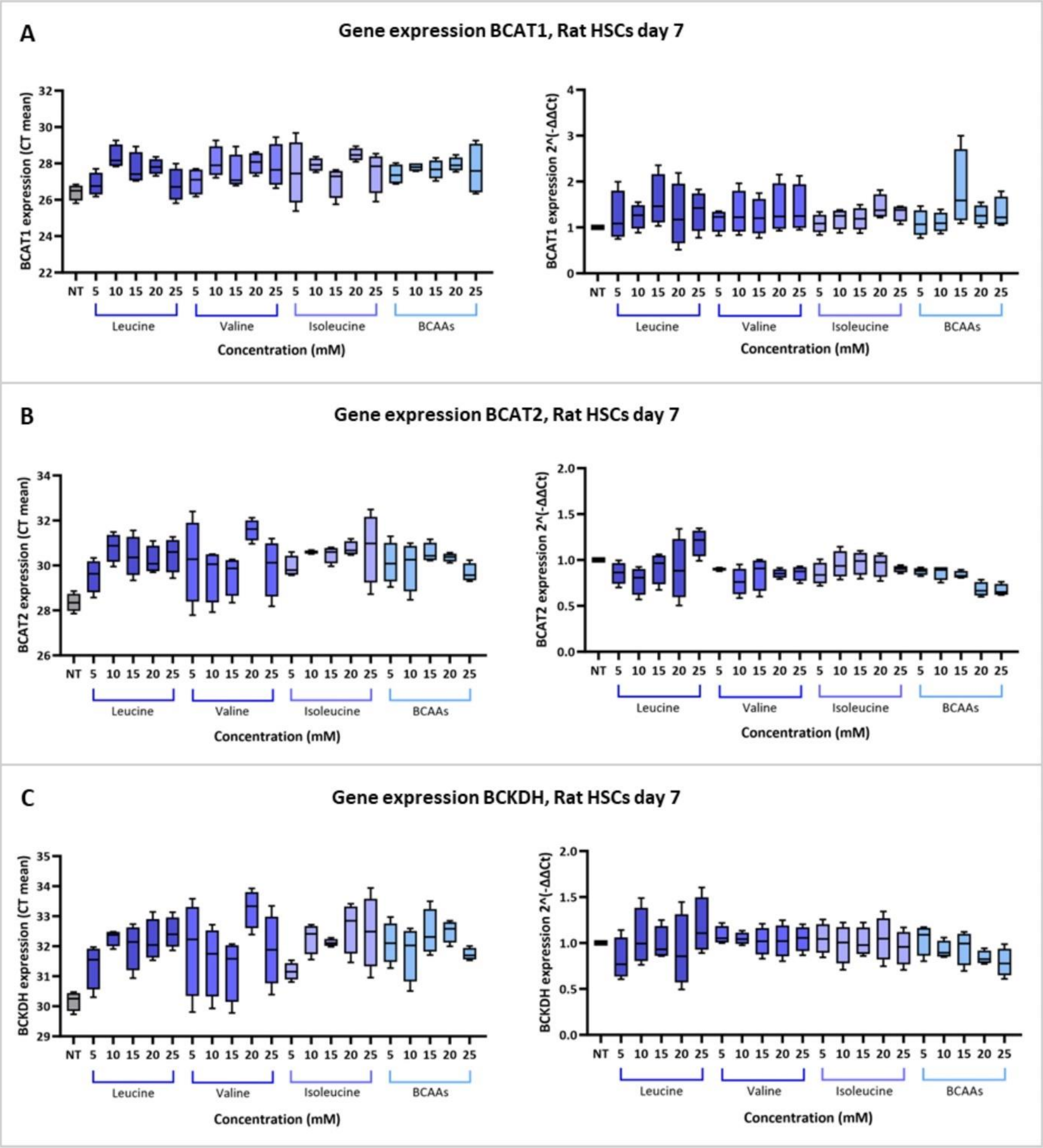


**Supplemental Fig S3. Gene expression of *BCAT* and *BCKDH* in rHSCs at day 7, treated with different concentrations of BCAAs.** The graphs indicate the gene expression of *BCAT1* (Panel A), *BCAT2* (Panel B) and *BCKDH* (Panel C) as Ct mean (left) and delta-delta CT (right) in rHSCs after 6 days of culturing (blue bars) with BCAAs at 5, 10, 15, 20 and 25 mM. *Abbreviations: BCAT: branched-chain amino acid transaminase; BCKDH: branched-chain keto acid dehydrogenase; NT: non-treatment.*

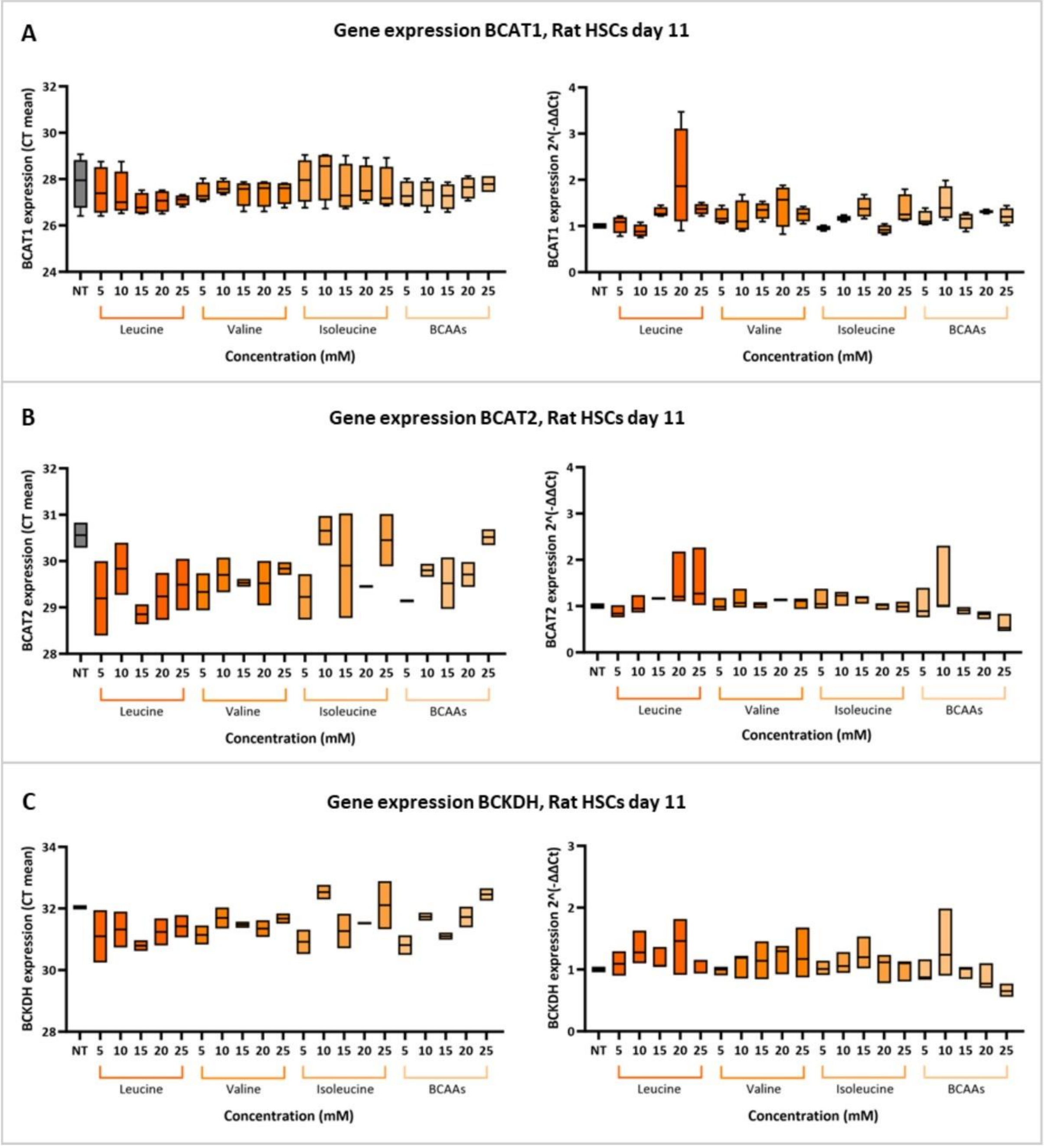


**Supplemental Fig S4. Gene expression of *BCAT* and *BCKDH* in rHSCs at day 11, treated with different concentrations of BCAAs.** The graphs indicate the gene expression of *BCAT1* (Panel A), *BCAT2* (Panel B) and *BCKDH* (Panel C) as Ct mean (left) and delta-delta CT (right) in rHSCs after 4 days of culturing (orange bars) with BCAAs at 5, 10, 15, 20 and 25 mM. *Abbreviations: BCAT: branched-chain amino acid transaminase; BCKDH: branched-chain keto acid dehydrogenase; NT: non-treatment.*

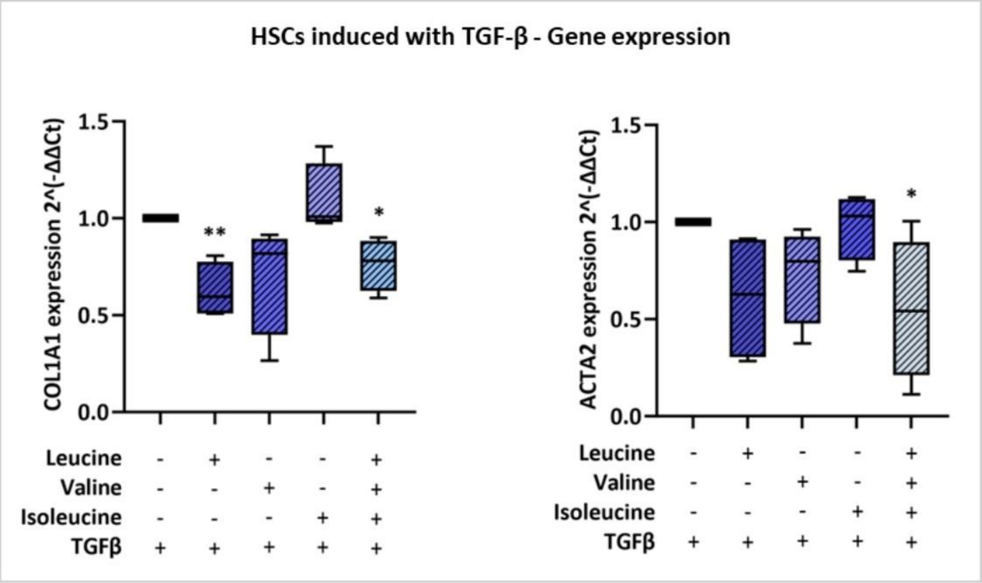


**Supplemental Fig S5. Gene expression of *COL1A1* and *ACTA2* in rHSCs indiced with TGF-β.** The graphs indicate the gene expression of *COL1A1* (left) and *ACTA2* (right) as delta-delta Ct in rHSCs that were first induced with TGF-β and then cultured in BCAAs-enriched medium. * $p < 0.05$; ** $p < 0.01$.

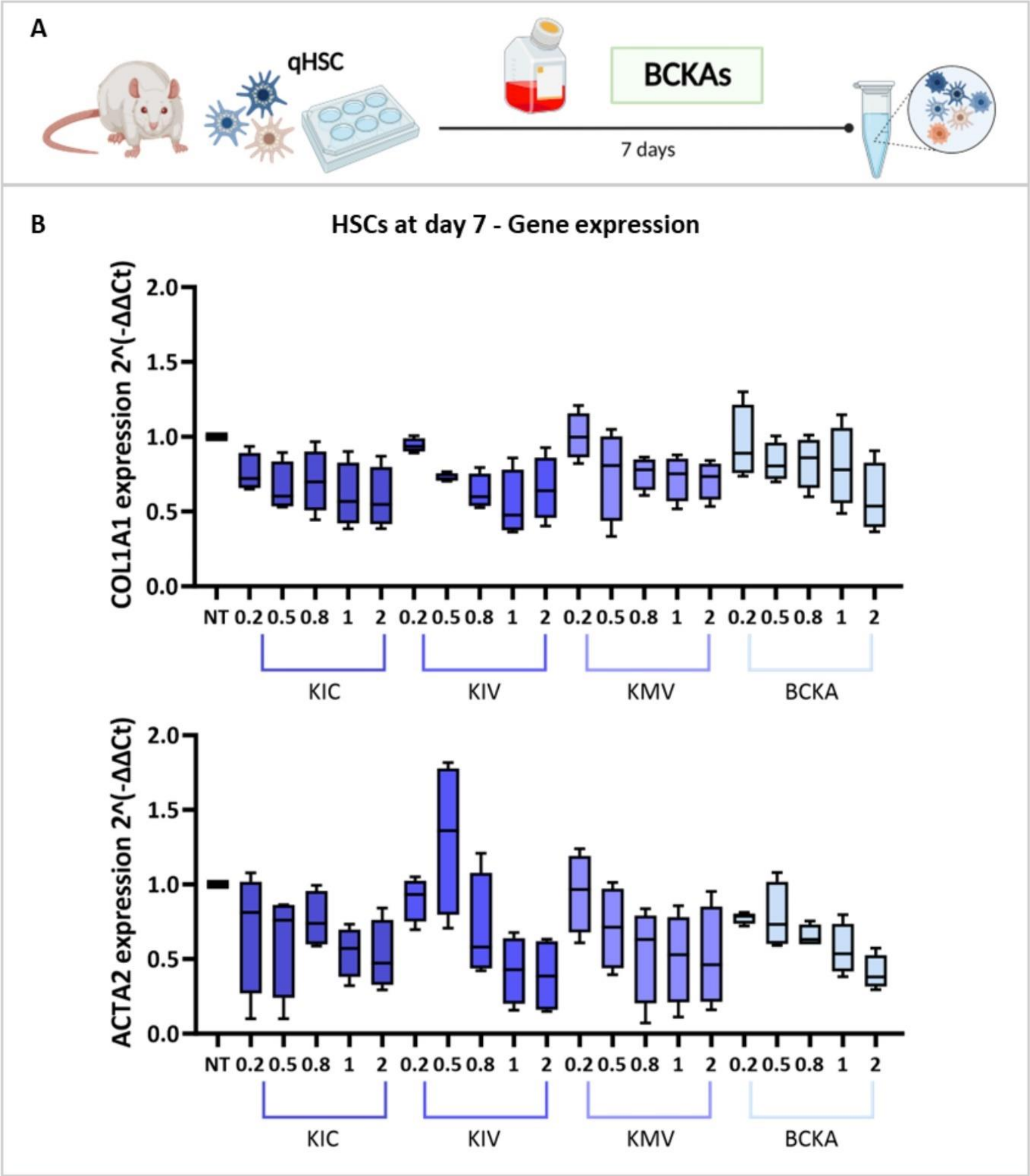


**Supplemental Fig S6. Gene expression of *COL1A1* and *ACTA2* in rHSCs at day 7, treated with different concentrations of BCKAs.** The graphs indicate the gene expression of *COL1A1* (up) and *ACTA2* (down) as delta-delta Ct in rHSCs after 6 days of culturing (blue bars) with BCKAs at 0.2, 0.5, 0.8, 1 and 2 mM. *Abbreviations: BCKAs: Branched-chain Keto Acids; Ile: Isoleucine; KIC: α-Ketoisocaproate; KIV: α-Ketoisovalerate; KMV: α-Keto-β-methylvalerate.*

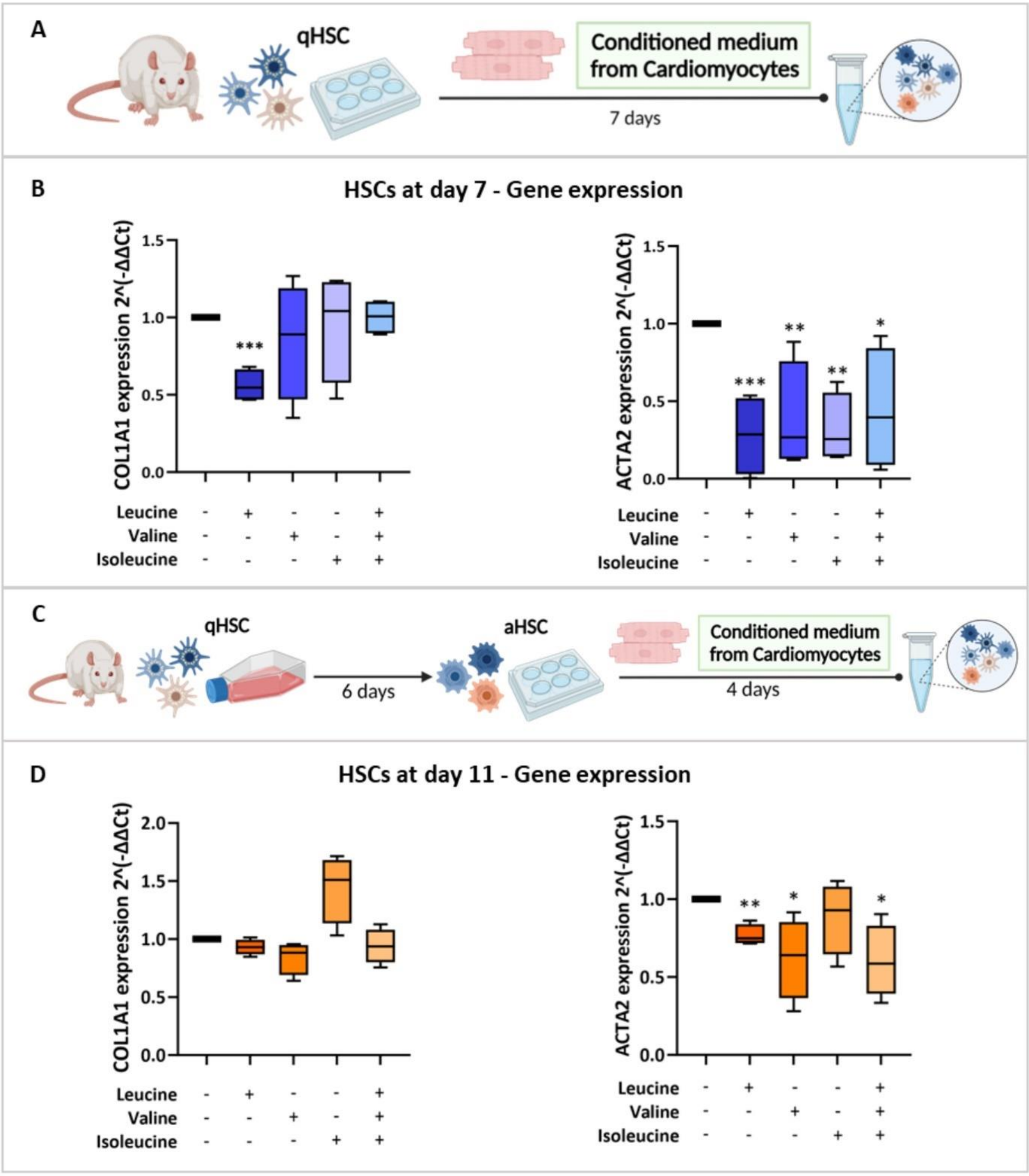


**Supplemental Fig S7. Gene expression of COL1A1 and ACTA2 in rHSCs at day 7 and 11, treated with conditioned medium from cardiomyocytes.** The graphs indicate the gene expression of COL1A1 (left) and ACTA2 (right) as delta-delta Ct in rHSCs at day 7 (blue bars) and 11 (orange bars) that were cultured in conditioned medium from cardiomyocytes, containing the metabolic products of BCAAs 15 mM. * $p < 0.05$; ** $p < 0.01$; *** $p < 0.001$.

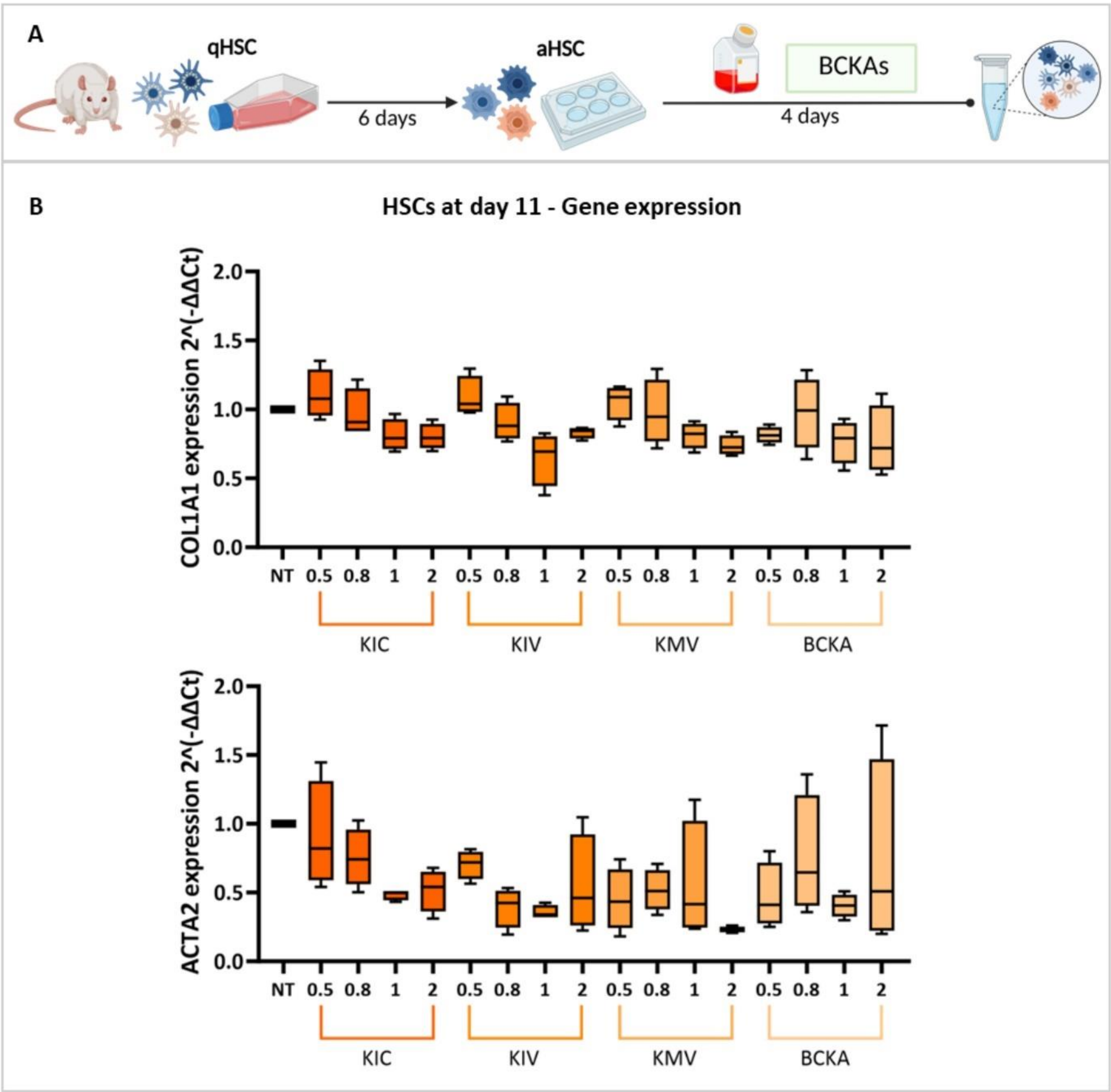


**Supplemental Fig S8. Gene expression of *COL1A1* and *ACTA2* in rHSCs at day 11, treated with different concentrations of BCKAs.** The graphs indicate the gene expression of *COL1A1* (up) and *ACTA2* (down) as delta-delta Ct in activated rHSCs after 4 days of culturing (orange bars) with BCKAs at 0.2, 0.5, 0.8, 1 and 2 mM. *Abbreviations: BCKAs: branched-chain keto acids; Ile: isoleucine; KIC: α-ketoisocaproate; KIV: α-ketoisovalerate; KMV: α-keto-β-methylvalerate.*

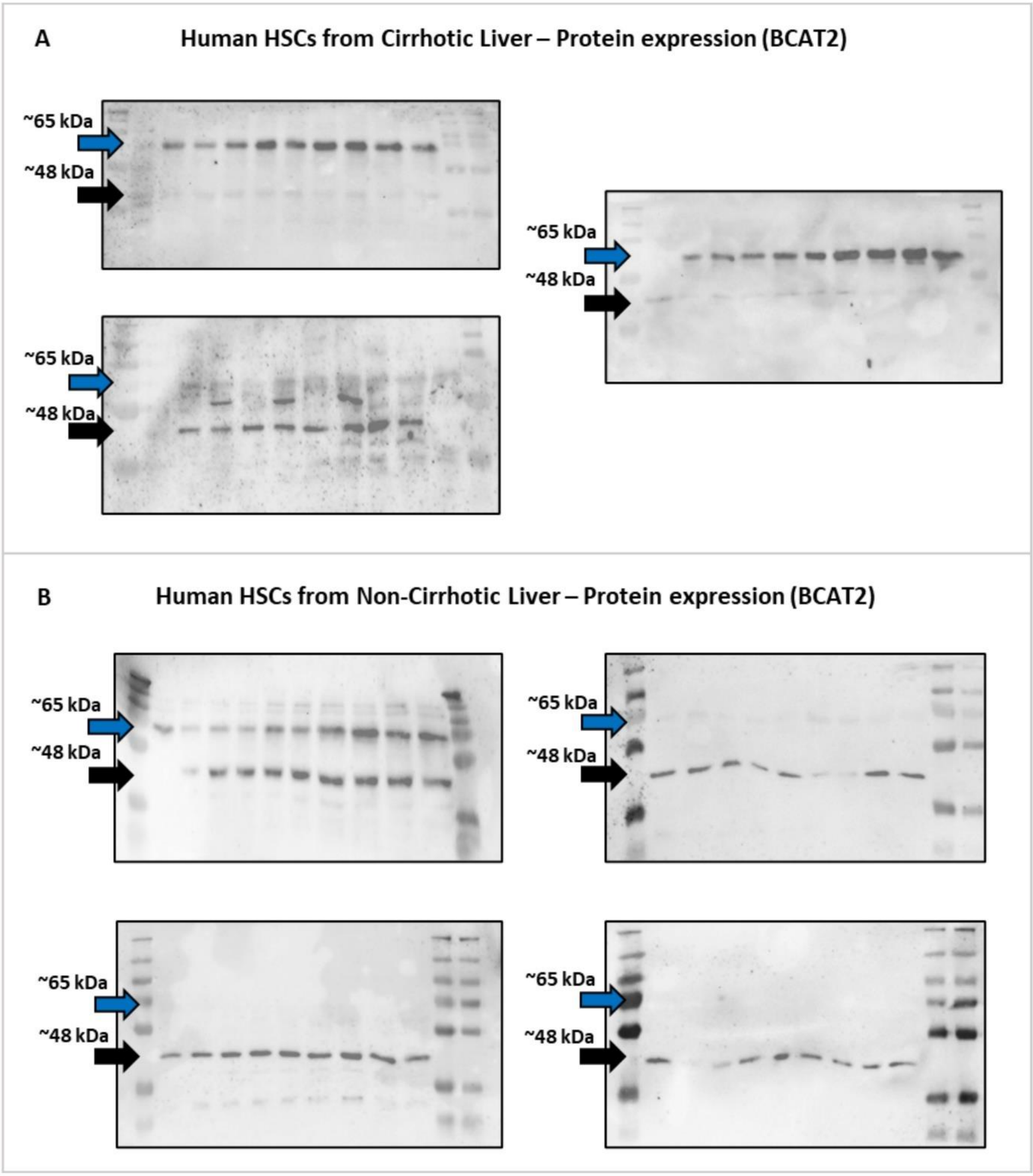


**Supplemental Fig S9. BCAT2 protein expression in hHSCs.** This figure shows the protein bands of BCAT2 in complete western blot membranes from hHSCs of different cirrhotic (Panel A) and non-cirrhotic tissues (Panel B). In two of the three cirrhotic tissue samples, BCAT2 was predominantly expressed at approximately 65 kDa (blue arrows), while in three of the four non-cirrhotic tissue samples, BCAT2 was exclusively expressed at approximately 48 kDa (black arrows). *Abbreviations: BCAT: branched-chain amino acid transaminase.*

**WESTERN BLOT IMAGES**

**Figure 1a:**

BCAT2

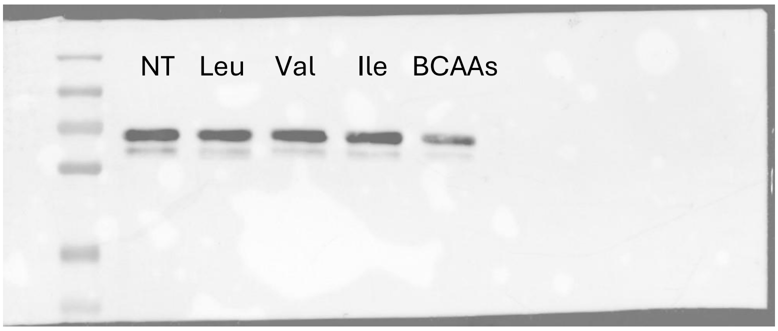


Alpha-tubulin

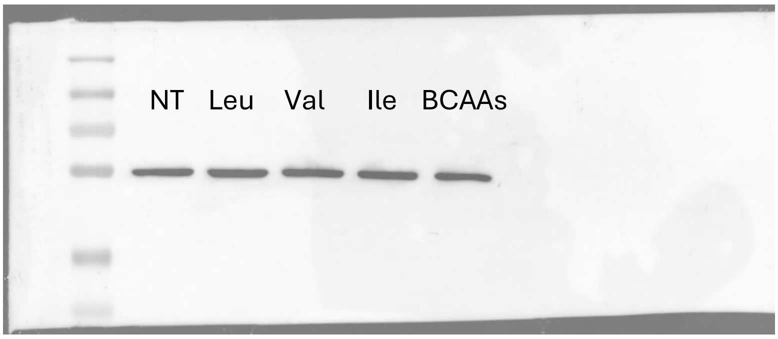


BCAT2

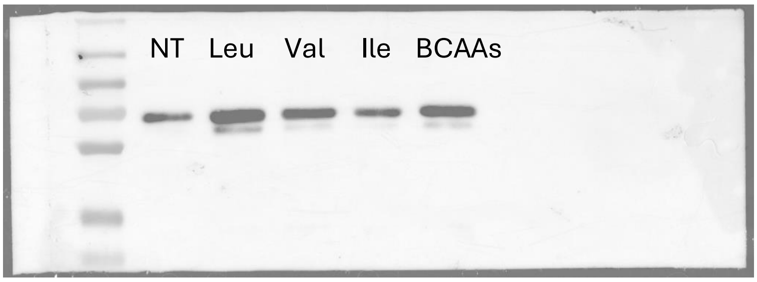


Alpha-tubulin

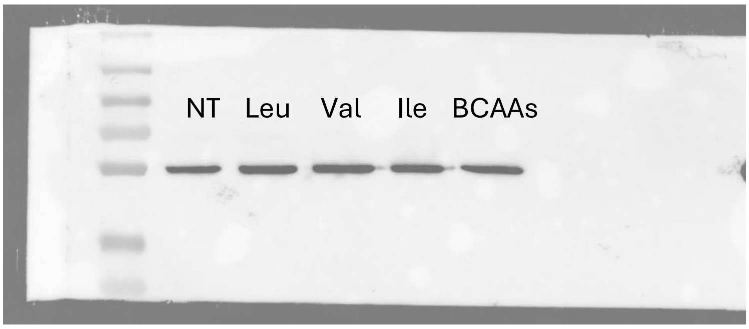


BCAT2

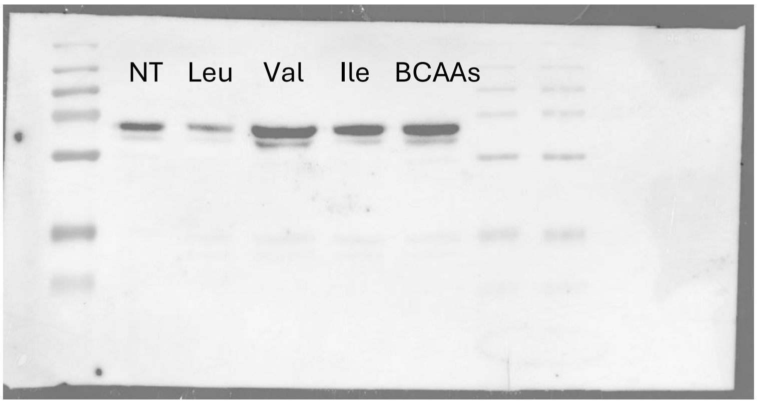


Alpha-tubulin

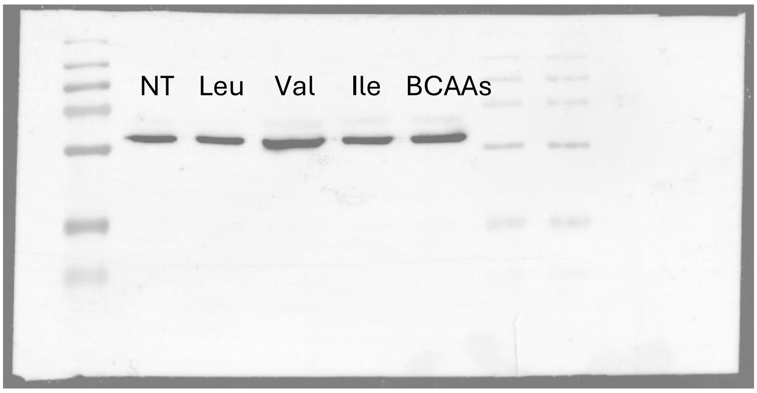


BCKDH

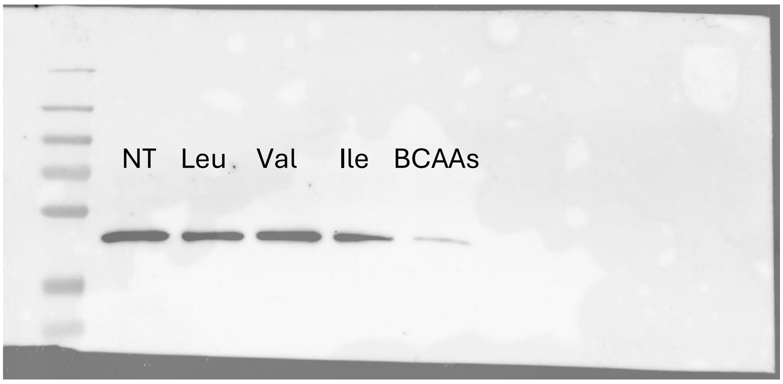


Alpha-tubulin

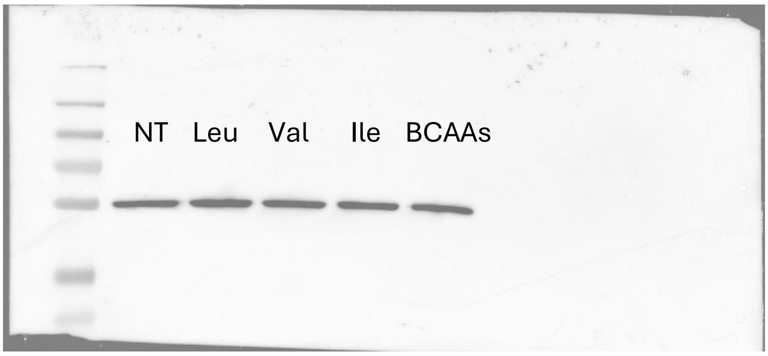


BCKDH

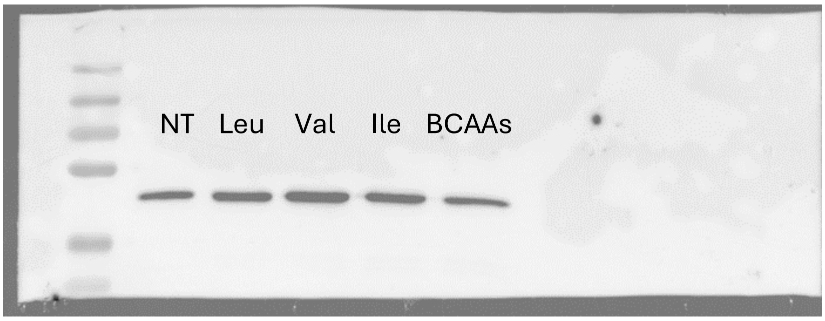


Alpha-tubulin

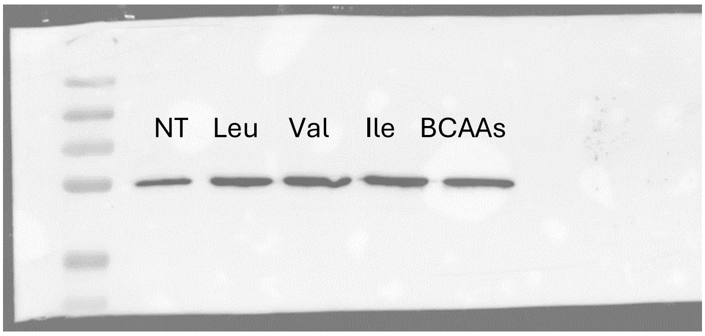


BCKDH

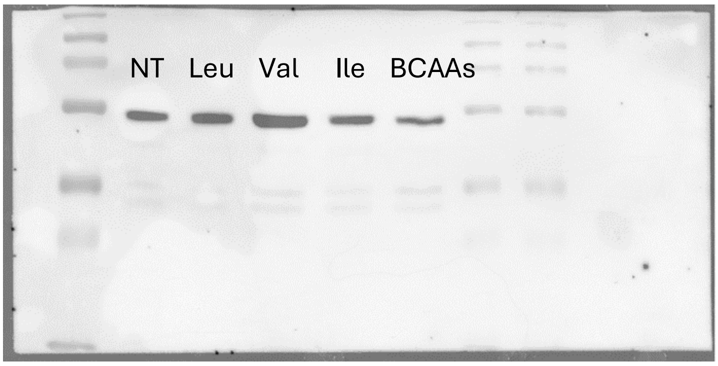


Alpha-tubulin

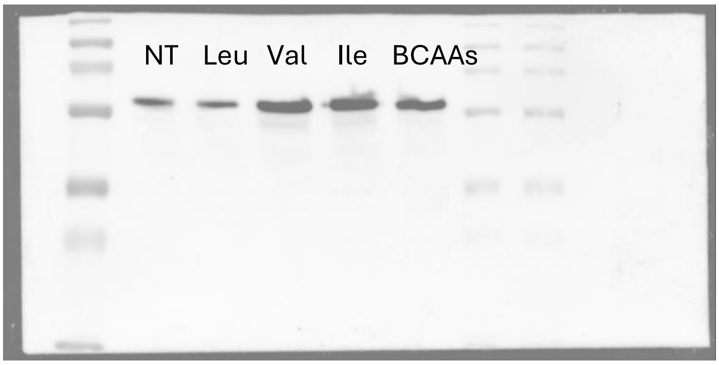


**Figure 1b:**

BCAT2

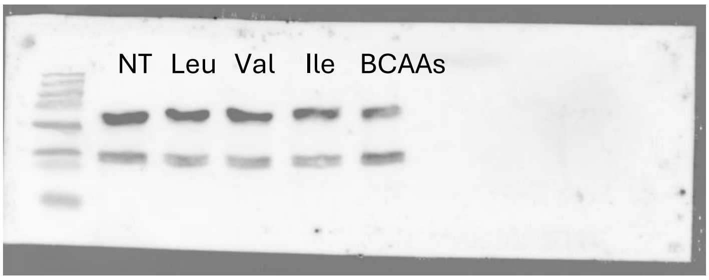


Alpha-tubulin

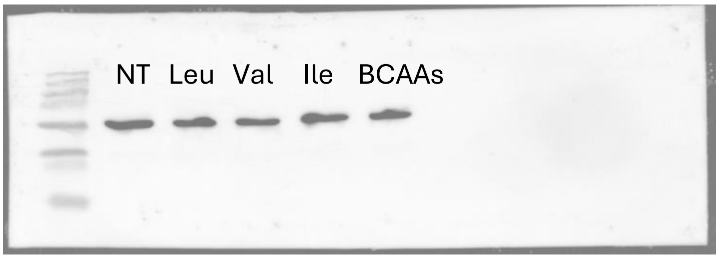


BCAT2

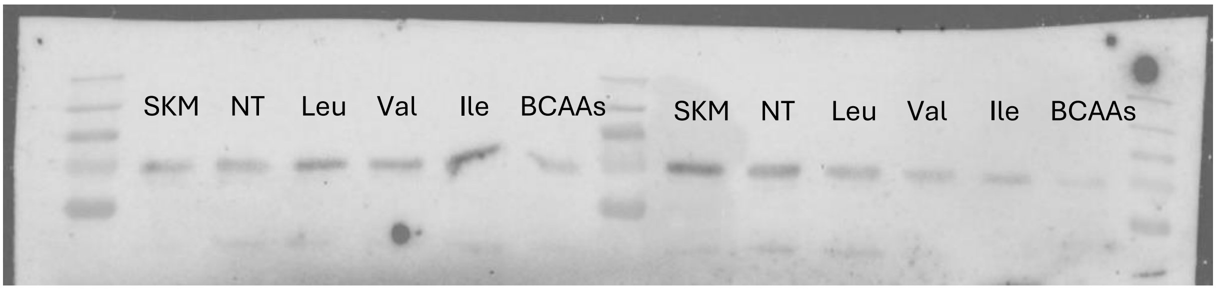


Alpha tubulin

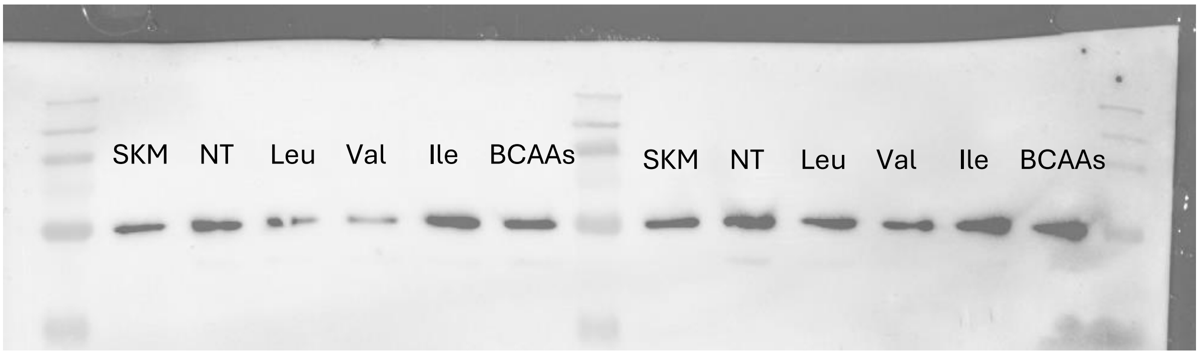


BCKDH

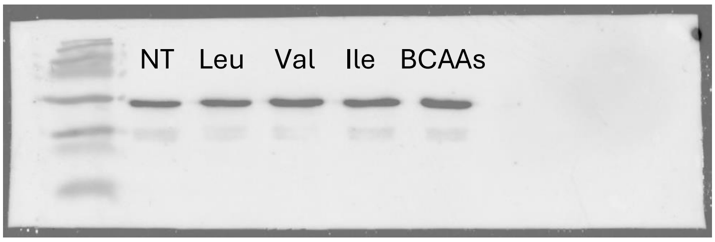


Alpha tubulin

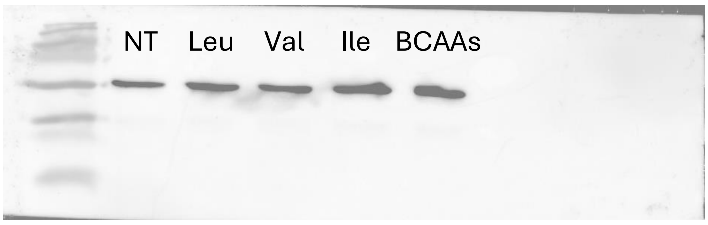


BCKDH

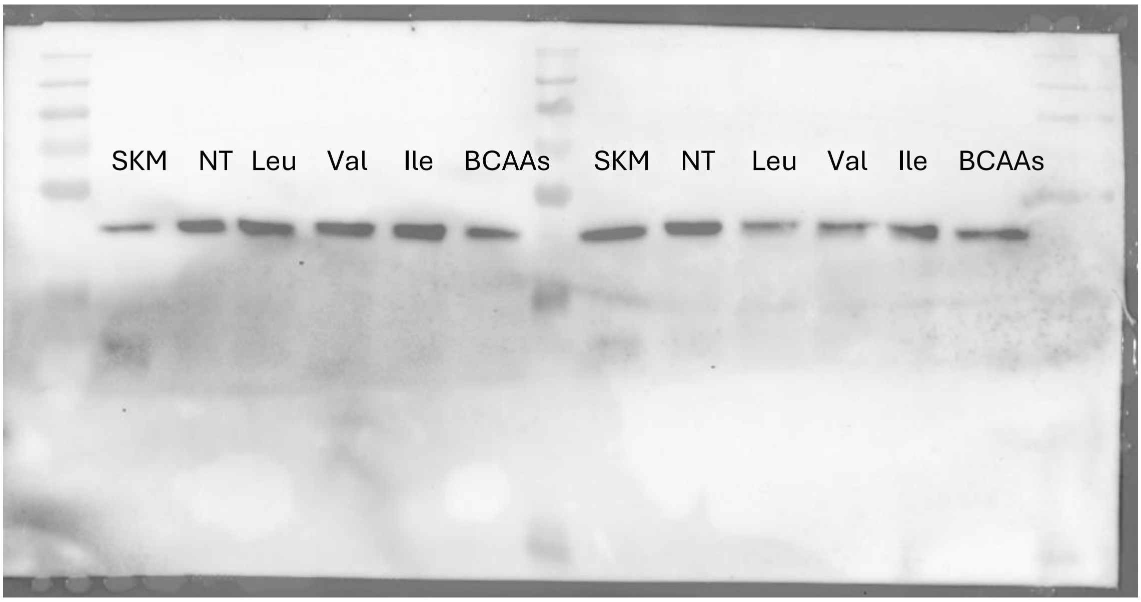


Alpha tubulin

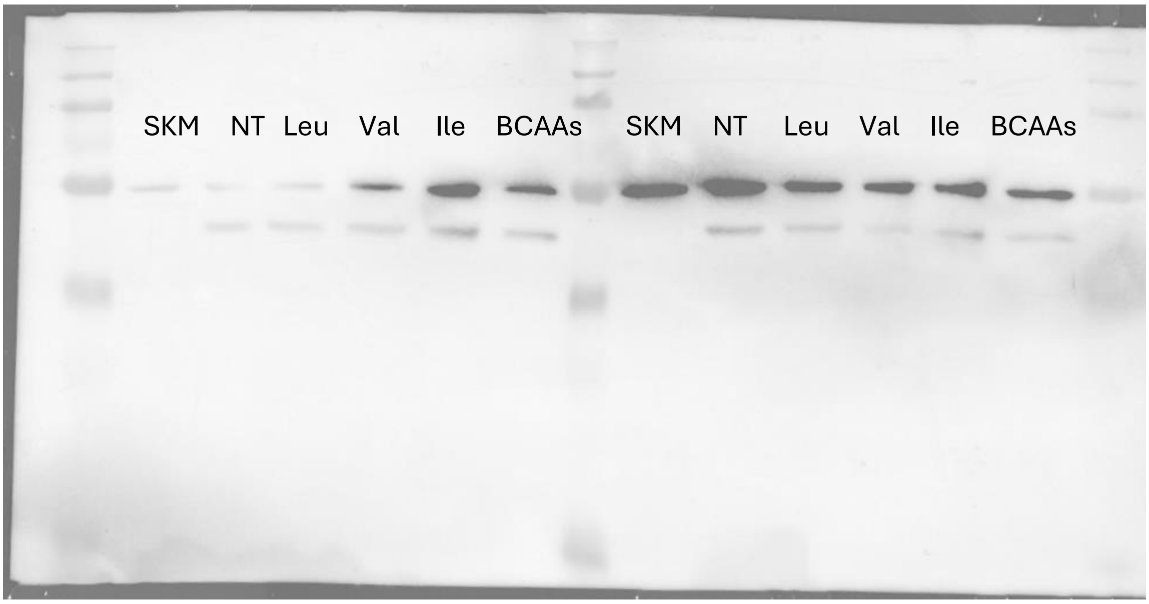


**Figure 2a:**

Collagen type 1

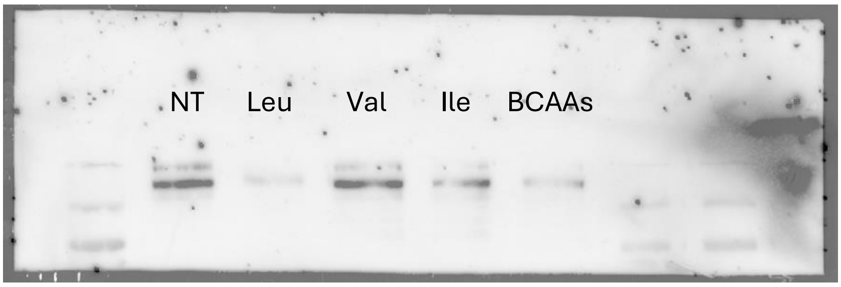


Alpha-tubulin

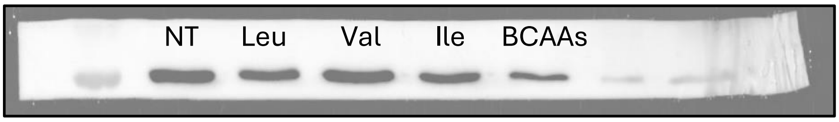


AlphaSMA

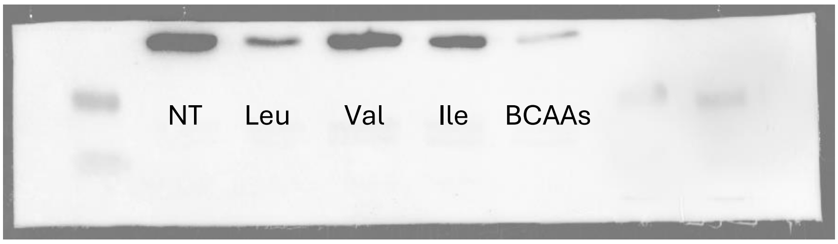


Collagen type 1

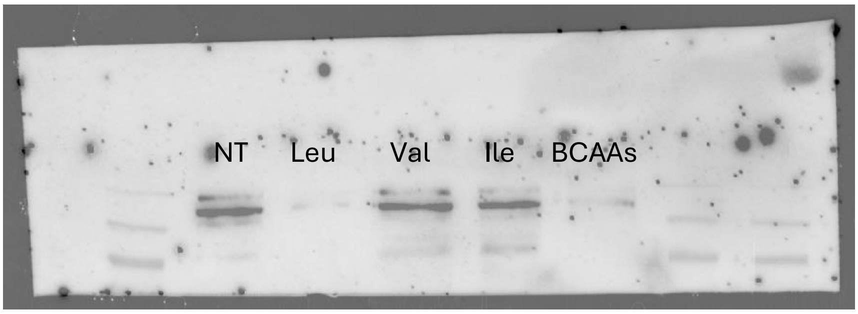


Alpha-tubulin

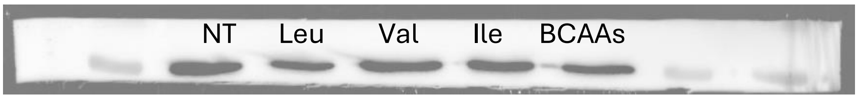


AlphaSMA

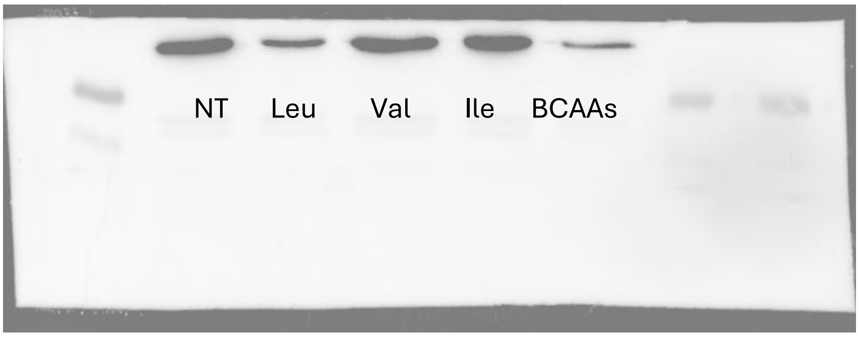


Collagen type 1

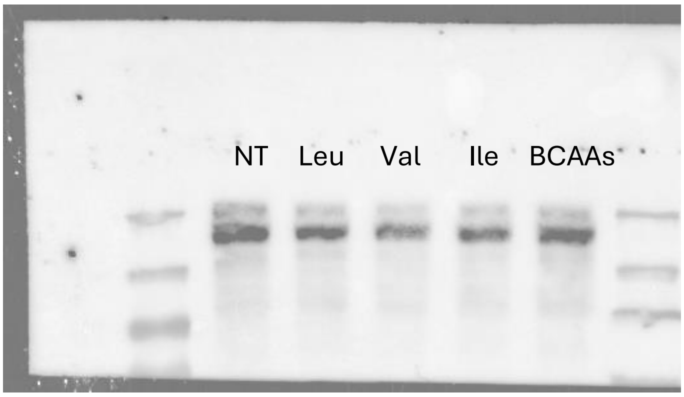


Alpha tubulin

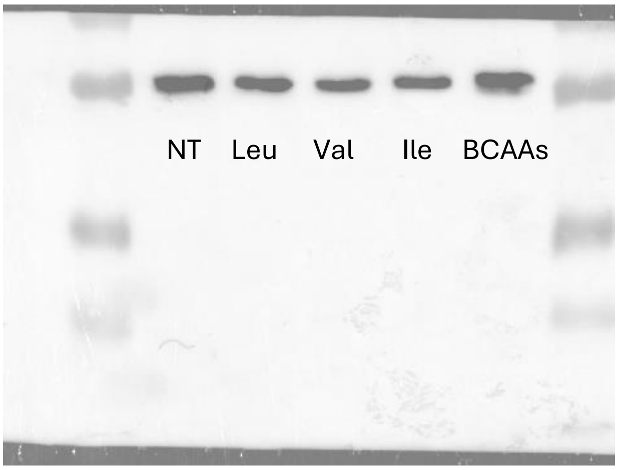


AlphaSMA

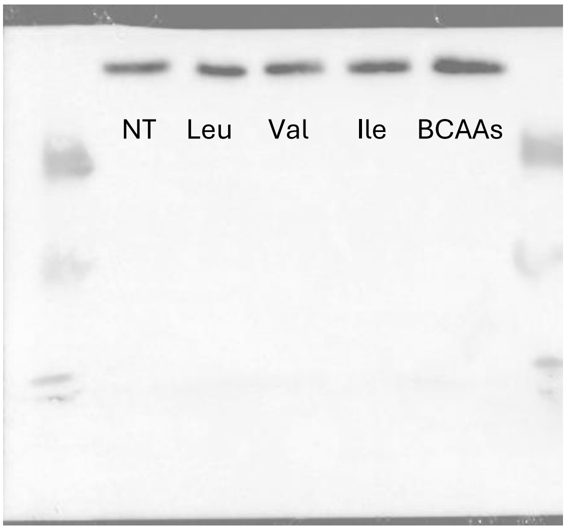


AlphaSMA

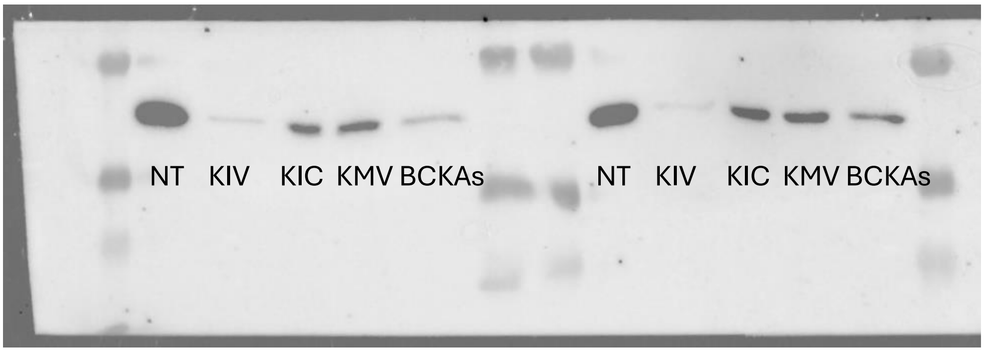


Alpha tubulin

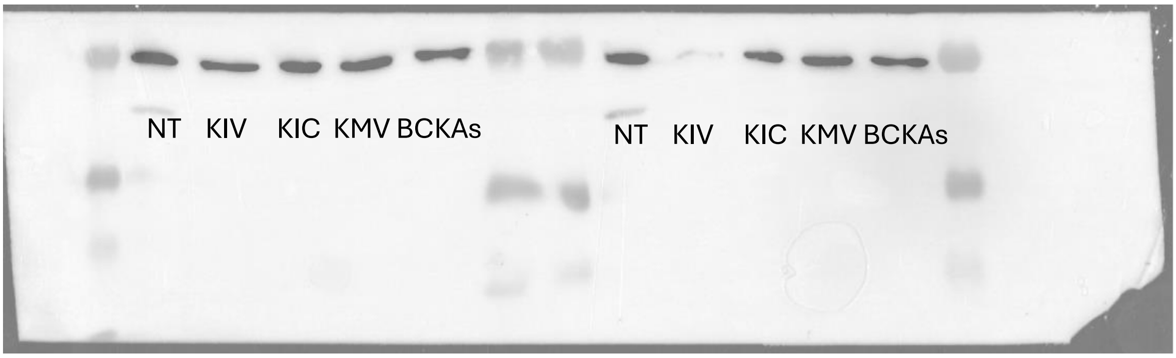


AlphaSMA

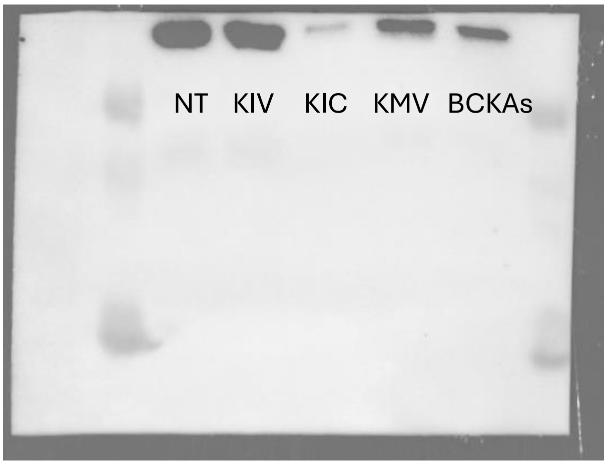


Alpha tubulin

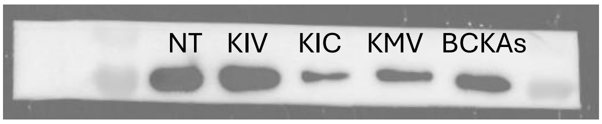

Collagen type 1

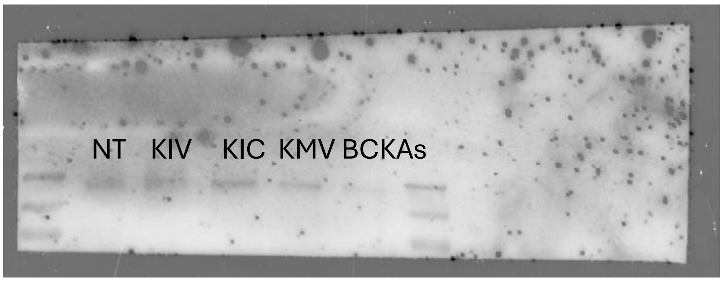


Alpha tubulin

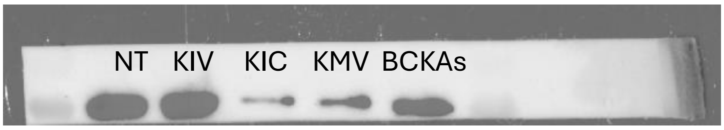


AlphaSMA

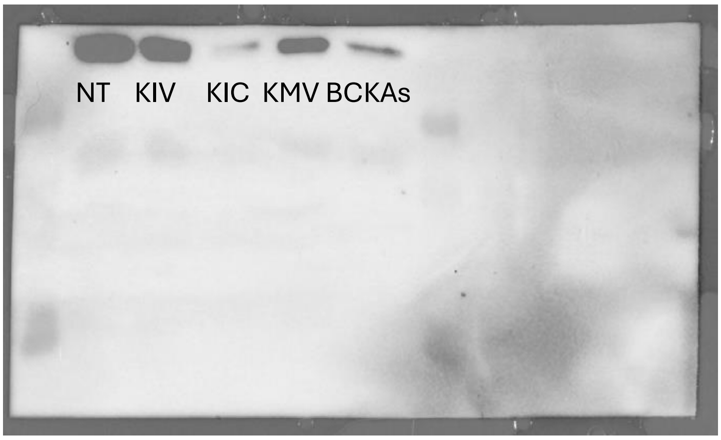


Collagen type 1

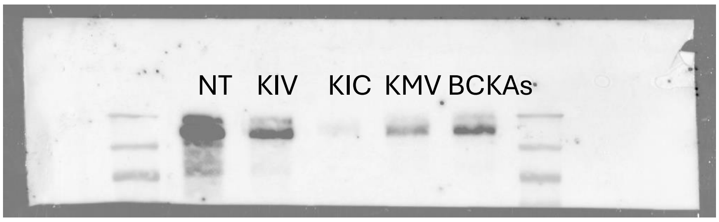


Alpha tubulin

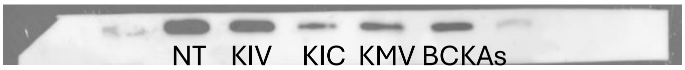


AlphaSMA

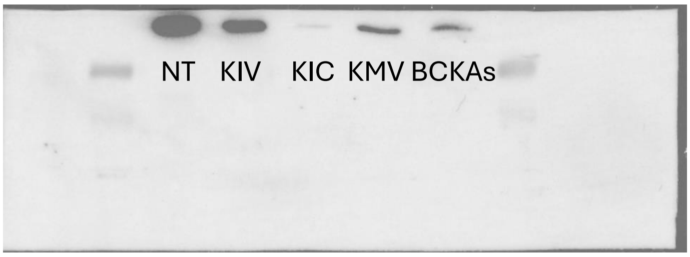


**Figure 3a:**

Collagen type 1

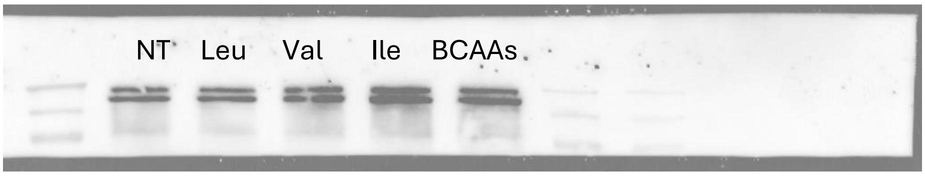


Alpha tubulin

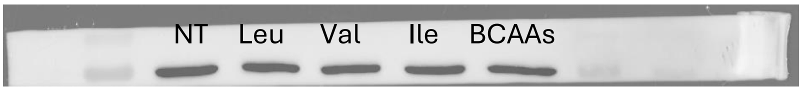


AlphaSMA

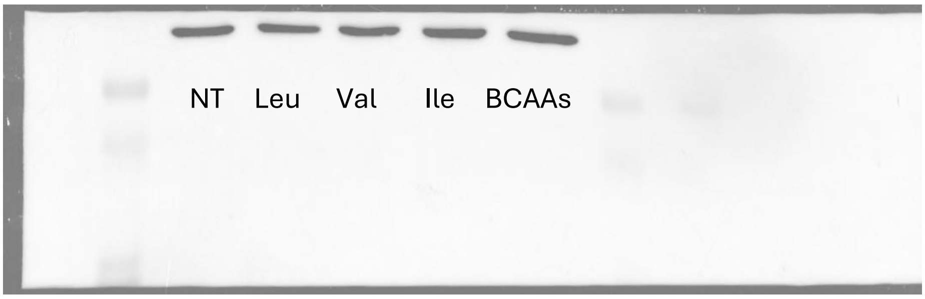


Collagen type 1

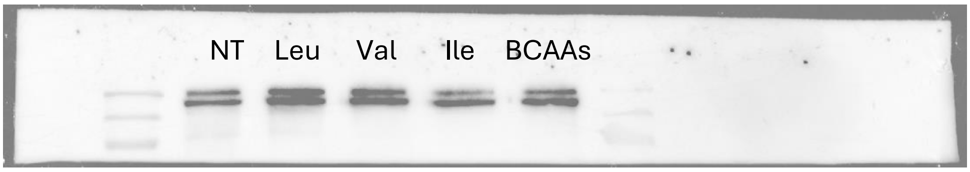


Alpha tubulin

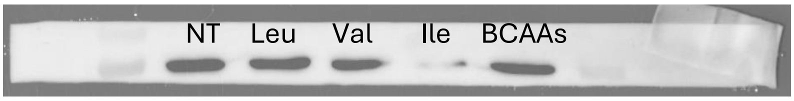


AlphaSMA

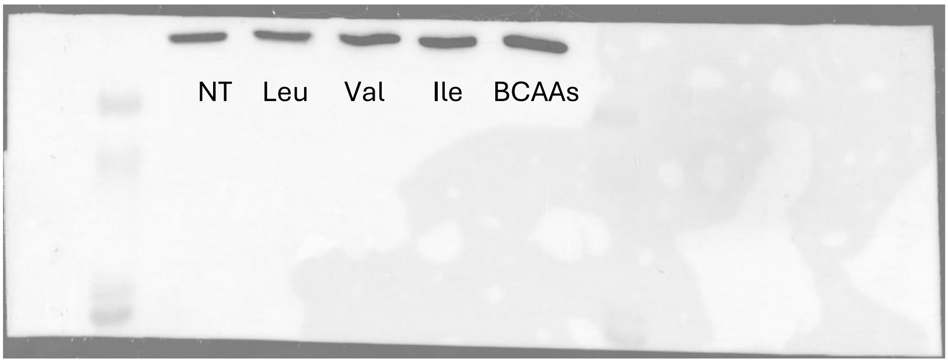


Collagen type 1

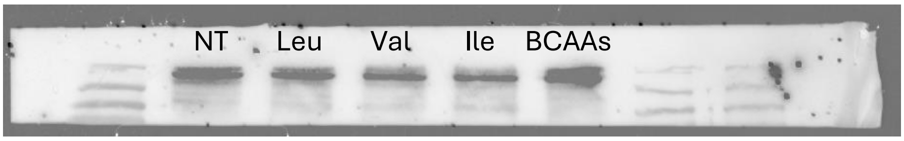


Alpha tubulin

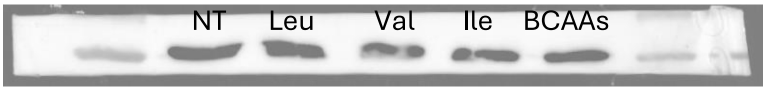


AlphaSMA

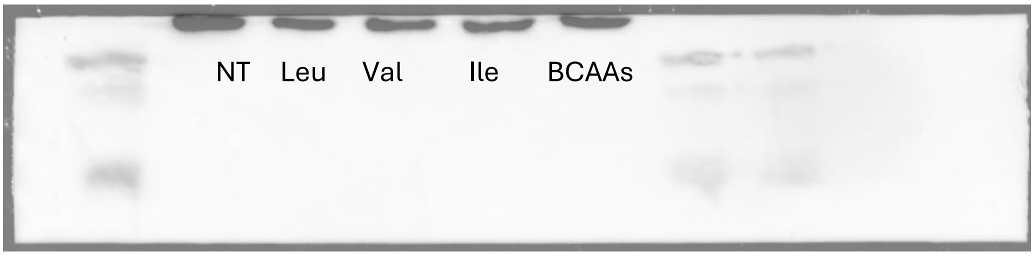


Collagen type 1

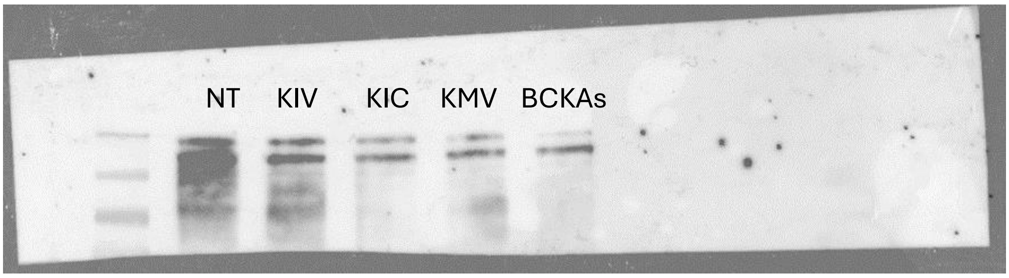


Alpha tubulin

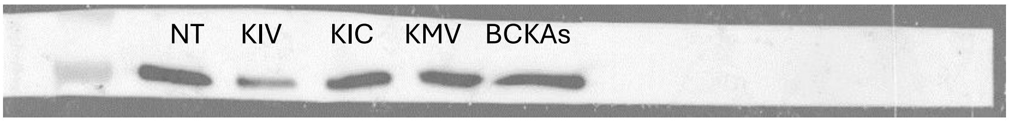


AlphaSMA

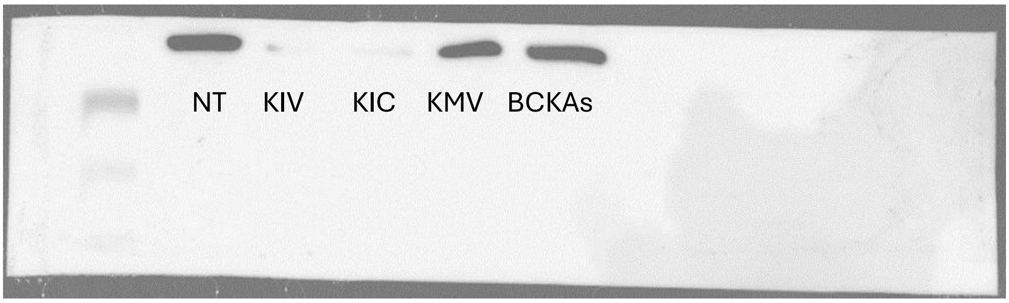


Collagen type 1

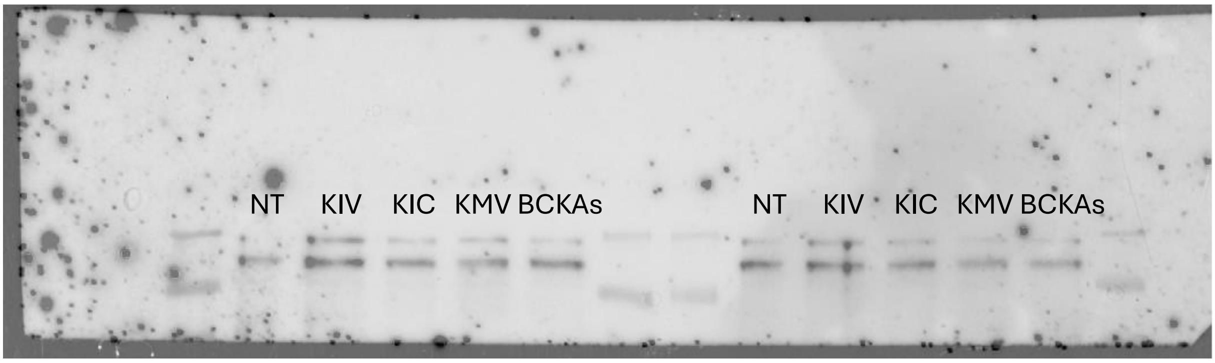


Alpha tubulin

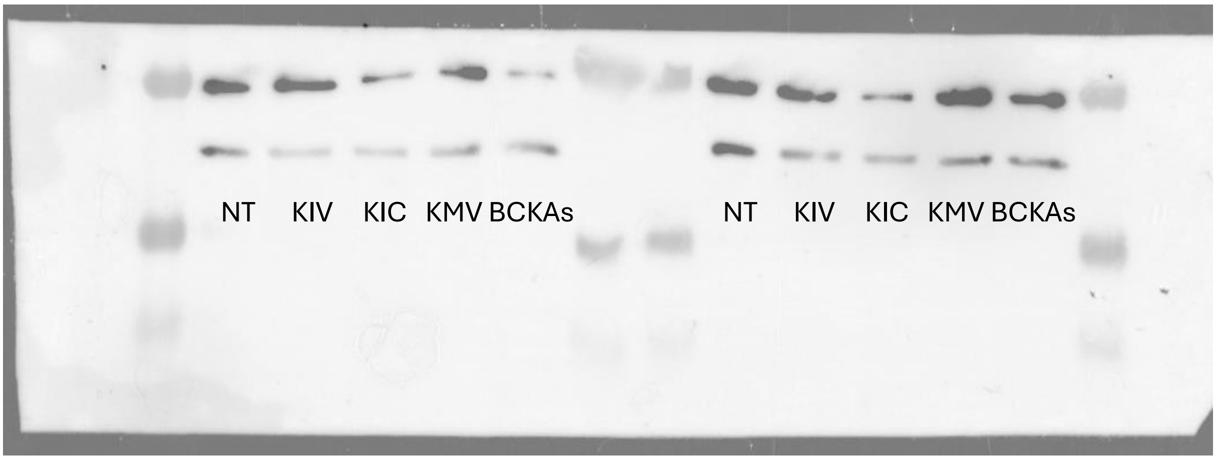


AlphaSMA

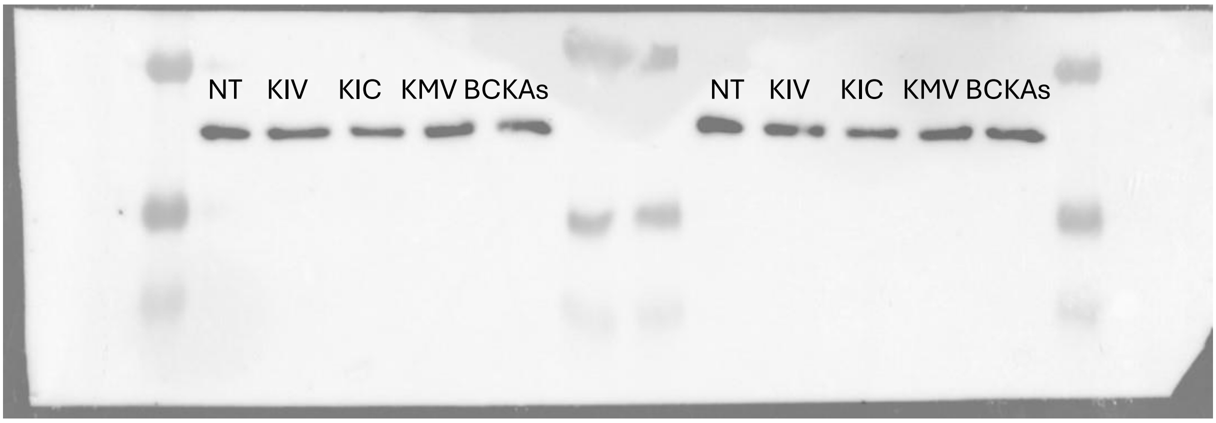


**Figure 4a:**

BCAT1

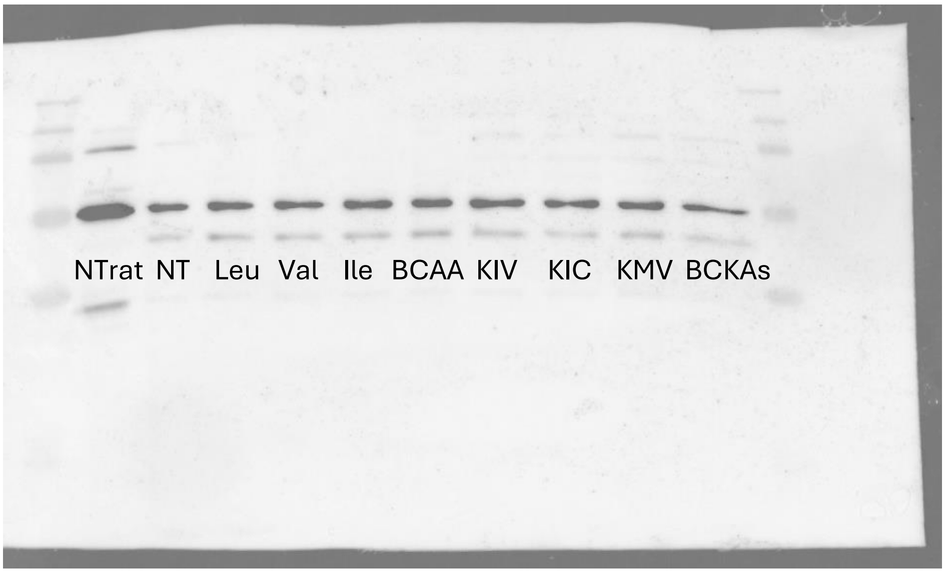


BCAT 2

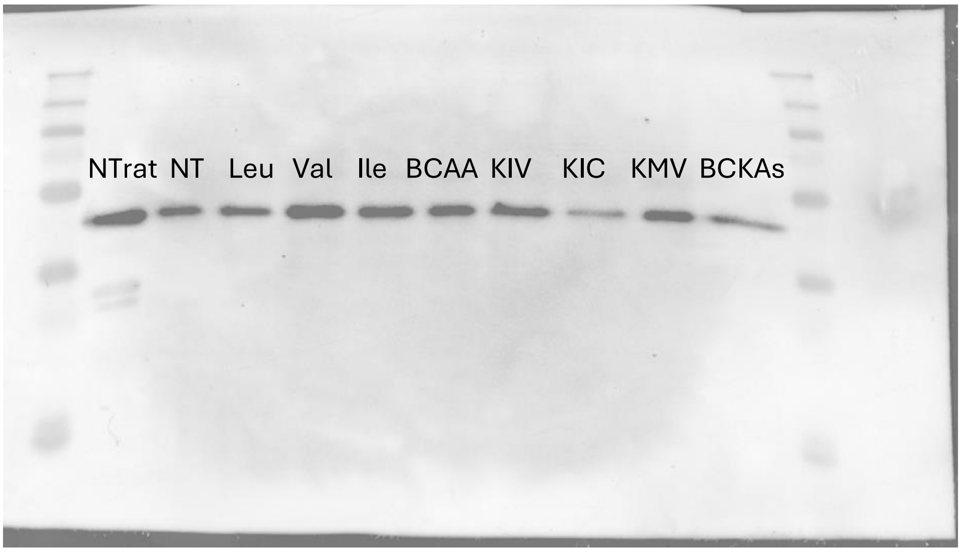


BCKDH

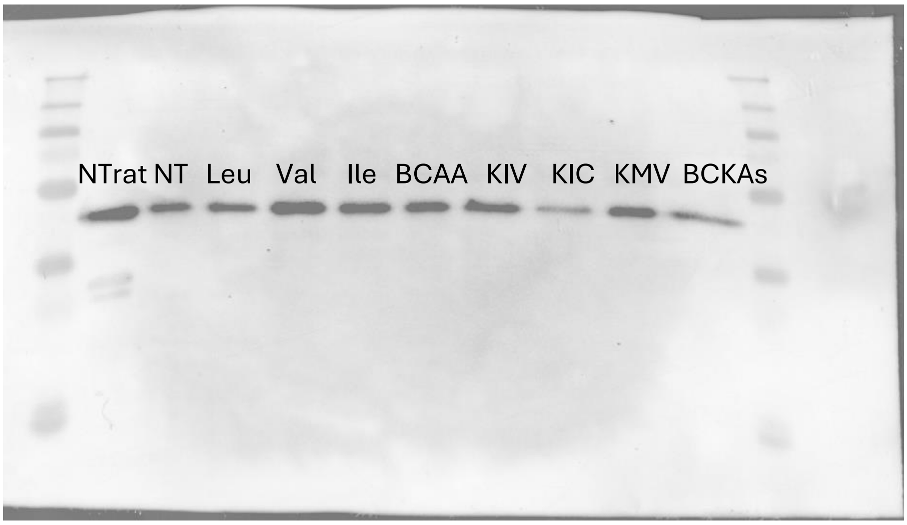


Alpha tubulin

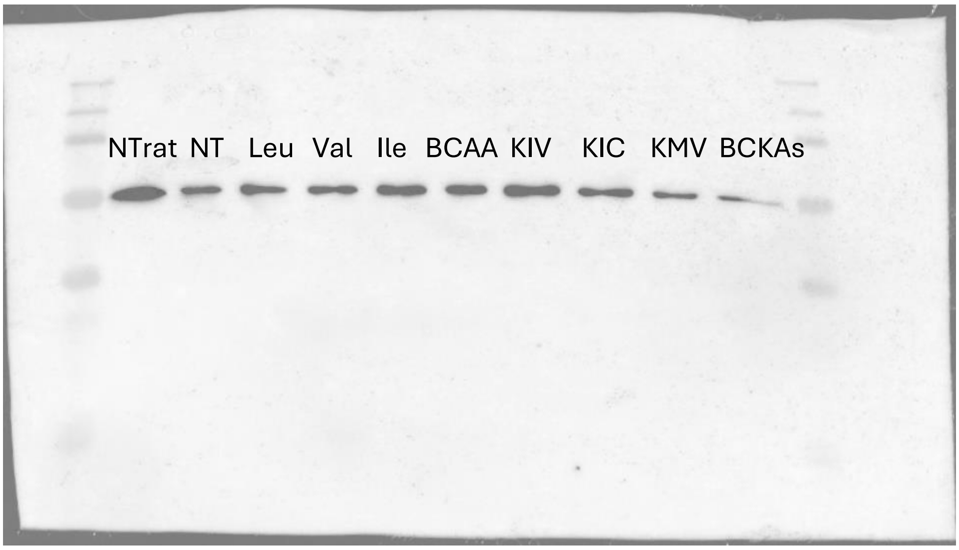


BCAT 2

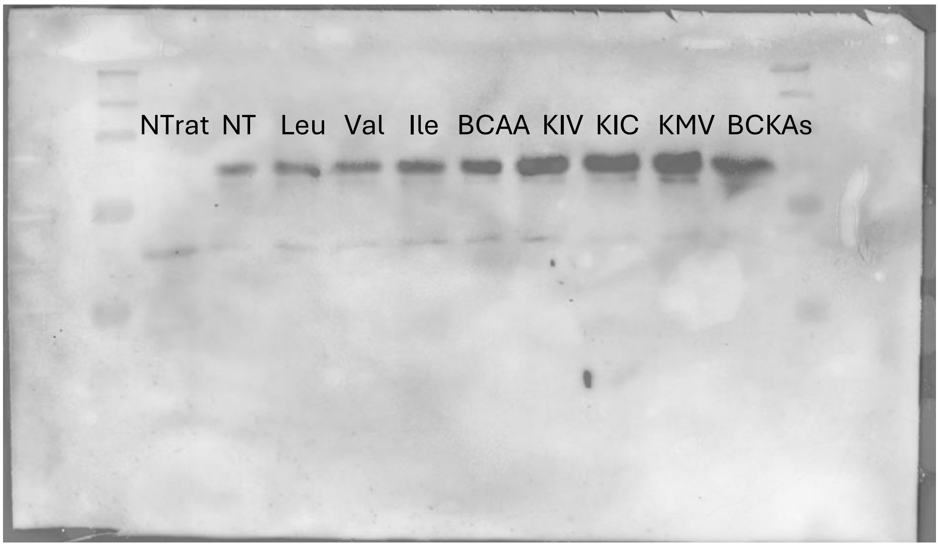


Alpha tubulin

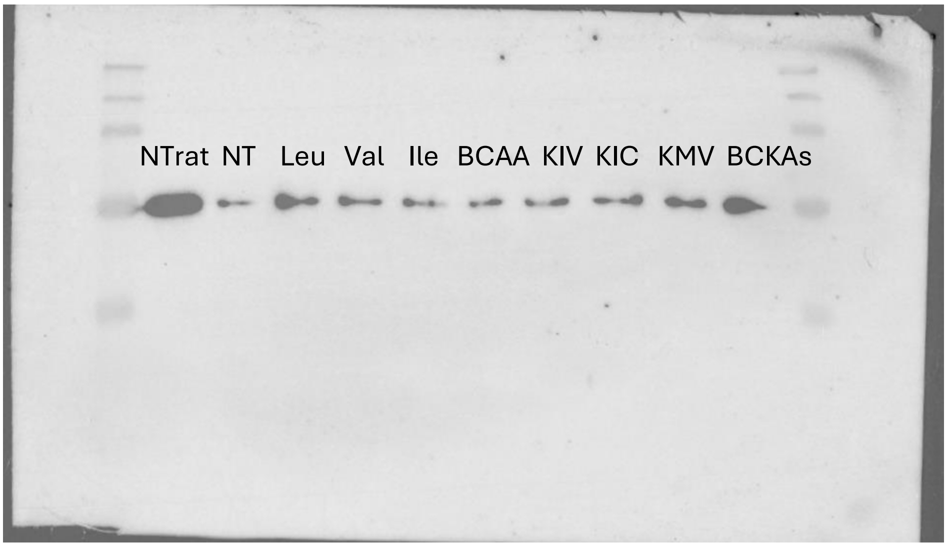


BCAT2

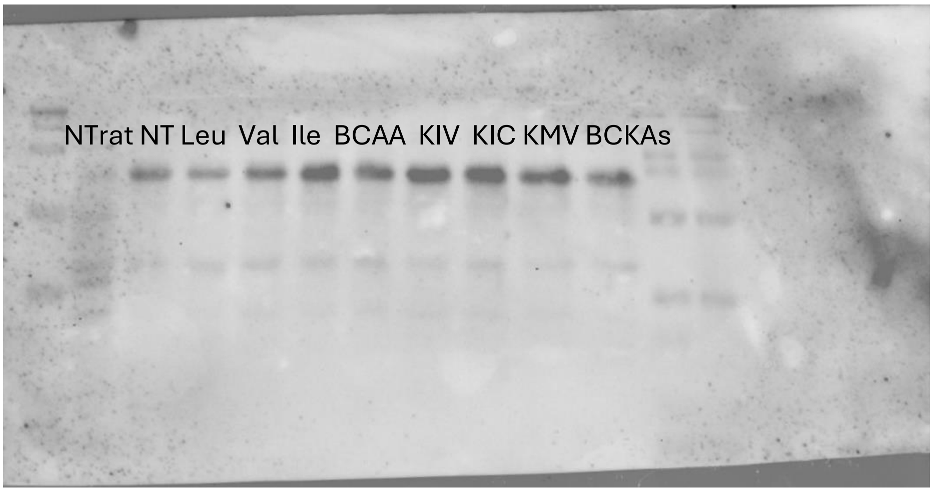


Alpha tubulin

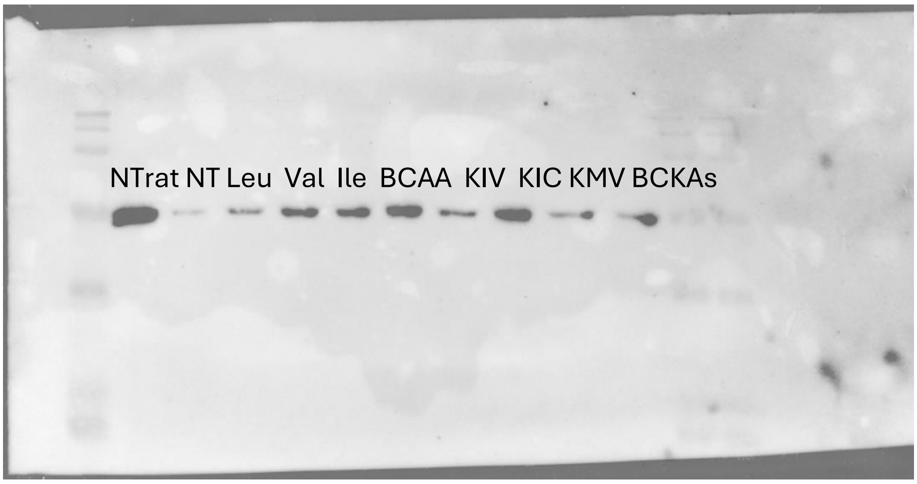


BCKDH

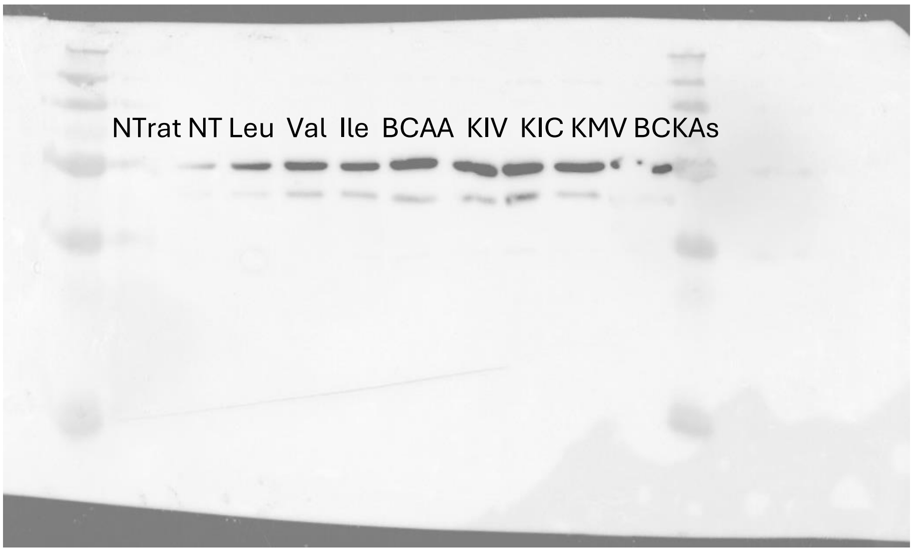


BCAT2

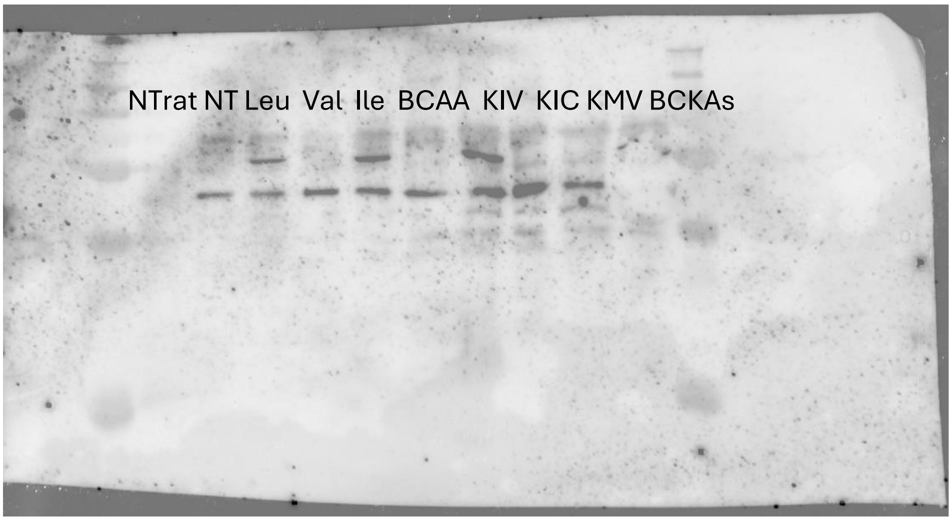


Alpha tubulin

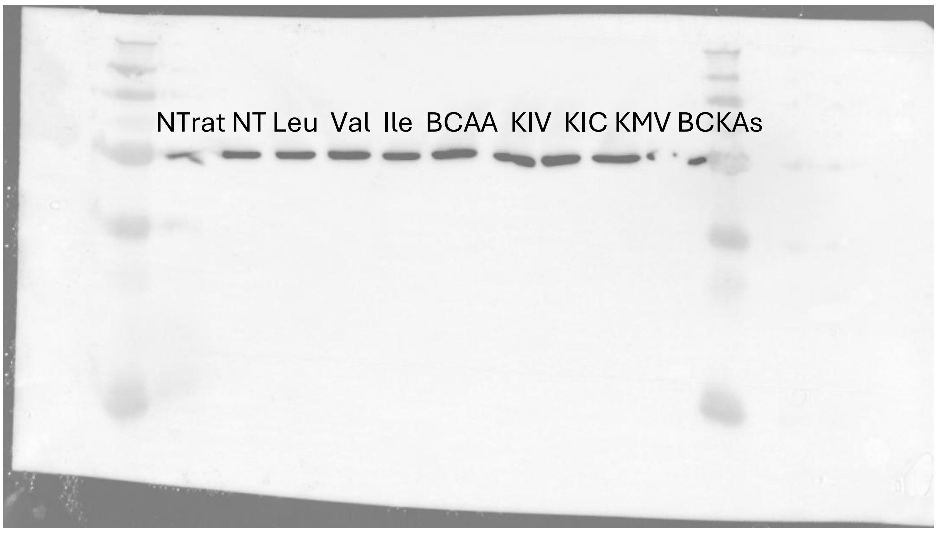


BCKDH

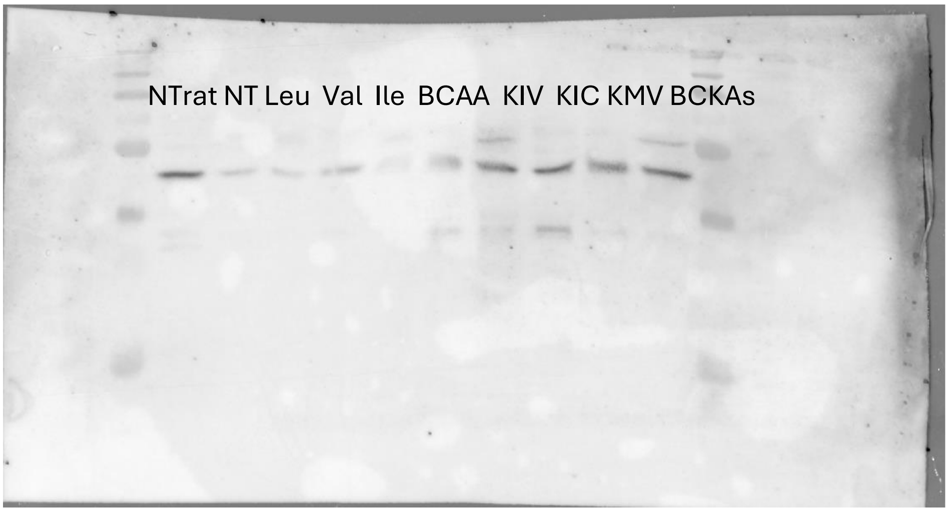


Alpha tubulin

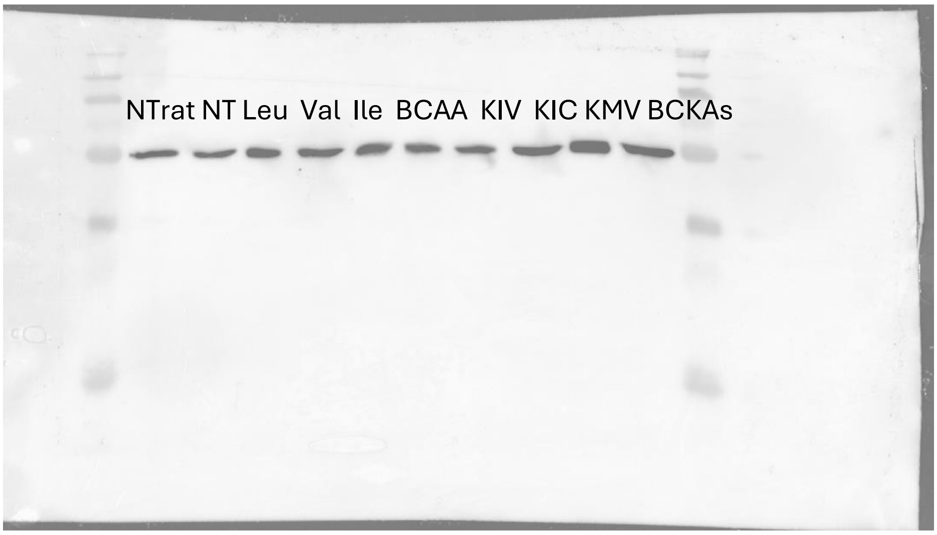


**Figure 4b:**

BCAT2

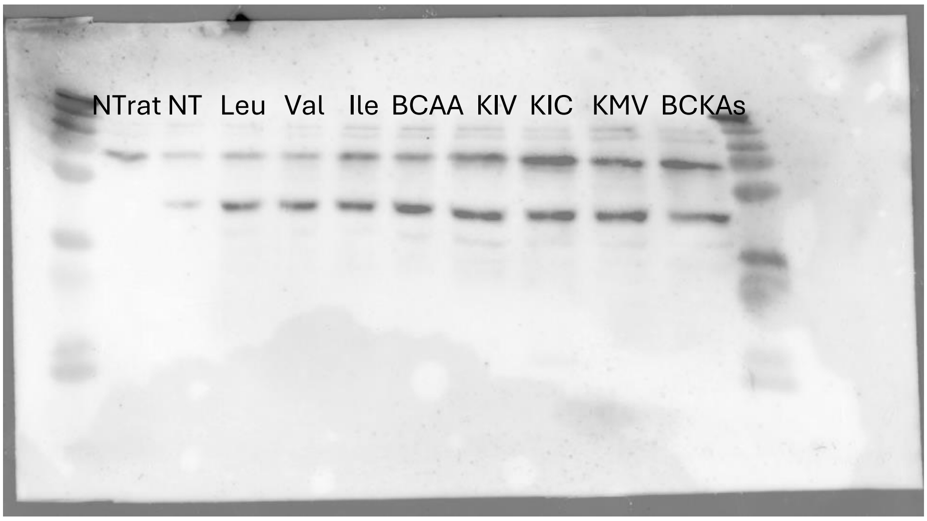


Alpha tubulin

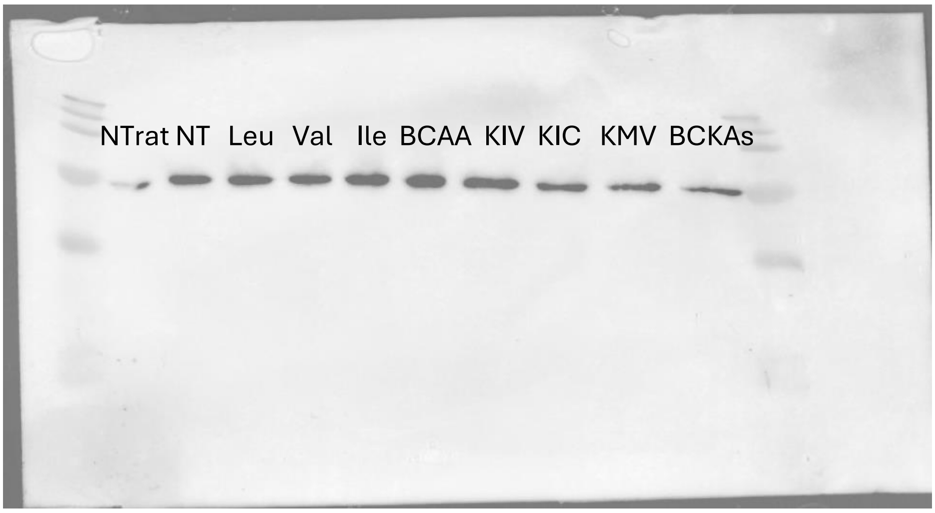


BCAT2

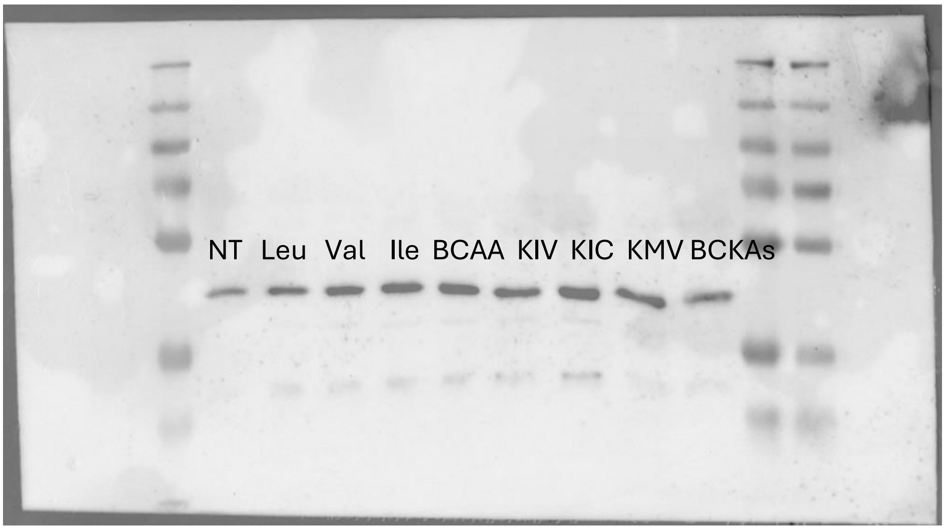


Alpha tubulin

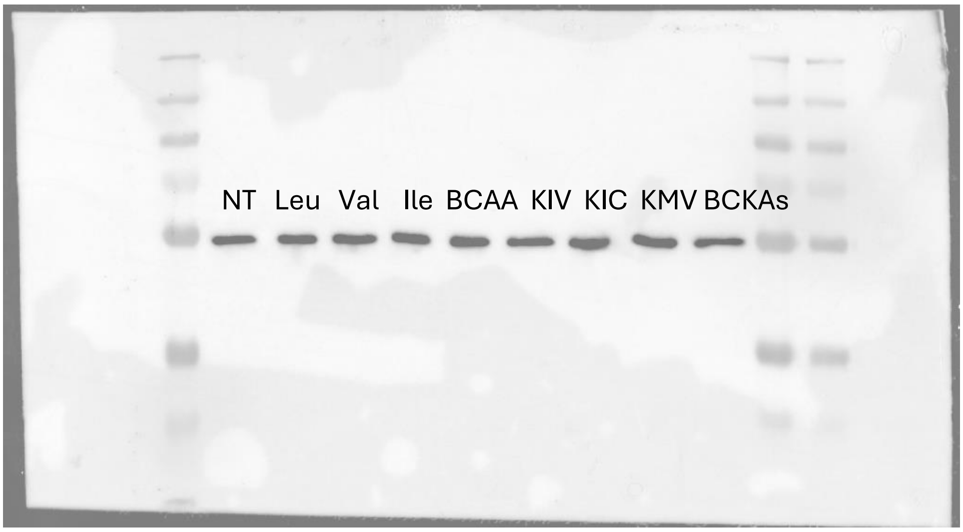


BCAT2

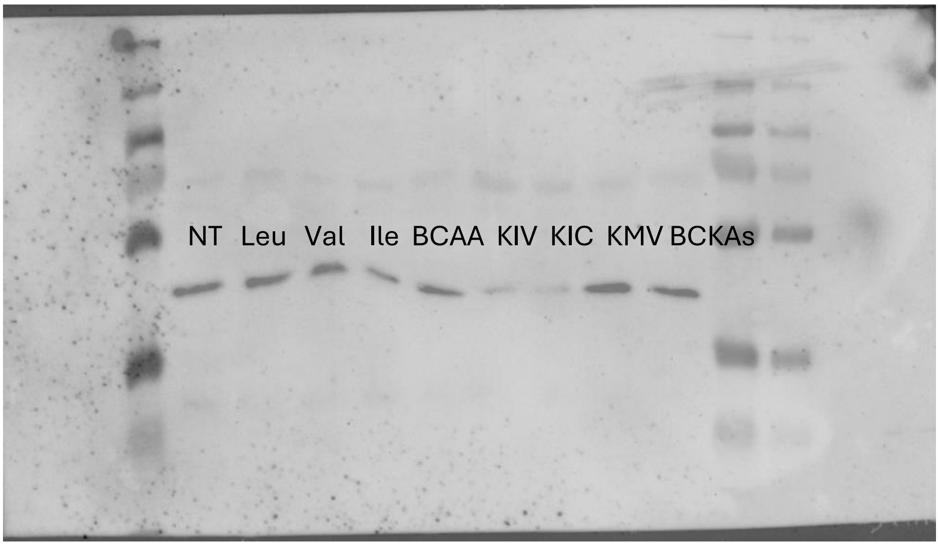


Alpha tubulin

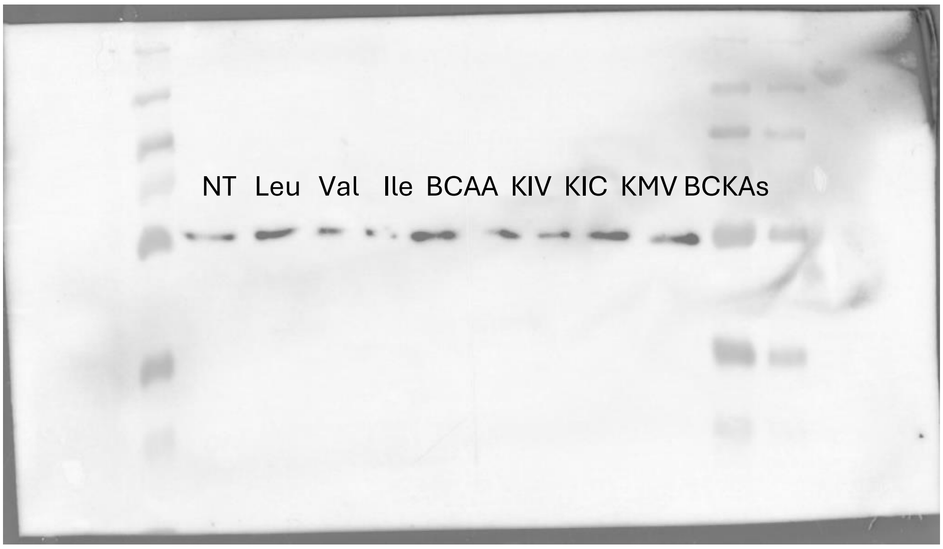


BCKDH

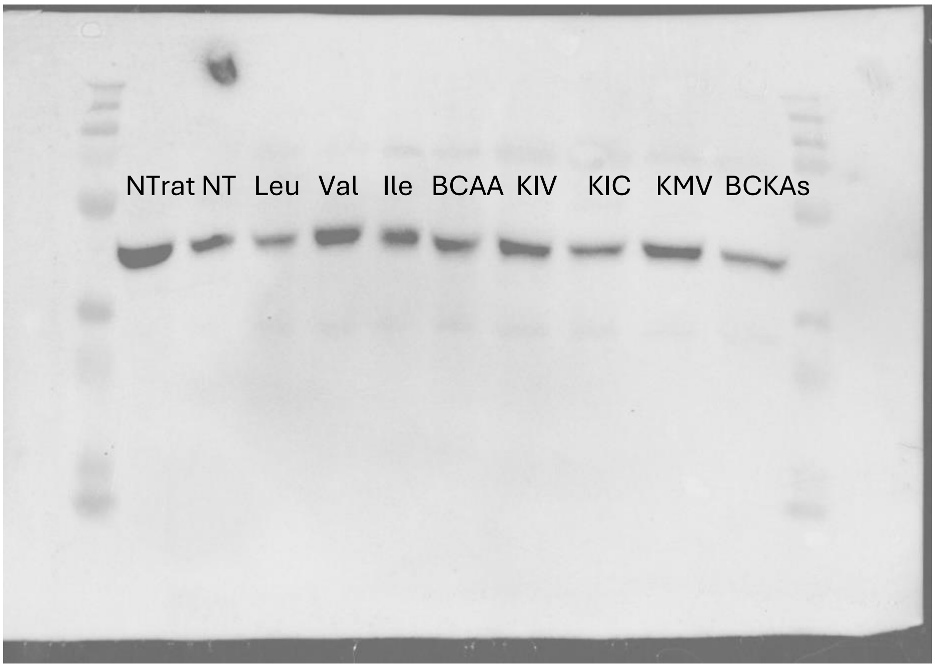


Alpha tubulin

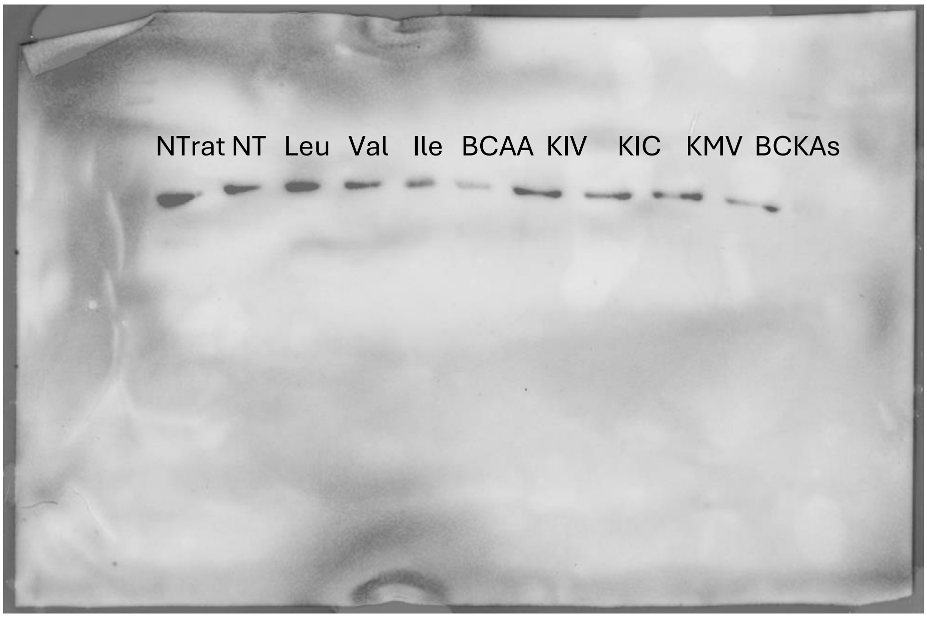


BCKDH

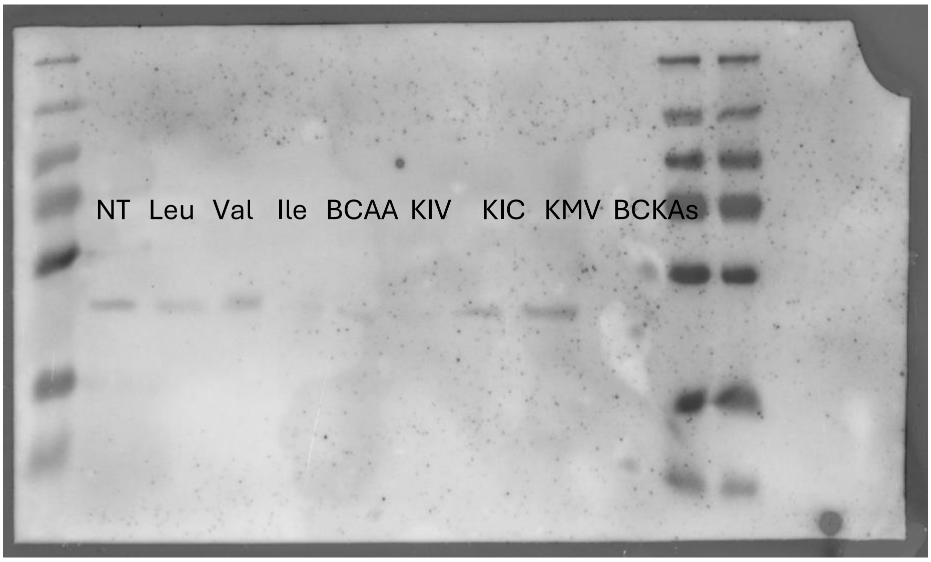


Alpha tubulin

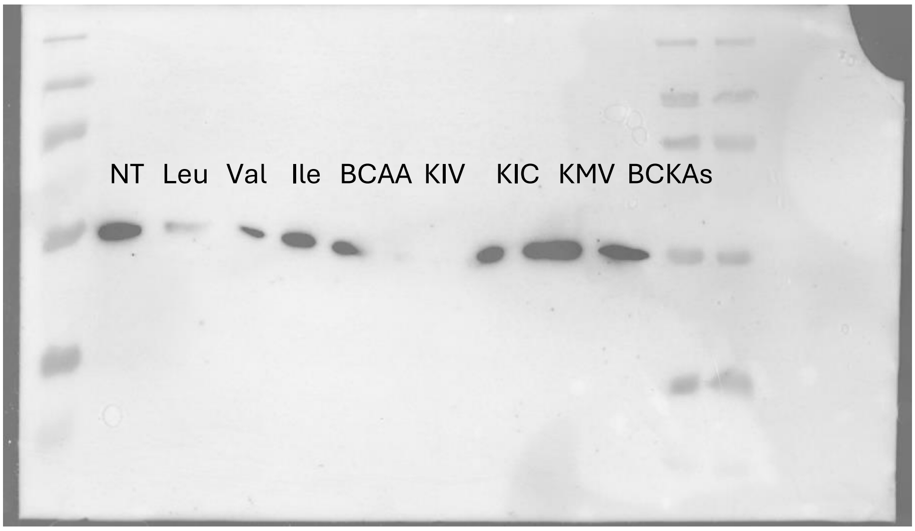


BCKDH

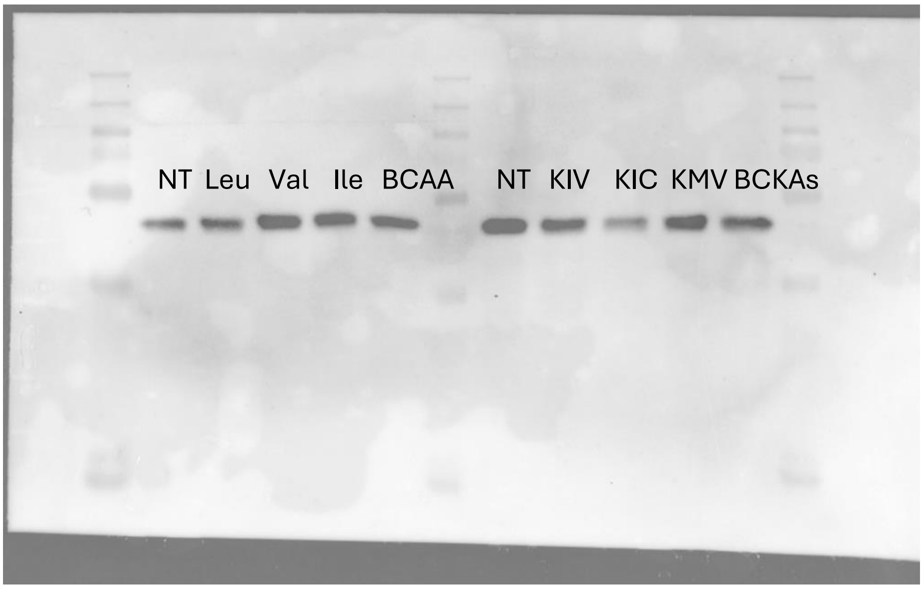


Alpha tubulin

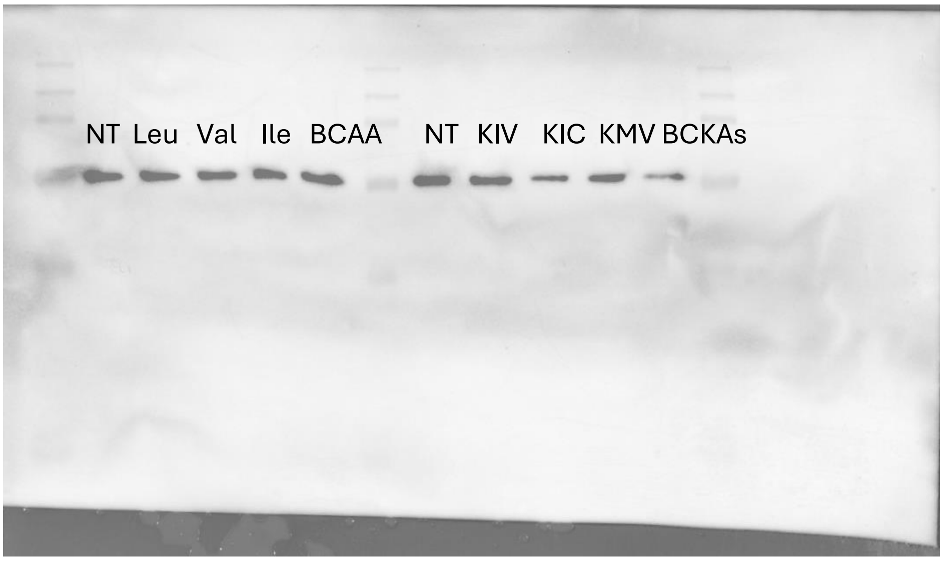


**Figure 5a:**

Collagen type 1

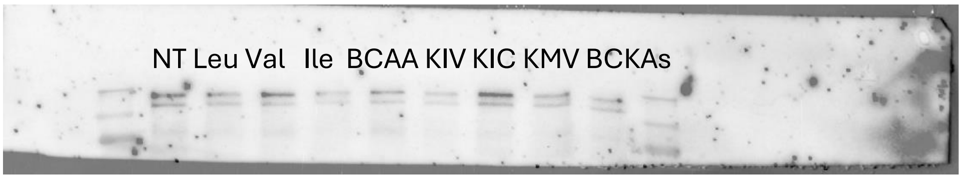


Alpha tubulin

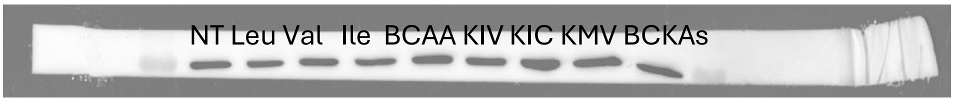


AlphaSMA

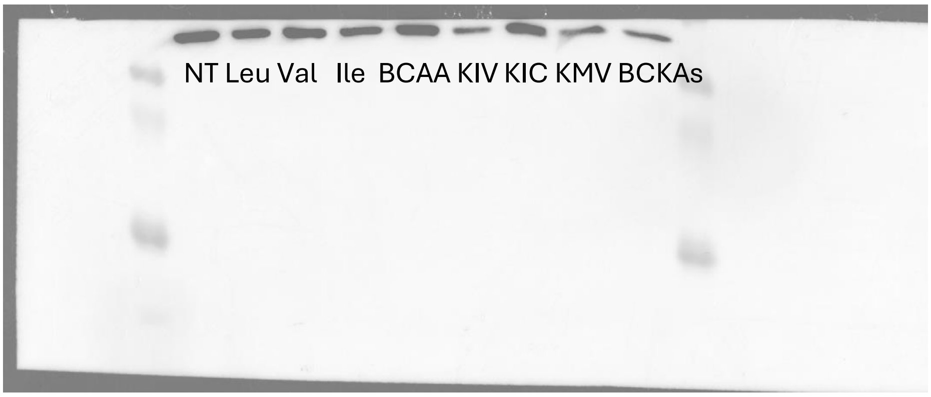

Collagen type 1

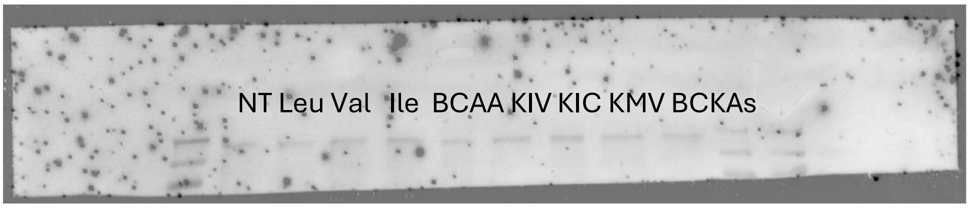


Alpha tubulin

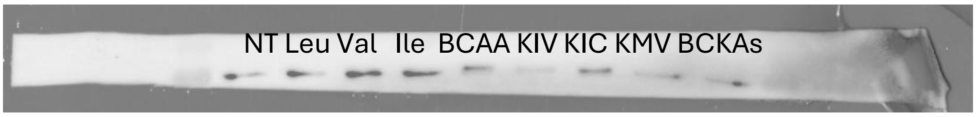


AlphaSMA

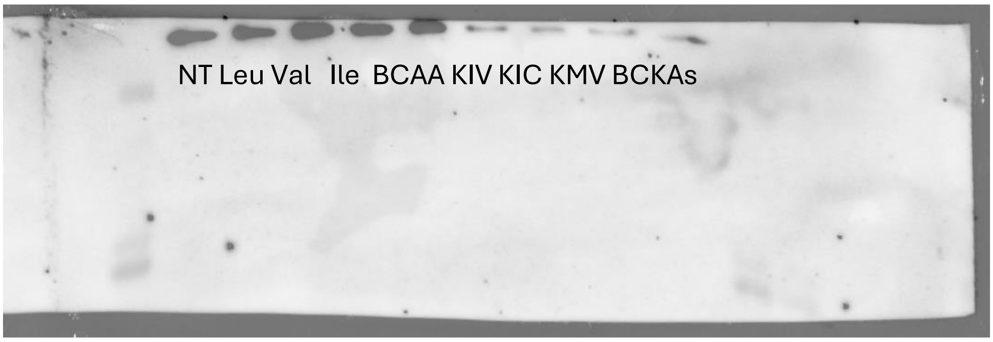


Collagen type 1

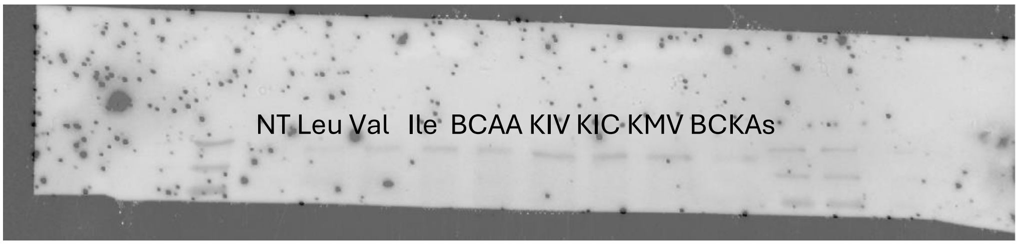


Alpha tubulin

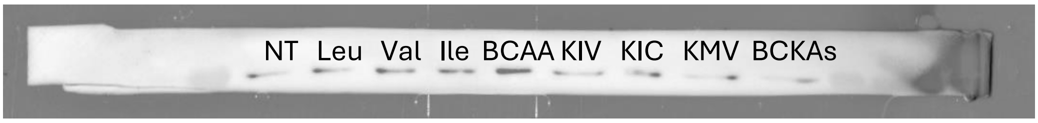

AlphaSMA

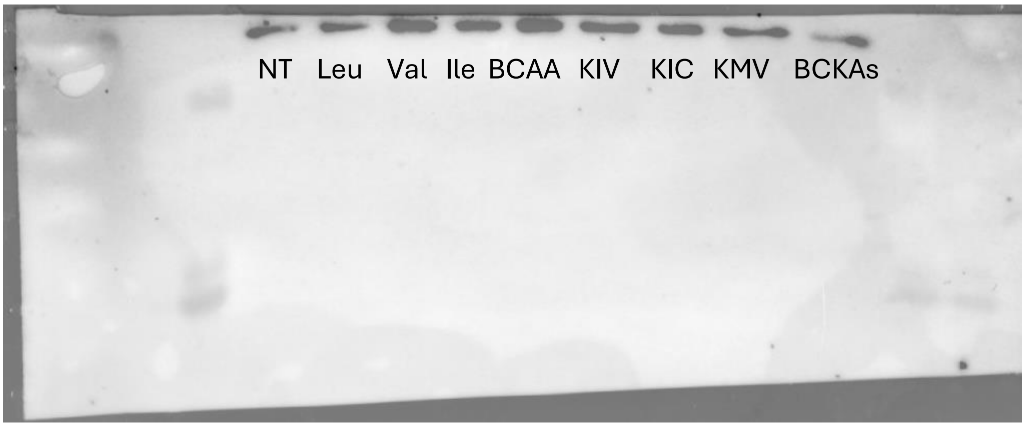


**Figure 6a:**

Collagen type 1

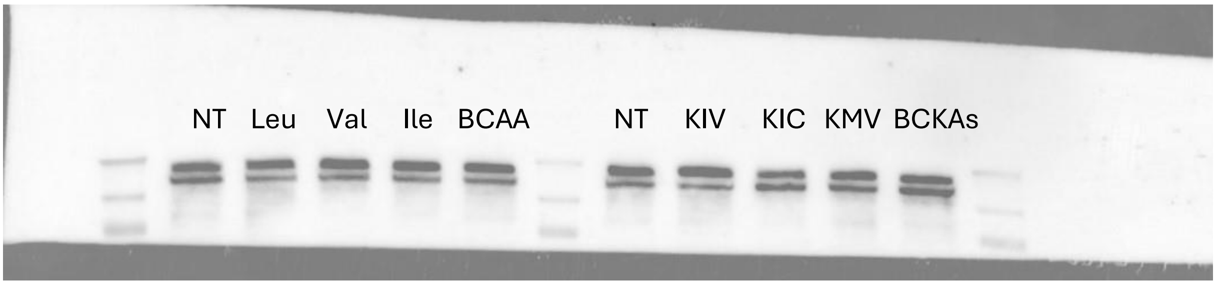


Alpha tubulin

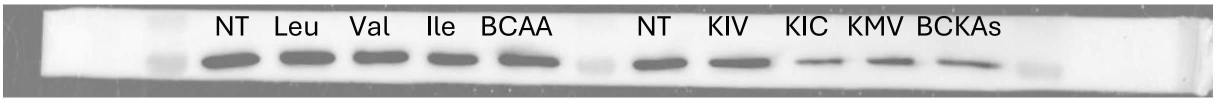


AlphaSMA

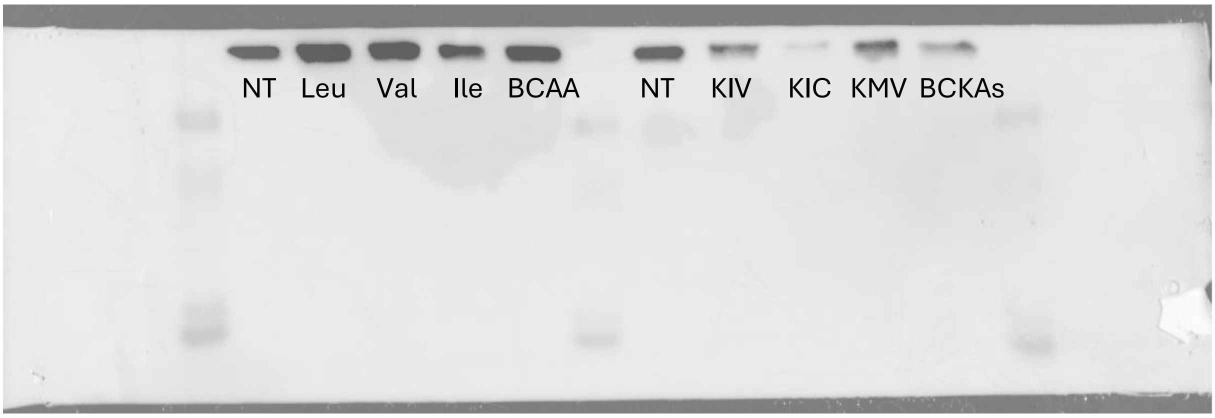


Collagen type 1

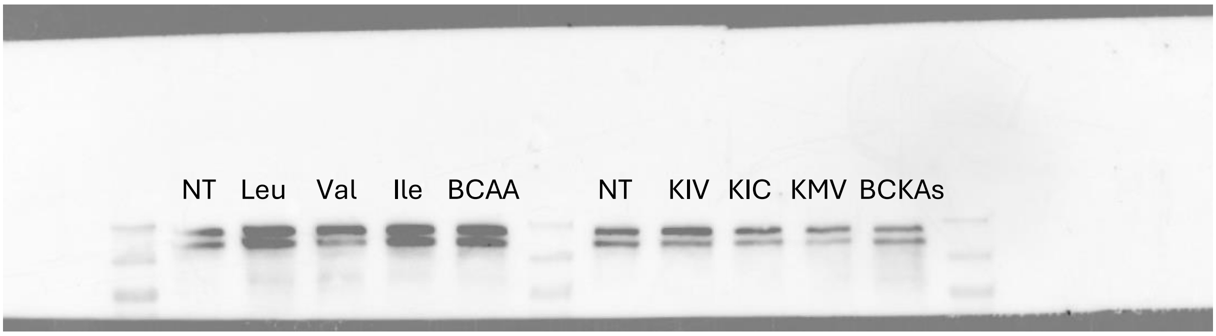


Alpha tubulin

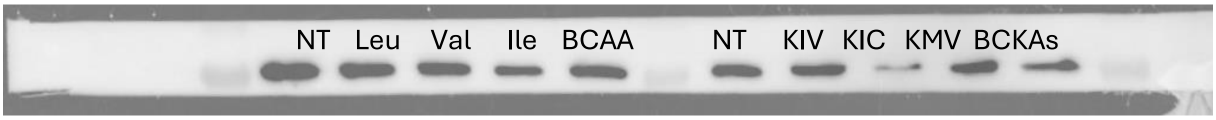


AlphaSMA

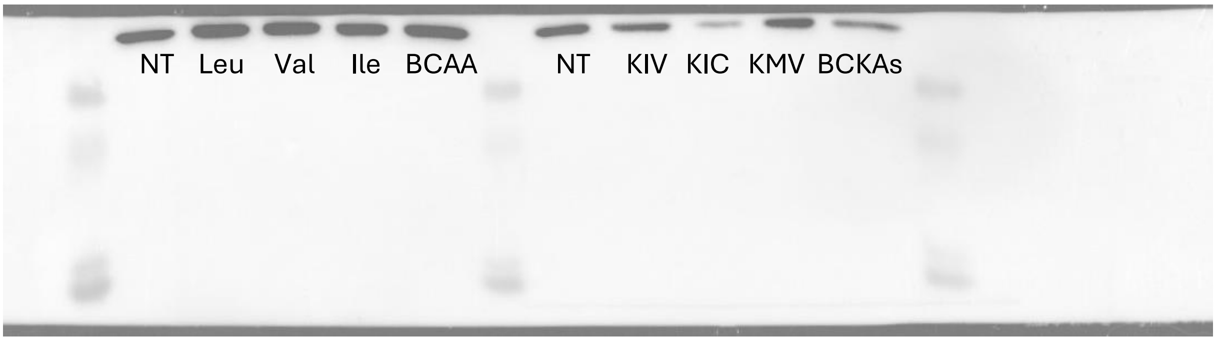


Collagen type 1

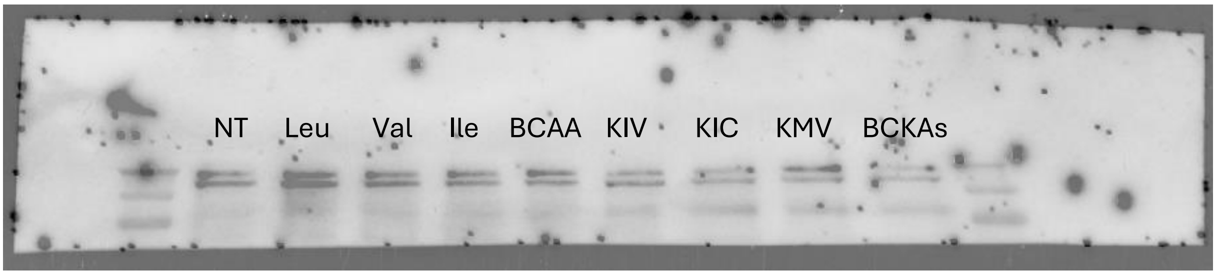


Alpha tubulin

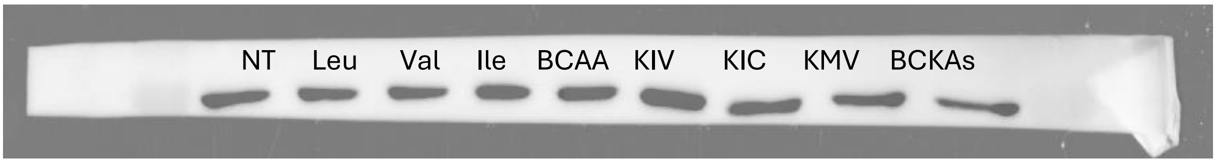


AlphaSMA

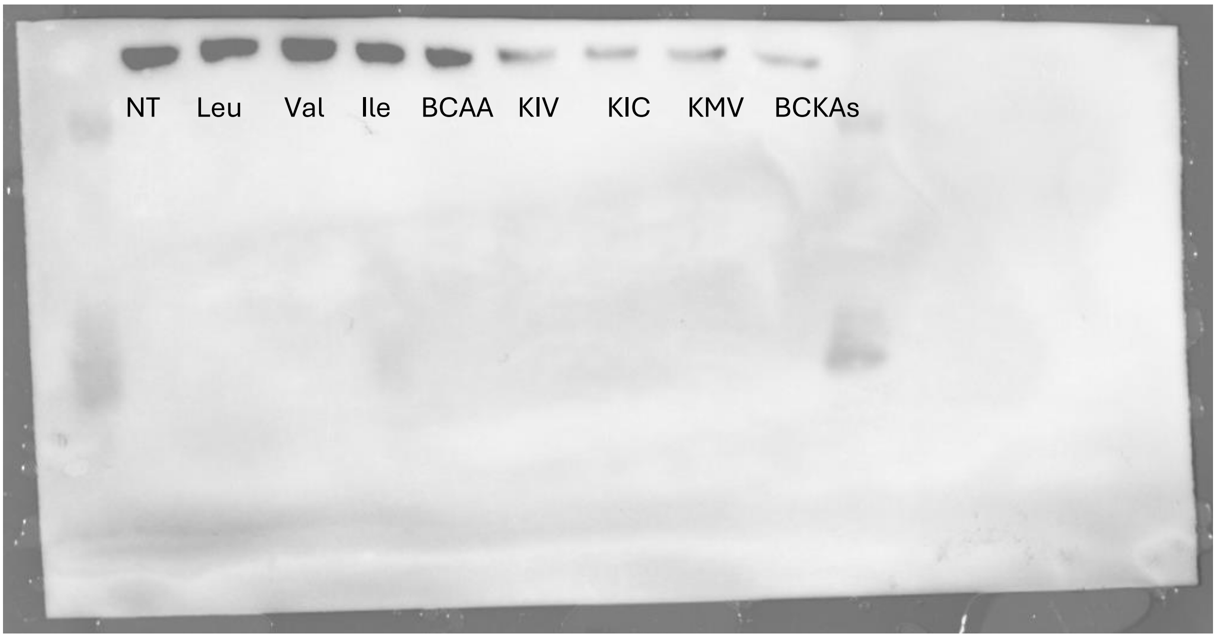


Alpha tubulin

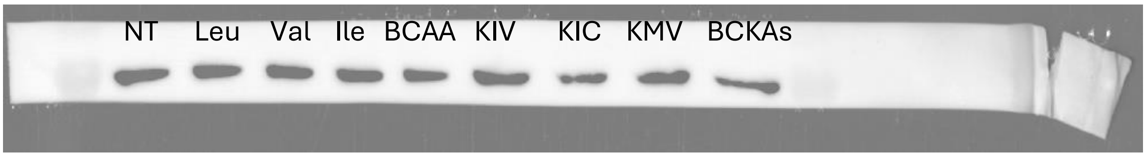

Supplement: Supplementary file 1 — Supplementary file1 (PDF 3446 KB) [file 11033_2024_10027_MOESM1_ESM.pdf]
